# Integrating single-cell sequencing and transcriptome analysis to unravel the mechanistic role of sialylation-related genes in sepsis-induced acute respiratory distress syndrome

**DOI:** 10.3389/fimmu.2025.1528769

**Published:** 2025-05-01

**Authors:** Xiaobing Liu, Yake Huang, Hao Zhang, Xia Yang, Quanxing Liu, Jigang Dai

**Affiliations:** ^1^ Department of Thoracic Surgery, Xinqiao Hospital, Army Medical University (Third Military Medical University), Chongqing, China; ^2^ Department of Obstetrics and Gynecology, Xinqiao Hospital, Army Medical University (Third Military Medical University), Chongqing, China; ^3^ Department of Critical Care Medicine, Daping Hospital, Army Medical University (Third Military Medical University), Chongqing, China; ^4^ Department of Wound Infection and Drug, State Key Laboratory of Trauma and Chemical Poisoning, Daping Hospital, Army Medical University (Third Military Medical University), Chongqing, China

**Keywords:** sepsis-induced acute respiratory distress syndrome, sialylation, nomogram, single-cell RNA sequencing, key genes

## Abstract

**Background:**

Studies have shown that sialylation of C1 esterase inhibitors is crucial for their interaction with histones, and histone-C1 esterase inhibitor complexes are detected in acute respiratory distress syndrome (ARDS), suggesting a potential role of sialylation in ARDS. However, the specific function of sialylation in ARDS remains unclear. Therefore, this study aimed to investigate the mechanism of sialylation-related genes (SRGs) in sepsis-induced ARDS.

**Methods:**

The ARDS related datasets (GSE32707, GSE66890, and GSE151263) were included in this study. Candidate genes were identified by implementing differential expression analysis and weighted gene co-expression network analysis (WGCNA). Subsequently, further selection by machine learning and expression assessment confirmed the key genes related to sialylation in sepsis-induced ARDS. Following this, the predictive ability of key genes as a whole for sepsis-induced ARDS was evaluated by creating a nomogram model. Afterwards, enrichment analysis, construction of regulatory networks, and drug prediction analysis were implemented to further understand the molecular mechanisms of action of key genes. Furthermore, single-cell RNA sequencing (scRNA-seq) data analysis was conducted to obtain key cells. Additionally, cell communication and pseudo-time analyses were implemented. In the end, the expression levels of the key genes were assessed by collecting clinical samples.

**Results:**

CD19 and GPR65 were identified as key genes associated with sialylation in sepsis-induced ARDS. The constructed nomogram model demonstrated that CD19 and GPR65 as a whole exhibited robust predictive capability for sepsis-induced ARDS. Meanwhile, CD19 and GPR65 were also found to be significantly co-enriched in the apoptosis and B-cell receptor signaling pathway. In addition, some important regulators and drugs with targeting effects on key genes were predicted, such as NEAT1, OIP5-AS1, alprostadil, and tacrolimus. Further, the scRNA-seq data analysis identified nine cell types, among which CD14 monocytes (CD14Mono) was designated as the key cell. Importantly, GPR65 expression exhibited dynamic changes during differentiation of CD14Mono. Also, we found that CD19 was significantly up-regulated in ARDS group.

**Conclusion:**

We identified CD19 and GPR65 as key genes associated with sialylation in sepsis-induced ARDS, highlighting CD14Mono as key cell type implicated in sepsis-induced ARDS. These findings offered theoretical support for understanding the mechanism of sialylation on sepsis-induced ARDS.

## 1 Introduction

Acute Respiratory Distress Syndrome (ARDS) is characterized by acute inflammatory lung injury, with histological features including diffuse alveolar damage, pulmonary edema, hyaline membrane formation, alveolar hemorrhage, and inflammation (1). ARDS constitutes 10% of intensive care unit admissions, with more than 3 million cases reported annually worldwide, and is associated with significant morbidity and mortality rates (2). While various triggers, including pneumonia, aspiration, trauma, pancreatitis, and multiple blood transfusions, can induce ARDS, sepsis remains the predominant cause, responsible for 32% of ARDS cases (3). The current treatment modalities for ARDS primarily encompass mechanical ventilation, pharmacological therapy with glucocorticoids, oxygen therapy, supportive care, and positional therapy, among other strategies (1). Despite advancements in mechanical ventilation therapy that have notably decreased ARDS mortality rates, the rates remain high at 25-40%, and there are currently no targeted treatments or specific key genes for critically ill patients (4, 5). Considering ARDS is a highly heterogeneous syndrome with variations contingent upon the underlying cause, the identification of specific key genes is essential for the diagnosis and treatment of sepsis-induced ARDS.

Sialylation, a post-translational modification, is critical in immune cell function and inflammatory responses (6). This process is regulated by sialyltransferases, transporters, and neuraminidases, and it plays a critical role in maintaining cell-cell interactions. It is also associated with numerous diseases, including cancer, embryonic demise, and immune system abnormalities (7). Research indicates that the sialylation of C1 esterase inhibitor is crucial for its interaction with histones, and this binding can mitigate the adverse effects of lung injury. Moreover, histone-C1 esterase inhibitor complexes have been identified in bronchoalveolar lavage fluid from ARDS patients and various lung injury models, suggesting a potential role for sialylation in ARDS (8). Additionally, the activity of sialidase NEU1 may modulate the sialylation status of angiotensin-converting enzyme 2 (ACE2) and other host receptors, as well as the extent of lysosomal exocytosis, thereby influencing the susceptibility, infectivity, and transmission of SARS-CoV-2 (7). Although sialylation has been shown to be associated with ARDS, the specific mechanism of sialylation in ARDS needs to be further investigated.

Single-cell RNA sequencing (scRNA-seq) has advanced ARDS research by profiling individual cell gene expression, revealing rare cell subsets, transitional states, and complex cell-cell communication networks (9). Ye et al. developed iMLGAM, a machine learning and genetic algorithm framework for predicting immunotherapy responses using multi-omics data (10). Other studies identified a plasma cell signature predicting immunotherapy outcomes (11, 12) and a T-cell exhaustion-related feature predicting chronic infection or tumor prognosiss (13) In ARDS patients, sepsis-related cases show increased CD14 cells, while pneumonia-related cases have more cytotoxic cells and NK T cells, indicating significant immune cell heterogeneity (14). scRNA-seq has demonstrated broad application potential and important value in ARDS research. It not only reveals the complexity and heterogeneity of immune cells in ARDS but also provides a scientific basis for the development of therapeutic strategies.

This study utilized transcriptome and single-cell sequencing data pertaining to sepsis-induced ARDS from the GEO database to identify key genes associated with sialylation in sepsis-induced ARDS using a suite of bioinformatics approaches. Additionally, enrichment analysis, regulatory network construction, and drug prediction were performed to elucidate the mechanisms of action of these key genes in sepsis-induced ARDS. Furthermore, based on the cellular expression of key genes, critical cell types were identified, and a pseudo-time series analysis was conducted on these cells to assess the expression patterns of key genes throughout various stages of differentiation. The ultimate aim is to offer novel insights and references for the clinical management of sepsis-induced ARDS.

## 2 Materials and methods

### 2.1 Data extraction

ARDS-related transcriptome sequencing data (GSE32707 and GSE66890) and single-cell RNA sequencing (scRNA-seq) data (GSE151263) were gained by accessing the Gene Expression Omnibus (GEO) database (http://www.ncbi.nlm.nih.gov/geo/), which were applied in this study. The GSE32707 dataset, which obtained based on the sequencing platform GPL10558, contained 144 blood samples, of which 18 blood samples from patients with sepsis-induced ARDS and 30 blood samples from patients with sepsis were selected for inclusion in this study. In particular, these blood samples were collected on the day of admission (day 0). The GSE66890 dataset, obtained based on the sequencing platform GPL6244, comprised 62 blood samples. Of these, 28 blood samples from patients with sepsis and 29 blood samples from patients with sepsis-induced ARDS were included in this study. Specifically, GSE32707 dataset was utilized as the training set while GSE66890 dataset was served as the validation set. The GSE151263 dataset consisted of peripheral blood mononuclear cell (PBMC) samples from three patients with sepsis-induced ARDS and four patients with sepsis, which were acquired based on sequencing platform GPL20301. Additionally, a total of 110 sialylation related genes (SRGs) were obtained through accessing Molecular Signatures Database (MSigDB) (https://www.gsea-msigdb.org/gsea/msigdb) (6).

First, the gene expression matrix was obtained through geoChina in the AnnoProbe package (v0.1.7). Subsequently, the obtained gene expression matrix was examined to check whether log2-standardization was required for it, so as to ensure the consistency and comparability of the data. If in the gene expression matrix, the 99th percentile was greater than 100, and the difference between the maximum and minimum values was greater than 50, while the first quartile (qx[2]) was greater than 0, and the first quartile was between 0 and 1, and the third quartile was between 1 and 2, then log2-standardization was carried out. Next, the corresponding GPL file was used for gene annotation operations in order to accurately identify the genes. Finally, the pre - processed data was saved and used for subsequent analysis.

### 2.2 Differential expression analysis

With the application of the limma package (v 3.54.0) (15), differential expression analysis was implemented between ARDS and sepsis in the GSE32707 dataset with the aim of identifying differentially expressed genes (DEGs) [*P* < 0.05 &|Log_2_ Fold Change (FC)| > 0.5]. In order to understand the distribution of DEGs as a whole, the ggplot2 package (v 3.4.1) (16) was employed to create a volcano plot, and the top 10 up-regulated and down-regulated genes sorted by log_2_FC were marked in the volcano plot. Subsequently, the ComplexHeatmap package (v 2.15.1) (17) was utilized to draw a heat map of the expression for these 20 DEGs.

### 2.3 Weighted gene co-expression network analysis

Based on the SRGs as the background gene set, the single-sample gene set enrichment analysis (ssGSEA) algorithm of the GSVA package (v 1.42.0) (18) was utilized to calculate the ssGSEA score for each sample in the GSE32707 dataset, followed by comparing the difference of these scores between the ARDS and sepsis groups by Wilcoxon test (*P* < 0.05).

Key module genes linked to SRGs were gained with the adoption of WGCNA using WGCNA package (v 1.70-3) (19). First, clustering analysis was adopted on all samples in the GSE32707 dataset. Through the clustering of the samples, it was determined whether there were outliers that needed to be filtered out to ensure the accuracy of the subsequent analysis. To ensure that the inter-gene interactions maximally conformed to the scale-free distribution, a soft threshold was determined for the data. The optimal soft threshold (β) was determined when the scale free topology model fit (R^2^) exceeded the threshold value of 0.85 and the mean value of the neighborhood function also gradually approached 0. Based on the determined optimal soft threshold (β), the minimum number of genes per gene module was set to 50 in accordance with the criteria of the hybrid dynamic tree cutting, thus clustering genes into different modules. The ssGSEA score of SRGs was used as a phenotypic trait, followed by calculating the correlation coefficient between the module and this score by Pearson correlation analysis. Modules with significant maximum positive and negative correlations were selected and defined as key modules [|correlation coefficient (cor)| > 0.3 & *P* < 0.05]. The genes in these key modules were defined as the key module genes.

### 2.4 Identification and functional analysis of candidate genes

The intersection of DEGs and key module genes was taken using VennDiagram package (v 1.7.1) (20) to gain genes linked to both sepsis-induced ARDS and sialylation, which were recorded as candidate genes. The signaling pathways associated with the candidate genes were investigated using Kyoto Encyclopedia of Genes and Genomes (KEGG) enrichment analysis via ClusterProfiler package (v 4.2.2) (21). A significance level of *P* < 0.05 was employed to determine the enrichment of these candidate genes in the signaling pathway. Subsequently, these candidate genes were entered into the Search Tool for the Retrieval of Interacting Genes/Proteins (STRING) database (http://www/string-db.org/), followed by the construction of a protein-protein interactions (PPI) network with the objective of probing their interactions at the protein level (medium confidence = 0.4).

### 2.5 Screening candidate key genes through three machine learning algorithms

Three machine learning algorithms were executed for these candidate genes to further confirm the candidate biomarkers, which comprised least absolute shrinkage and selection operator (LASSO), Boruta, and XGBoost algorithms. The LASSO regression analysis was performed using the glmnet package (v 4.1-4), with the parameter setting of family=binomial (22). Then, 10-fold cross-validation was applied, and the L1-penalty (lambda) was used to shrink less important genes to zero. The error rate was calculated for each lambda value, and the optimal lambda was identified. The genes whose regression coefficients were not penalized to zero were selected as the more important feature genes for the disease, and the best classification model was constructed. Boruta was a feature selection algorithm implemented through the Boruta package (v 7.0.0) (23), which randomly perturbed the order of each gene and evaluated the importance of each gene. Then, correlation screening was performed with the pValue parameter set to 0.01 to determine the relevance of the genes. The maximum number of iterations was set to 300, and the algorithm continued to screen and remove genes with lower relevance. Finally, when the iteration reached the maximum number of steps or other stopping criteria were met, such as no more genes being marked as lowly correlated, the remaining genes were considered the optimal feature genes. Next, the XGBoost algorithm was performed using the xgboost package (v 1.7.3.1) (24). The maximum number of iterations was set to 25, and eta was set to 0.3 to control the step size of weight updates during each iteration. Based on this, the model was trained, and the feature genes that made significant contributions to the model were identified by evaluating their importance as output by the algorithm. Furthermore, candidate key genes were obtained by taking the intersection of the feature genes selected by these three machine learning algorithms.

### 2.6 Identification of key genes and evaluation of their predictive ability for sepsis-induced ARDS

The expression levels of candidate key genes were evaluated between the ARDS and sepsis groups in both GSE32707 and GSE66890 datasets. Candidate key genes with consistent expression trends in both datasets and significant differences in gene expression levels between ARDS and sepsis groups were selected and defined as key genes for subsequent analysis (*P* < 0.05). Subsequently, the distribution of key genes was interrogated by implementing chromosomal localization and subcellular localization analyses. The difference was that the chromosomal localization analysis was performed using the RCircos package (v 1.2.2) (25), while the subcellular localization analysis was conducted using the Cell-PLoc 3.0 website (http://www.csbio.sjtu.edu.cn/bioinf/Hum-mPLoc3/). Afterwards, in order to determine whether the identified key genes were accurate for the prediction of sepsis-induced ARDS patients, the rms package (v 6.5-0) (26) was employed to construct a nomogram model of key genes in GSE32707 dataset. In the nomogram model, the key genes were scored separately, with each score corresponding to a specific key gene. The sum of the scores of these key genes determined the total point, which was then utilized to infer the incidence of sepsis-induced ARDS. Moreover, calibration curve and decision curve analysis (DCA) were adopted to evaluate the accuracy of the predictive capability of the nomogram model. Notably, calibration curve was plotted using rmda (v 1.6) (https://CRAN.R-project.org/package=rmda) and DCA was implemented employing ggDCA package (v 1.2) (https://rdrr.io/github/yikeshu0611/ggDCA/).

### 2.7 Functional enrichment analysis

Gene set enrichment analysis (GSEA) was implemented in GSE32707 dataset to reveal the signaling pathways with significant enrichment of key genes. First, ARDS samples were categorized into high and low expression groups based on the median expression of key genes. Then, the high and low expression groups were subjected to differential expression analysis to identify DEGs (high *vs* low) and their corresponding log_2_FC. After that, these DEGs were sorted based on their log_2_FC, followed by conducting GSEA via ClusterProfiler package on the sorted DEGs (*P.*adjust < 0.05). The reference gene set utilized in this analysis was ‘c2.cp.kegg.v7.5.1.symbols.gmt’, which was gained from MSigDB. Besides, the GeneMANIA database (http://genemania.org) was applied to predict genes associated with key gene functions and the functions they were involved in.

### 2.8 Construction of regulatory networks and analysis of drug prediction

Regulatory factors that had regulatory relationships with key genes were predicted through the application of multiple databases with the objective of probing the regulatory mechanisms of key genes. Initially, the miRDB database (https://mirdb.org/) was employed for the prediction of microRNAs (miRNAs) that were regulatory factors of mRNAs, thereby obtaining pairs of miRNA-mRNA relationships. Subsequently, Encyclopedia of DNA Elements (ENCODE) database (https://www.encodeproject.org/) was utilized to predict long non-coding RNAs (lncRNAs) that exhibited regulatory associations with these identified miRNAs, resulting in the acquisition of pairs of miRNA-lncRNA relationships. By integrating the obtained sets of miRNA-mRNA and miRNA-lncRNA relationship pairs, a comprehensive lncRNA-miRNA-mRNA regulatory network was constructed and visualized using Cytoscape software (v 3.8.2) (27). Additionally, the Drug Signatures database (DSigDB) (https://dsigdb.tanlab.org/DSigDBv1.0/) was employed to predict drugs targeting key genes to explore the potential therapeutic effects of these drugs on sepsis-induced ARDS.

### 2.9 scRNA-seq data analysis

The scRNA-seq data analysis was performed in GSE151263 dataset to probe the expression of key genes at the cellular level. First, the scRNA-seq data were filtered using Seurat package (v 5.0) (28) to filter out cells with less than 300 genes and genes covered by less than 5 cells, followed by retaining the genes and cells that satisfied the following conditions, which contained 200 < nFeature < 3,000, nCount < 20,000, and mitochondrial percentage < 5%. Subsequently, multiple samples were integrated using IntegrateData and the filtered data were normalized using NormalizeData from the Seurat package, followed by the identification of 2,000 highly variable genes using the FindVariableFeatures function. Immediately following this, principal component analysis (PCA) was implemented to assess the distribution of 2,000 highly variable genes in the ARDS and sepsis groups. The data were normalized using the ScaleData function in Seurat package, and statistically significant principal components (PCs) were determined using the JackStrawPlot function. Afterwards, the cells were clustered using the Uniform manifold approximation and projection (UMAP) method (resolution = 0.4). Moreover, the obtained cell subpopulations were annotated using the singleR package (v 1.0.6) (29) to identify specific cell types. By the way, the distribution of annotated cell types in the ARDS and sepsis groups was visualized.

### 2.10 Cell communication and pseudo-time analyses

The CellChat package (v 1.6.1) (30) was employed for cell communication analysis in annotated cell types. Following the creation of cell chat objects, importation of ligand receptor data in CellChatDB.human, and preprocessing, cell communication networks were generated. Heat map and circle plot were utilized to visually represent the number and weight of interactions between different cell types, while bubble plot was constructed to demonstrate the probability of communication regulated by specific ligand-receptor pairs from certain cell populations to other cellular groups. Subsequently, the expression of key genes in different cell types was demonstrated by UMAP, followed by implementing Wilcoxon test to assess the differences in key gene expression between different cell types in ARDS and sepsis groups (*P* < 0.05). Cells with significant expression of key genes in both cell types between the two groups were selected and defined as key cells. Further, pseudo-time analysis was implemented on the key cells using the monocle package (v 2.22.0) (31) with the objective of exploring their differentiation status and the changes in the expression of key genes during their differentiation stages.

### 2.11 Reverse transcription quantitative polymerase chain reaction

To further verify whether the key genes identified through bioinformatics analysis exhibit consistent expression patterns in clinical samples, the 5 sepsis-induced ARDS blood samples were collected in Daping Hospital, Army Medical University. The blood samples obtained from 5 sepsis patients were utilized as control samples. These blood samples were utilized to perform RT-qPCR. This study was approved by the Ethics Committee of the Daping Hospital, Army Medical University, Chongqing, China (#2019-112). All patients had signed an informed consent form. Total RNA of 10 samples was separated by the TRIzol (Ambion, Austin, USA) based on the manufacturer’s guidance. The inverse transcription of total RNA into cDNA was conducted using the SureScript-First-strand-cDNA-synthesis-kit (Servicebio, Wuhan, China) based on the producer’s indication. Subsequently, qPCR was carried out utilizing the 2xUniversal Blue SYBR Green qPCR Master Mix (Servicebio, Wuhan, China) under the direction of the manual. The primer sequences for PCR were tabulated in (**Supplementary Table 1**). The expression was uniformized to the internal reference GAPDH and computed employing the 2^−ΔΔCt^ method (32).

### 2.12 Statistical analysis

Based on R software (v 4.2.2), the data were analyzed. The Wilcoxon test was utilized to assess the differences between different groups. The *P* value less than 0.05 was considered statistically significant.

## 3 Results

### 3.1 Recognition of DEGs and key module genes linked to SRGs

**Figure 1:**
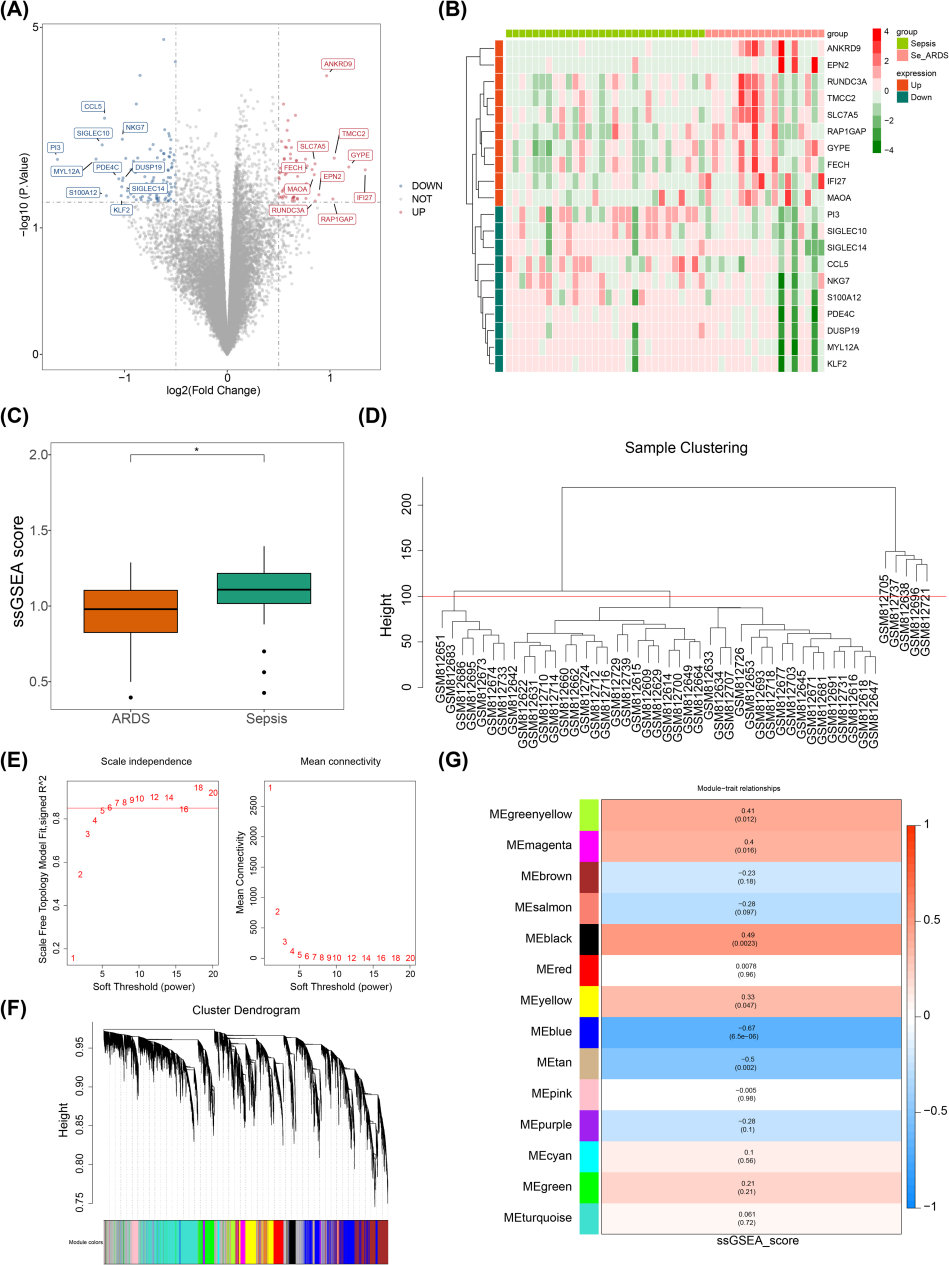
Recognition of DEGs and key module genes linked to SRGs. *, p < 0.05; **(A)** Volcano plot display of differentially expressed genes; **(B)** Heatmap display of differentially expressed genes; **(C)** Box plot of SRGs scores; **(D)** Sample clustering diagram; **(E)** Determination of soft threshold in WGCNA algorithm; **(F)** Cluster dendrogram; **(G)** Heatmap of the relationship between gene modules and traits.

With the application of the limma package, 166 DEGs were selected in the GSE32707 dataset. Among them, 64 genes were notably up-regulated in the ARDS group, while 102 genes were notably down-regulated in the ARDS group (**Figures 1A, B**). The Wilcoxon test demonstrated that the ssGSEA score of SRGs was significantly down-regulated in the ARDS group compared to the sepsis group, suggesting that sialylation does have an effect on sepsis-induced ARDS (**Figure 1C**). Subsequently, key module genes linked to SRGs were gained through WGCNA. The clustering analysis results indicated that outlier samples were identified using a height of 100, resulting in the elimination of five samples classified as outliers (**Figure 1D**). Immediately thereafter, the optimal soft threshold (β) was chosen to be six based on the criteria that R^2^ exceeded 0.85 and the mean value of the adjacency function gradually approached zero (**Figure 1E**). After that, 14 gene modules were gained (**Figure 1F**). Furthermore, Meblue and Meblack were selected as key modules due to the fact that Meblue (cor = -0.67, *P* = 6.5 × 10^-6^) and Meblack (cor = 0.49, *P* = 0.0023) exhibited significant maximum positive and negative correlations with ssGSEA scores, respectively (**Figure 1G**). In these two key modules, 2,784 key module genes were obtained.

### 3.2 Identification of seven candidate key genes

**Figure 2:**
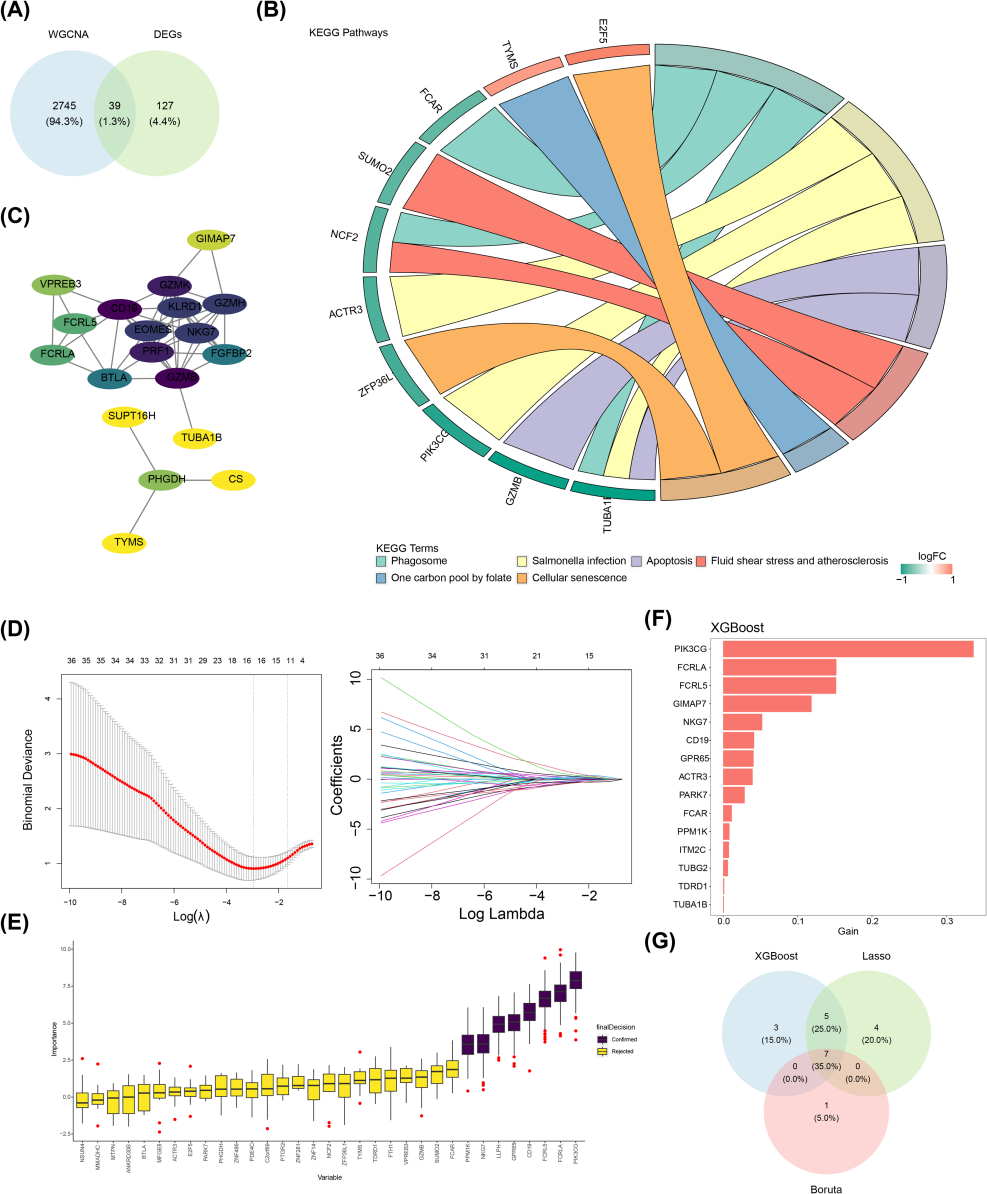
Identification of seven candidate key genes. **(A)** Candidate genes identification; **(B)** KEGG enrichment chord diagram; **(C)** Candidate genes PPI network; **(D)** Screening candidate key genes using LASSO regression analysis; **(E)** Boruta algorithm for identifying candidate key genes; **(F)** XGBoost for assessing the importance of feature genes in screening; **(G)** Venn diagram related to core genes.

A total of 39 candidate genes were identified through crossing 166 DEGs and 2,784 key module genes (**Figure 2A**). KEGG results indicated that these candidate genes were remarkably enriched to six signaling pathways, including phagosome, apoptosis, and cellular senescence (*P* < 0.05) (**Figure 2B**). After excluding the discrete proteins, a PPI network comprising 19 nodes and 51 edges was generated. Notably, candidate genes such as CD19, GZMK, KLRD1, and EOMES exhibited enhanced interactions with other genes within this network (**Figure 2C**). When the lambda in the LASSO analysis was 0.0518339, 16 feature genes were identified (**Figure 2D**). Meanwhile, eight and 15 feature genes were identified by Boruta (**Figure 2E**) and XGBoost algorithms (**Figure 2F**), respectively. Furthermore, seven candidate key genes were identified by overlapping three parts of the feature genes obtained through these three machine learning algorithms, which contained PIK3CG, FCRLA, FCRL5, NKG7, CD19, GPR65, and PPM1K (**Figure 2G**).

### 3.3 The key genes with different distribution exhibited excellent predictive ability for sepsis-induced ARDS

**Figure 3:**
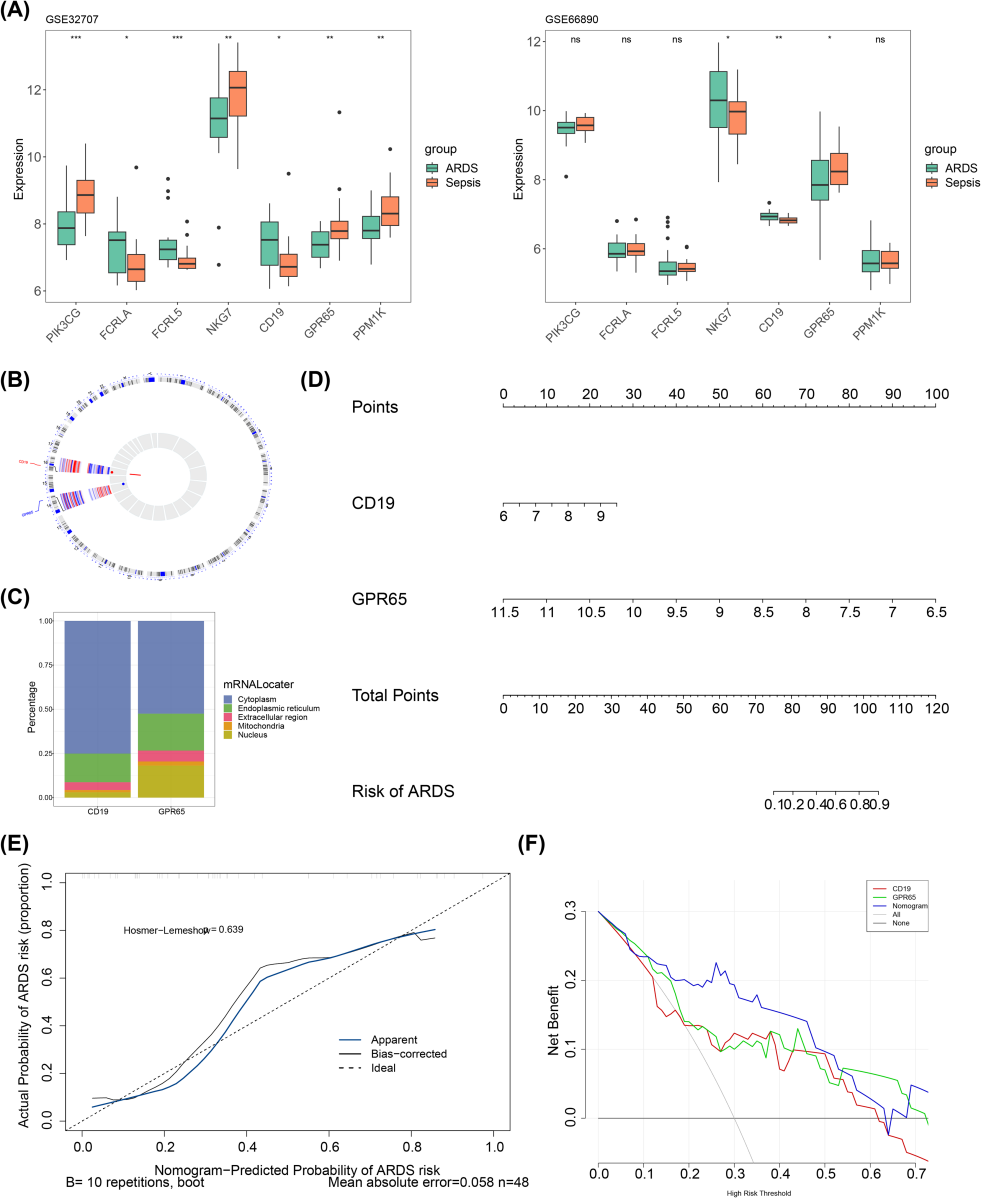
The key genes with different distribution exhibited excellent predictive ability for sepsis-induced ARDS. *, p < 0.05; **, p < 0.01; ***, p < 0.001, ns, not significant; **(A)** Training set key genes expression and validation set key genes expression; **(B)** Chromosomal localization map of key genes; **(C)** Subcellular localization prediction map; **(D)** Nomogram; **(E)** Calibration curve; **(F)** DCA curve.

The expression levels of these seven candidate key genes were assessed between the ARDS and sepsis groups in both GSE32707 and GSE66890 datasets. The results revealed that the expression trends of CD19 and GPR65 were consistent in both datasets, with CD19 being significantly upregulated in the SRDS group, while GPR65 was significantly downregulated (*P* < 0.05) (**Figure 3A**). Thus, CD19 and GPR65 were recorded as key genes associated with sialylation in sepsis-induced ARDS. Subsequently, the distribution of key genes was explored. Chromosomal localization results revealed that CD19 was located on chromosome 16, and GPR65 was situated on chromosome 14 (**Figure 3B**). Meanwhile, subcellular localization analysis demonstrated predominant cytoplasmic expression for both CD19 and GPR65 (**Figure 3C**). After that, the predictive ability of key genes as a whole for sepsis-induced ARDS was evaluated. A nomogram model was created based on CD19 and GPR65. Within this model, a higher total point demonstrated an increased probability of sepsis-induced ARDS (**Figure 3D**). The calibration curve demonstrated a close resemblance between the slope of the nomogram model and the ideal curve (*P* = 0.639), further emphasizing its predictive accuracy (**Figure 3E**). Additionally, the nomogram model exhibited a greater net benefit compared to a single key gene in DCA, highlighting its superior performance (**Figure 3F**).

### 3.4 Specific signaling mechanisms of CD19 and GPR65

**Figure 4:**
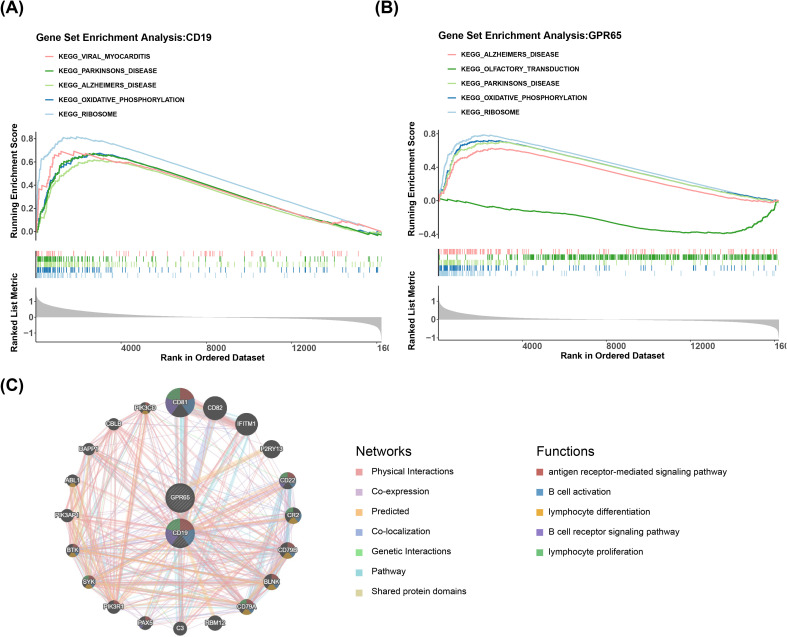
Specific signaling mechanisms of CD19 and GPR65. **(A)** KEGG enrichment analysis of CD19-associated pathways. **(B)** KEGG enrichment analysis of GPR65-related pathways. **(C)** Co-expression network mapping of coregulated genes.

GSEA was implemented to probe the specific signaling mechanisms of CD19 and GPR65 in sepsis-induced ARDS. The results demonstrated that among the significantly enriched top 5 pathways, high expression of CD19 and GPR65 was markedly co-enriched in oxidative phosphorylation, ribosome, Alzheimer’s disease, and Parkinson’s disease, whereas low expression of GPR65 was significantly enriched in olfactory transduction (*P*.adjust < 0.05) (**Figures 4A, B**). In addition, CD19 and GPR65 were significantly co-enriched in apoptosis, B-cell receptor signaling pathway, NOD-like receptor signaling pathway and others (**Supplementary Table 2**). Additionally, top 20 genes associated with CD19 and GPR65 functions were predicted in the GeneMANIA database, such as CD81, CD22, CD79A, SYK, etc. Their common functions included B cell activation, lymphocyte differentiation, B cell receptor signaling pathway, etc (**Figure 4C**).

### 3.5 Multiple factors and drugs existed to modulate relationships with key genes

**Figure 5:**
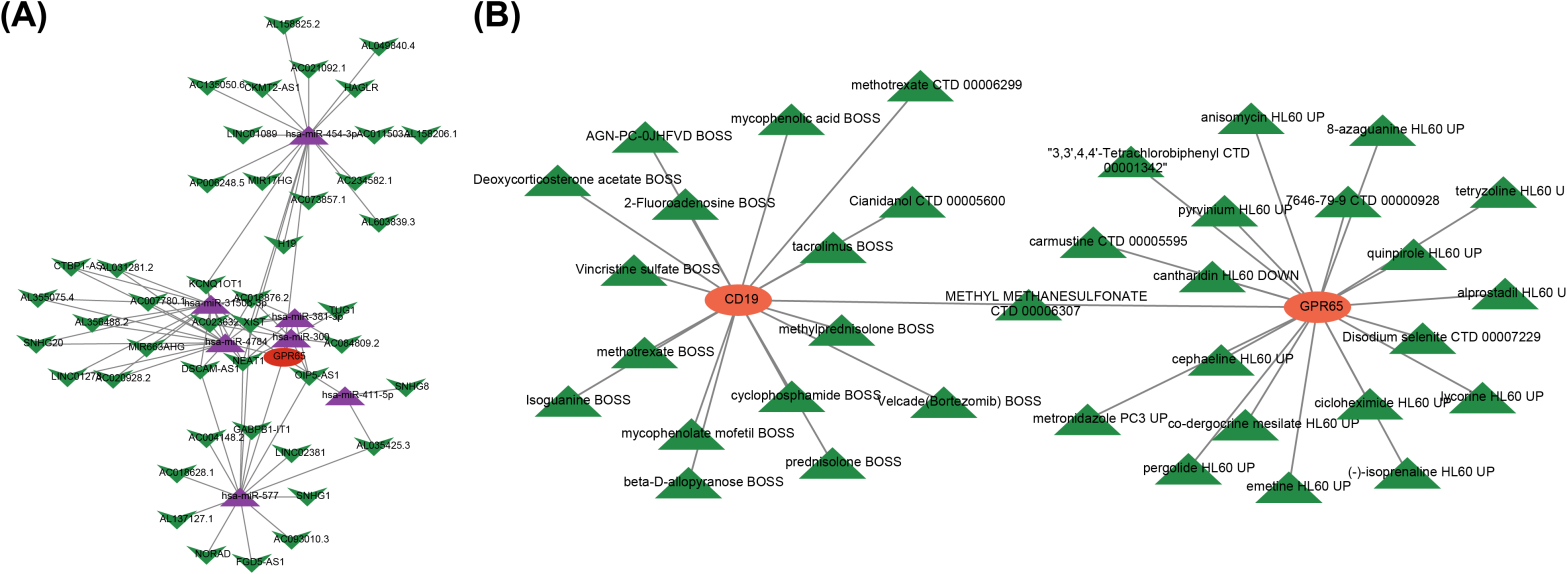
Multiple factors and drugs existed to modulate relationships with key genes. **(A)** Core ceRNA network diagram; **(B)** Key gene-drug relationship.

A lncRNA-miRNA-mRNA network containing 52 nodes and 85 edges was constructed by applying multiple databases for prediction. In this network, GPR65 expression was regulated by several factors, such as OIP5-AS1 regulated the expression of GPR65 through hsa-miR-300, hsa-miR-381-3p, hsa-miR-3150b-3p, hsa-miR-411-5p, and hsa-miR-577 (**Figure 5A**). Meanwhile, several other lncRNAs, including NEAT1, TUG1, H19, and SNHG1, exerted regulatory functions in the modulation of GPR65 expression. Subsequently, 36 drugs were found to target key genes. Among them, methyl methanesulfonate had target relationship with both CD19 and GPR65 (**Figure 5B**). Besides, some other important drugs were predicted, including alprostadil, tacrolimus, methotrexate, etc.

### 3.6 Nine cell types were annotated by scRNA-seq data analysis

**Figure 6:**
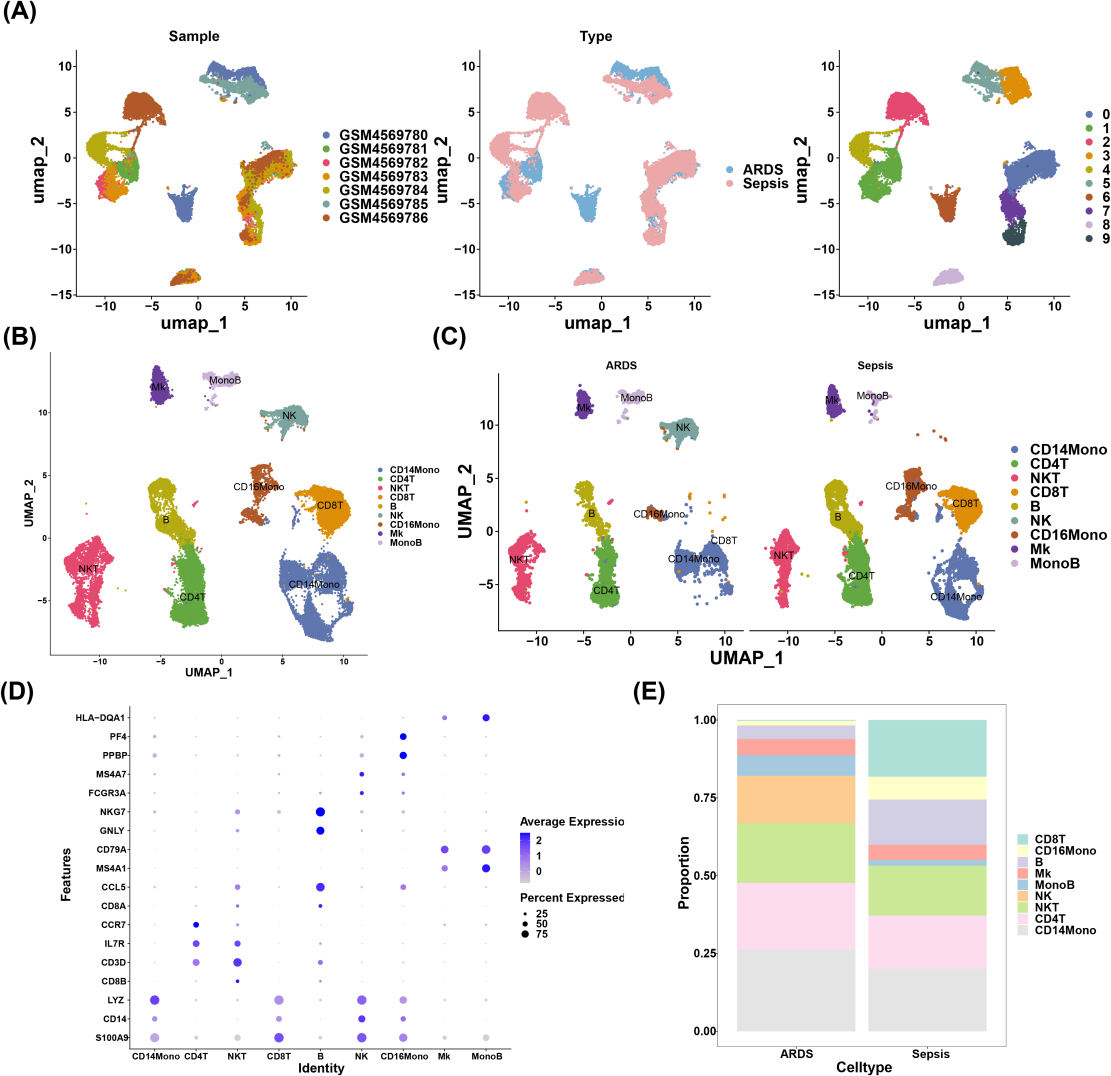
Nine cell types were annotated by scRNA-seq data analysis. **(A)** UMAP dimensionality reduction clustering result plot; **(B)** Annotation of cell subpopulations; **(C)** The result of cell subpopulation distribution in two groups; **(D)** The expression of marker genes for these nine cell types was visualized by bubble plots; **(E)** Graph of cell proportions in different groups.

There were 24,796 cells and 16,788 genes in the scRNA-seq data before quality control (QC) (**Supplementary Figure 1A**). Then, 20,290 cells and 14,008 genes were retained after quality control in the GSE151263 dataset (**Supplementary Figure 1B**). A total of 2,000 highly variable genes were identified, followed by labeling the top 10 highly variable genes (**Supplementary Figure 1C**). PCA results demonstrated a largely centralized distribution of ARDS and sepsis genes, with no significant outliers (**Supplementary Figure 1D**). Following this, the top 40 PCs were selected for subsequent analysis (**Supplementary Figure 1E**). After the reduced dimensional clustering analysis, 10 cellular taxa were obtained (**Figure 6A**). Furthermore, 9 cell types were identified through annotation, which contained CD14 monocytes (CD14Mono) (LYZ, CD14, and S100A9), CD4^+^ T (CD4T) cell (CCR7), natural killer T (NKT) cell (IL7R and CD3D), CD8T cell (CD8A and CD8B), B cell (CD79A and MS4A1), natural killer (NK) cell (NKG7 and GNLY), CD16 monocytes (CD16Mono) (MSA7 and FCGR3A), megakaryocyte (Mk) (PF4 and PPBP), monocytes B (MonoB) cell (HLA-DQA1) (**Figure 6B**). Of these, nine cell types were found in the ARDS group, while eight cell types were found in the sepsis group (**Figure 6C**). The expression of marker genes for these nine cell types was visualized by bubble plots (**Figure 6D**). Also, we found that there were significant changes in the proportion of NK, CD14Mono, and CD8T between ARDS and sepsis groups (**Figure 6E**).

### 3.7 The key cells that communicated with other cells had different stages of differentiation

**Figure 7:**
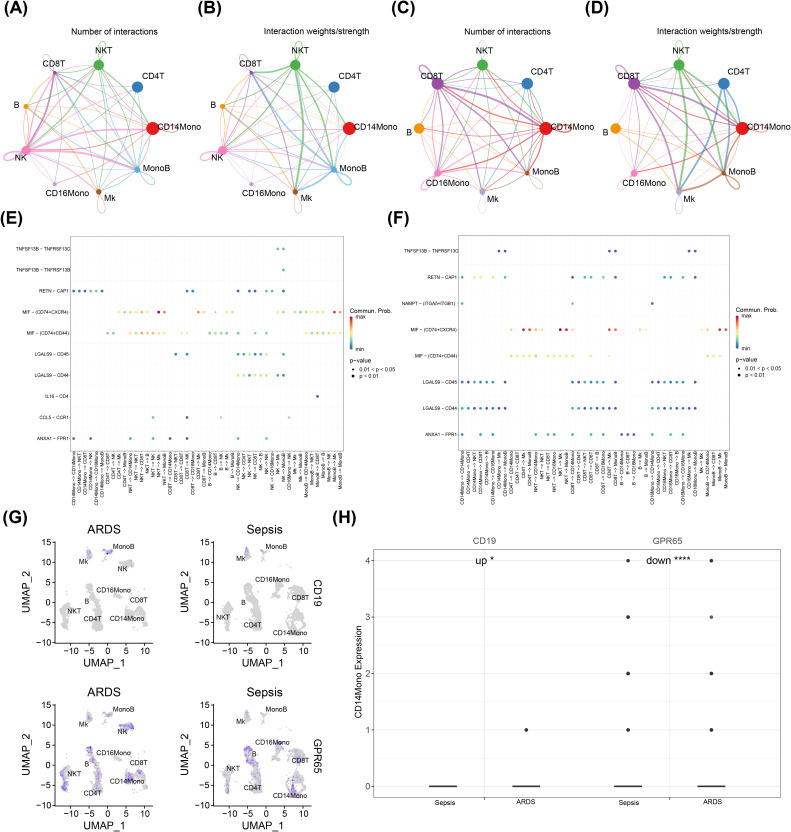
Communication between key cells and other cells and expression of key genes in different cells. *, p < 0.05; ****, p < 0.0001; **(A, B)** Chord diagram of differences in the number and intensity of cell-cell communication interactions among ARDS cell subsets; **(C, D)** Chord diagram depicting differences in the number and intensity of cell-cell communication interactions among sepsis cell subsets; **(E)** Bubble chart of ARDS cell communication; **(F)** Bubble chart of sepsis cell communication; **(G)** Expression of genes in different cells; **(H)** The expression levels of both CD19 and GPR65 were significantly different in CD14Mono between two groups.

Cell communication analysis was implemented to probe the exchange of information between the nine cell types obtained by annotation. In both the ARDS (**Figures 7A, B**) and sepsis groups (**Figures 7C, D**), there was an increased number and intensity of interactions between CD14Mono and other cell types. Additionally, our findings demonstrated that the likelihood of cellular communication between NKT and Mk via MIF - (CD74+CXCR4) was significantly higher in the ARDS group compared to other groups (**Figure 7E**). Conversely, in the sepsis group, the probability of cellular communication between NKT and CD16Mono via MIF - (CD74+CXCR4) exhibited the highest magnitude (**Figure 7F**). Next, the expression levels of key genes were evaluated in cells obtained by annotation. The results demonstrated that CD19 was highly expressed in Mk and MonoB, while GPR65 was more widely expressed in almost all cell types (**Figure 7G**). Afterwards, the expression of key genes was compared in these cell types between the ARDS and sepsis groups. The results indicated that the expression levels of both CD19 and GPR65 were significantly different in CD14Mono between two groups (**Figure 7H**, **Supplementary Figure 2**). Therefore, CD14Mono was selected as the key cell.

**Figure 8:**
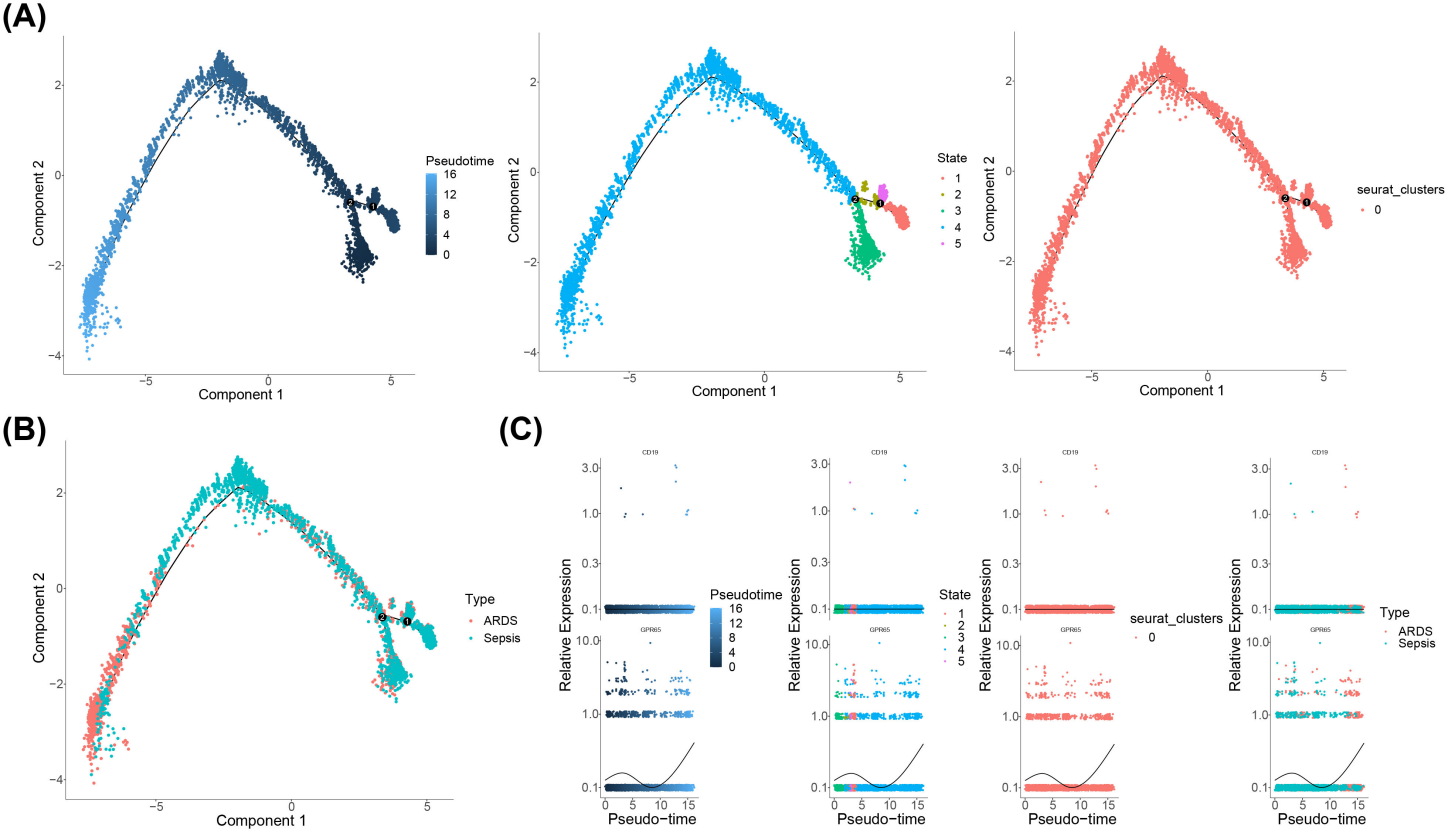
Pseudo-time analysis of CD14Mono. **(A)** Temporal differences in cell differentiation, stages of cell differentiation and cell cluster; **(B)** Stages of cell cluster differentiation; **(C)** The expression levels of CD19 and GPR65 were evaluated at various stages of differentiation in CD14Mono.

The pseudo-time analysis was conducted for key cells. The findings revealed a temporal differentiation of CD14Mono from right to left, exhibiting five distinct states with state three being the predominant state throughout the observation period (**Figure 8A**). Furthermore, our results demonstrated a gradual differentiation of CD14Mono from the sepsis group towards sepsis-induced ARDS (**Figure 8B**). In addition, the expression levels of CD19 and GPR65 were evaluated at various stages of differentiation in CD14Mono. The findings demonstrated that CD19 expression remained relatively stable throughout the entire differentiation process of CD14Mono, whereas GPR65 expression exhibited a pattern characterized by an initial increase, followed by a decrease, and then another subsequent increase (**Figure 8C**).

### 3.8 Expression evaluation of key genes

**Figure 9:**
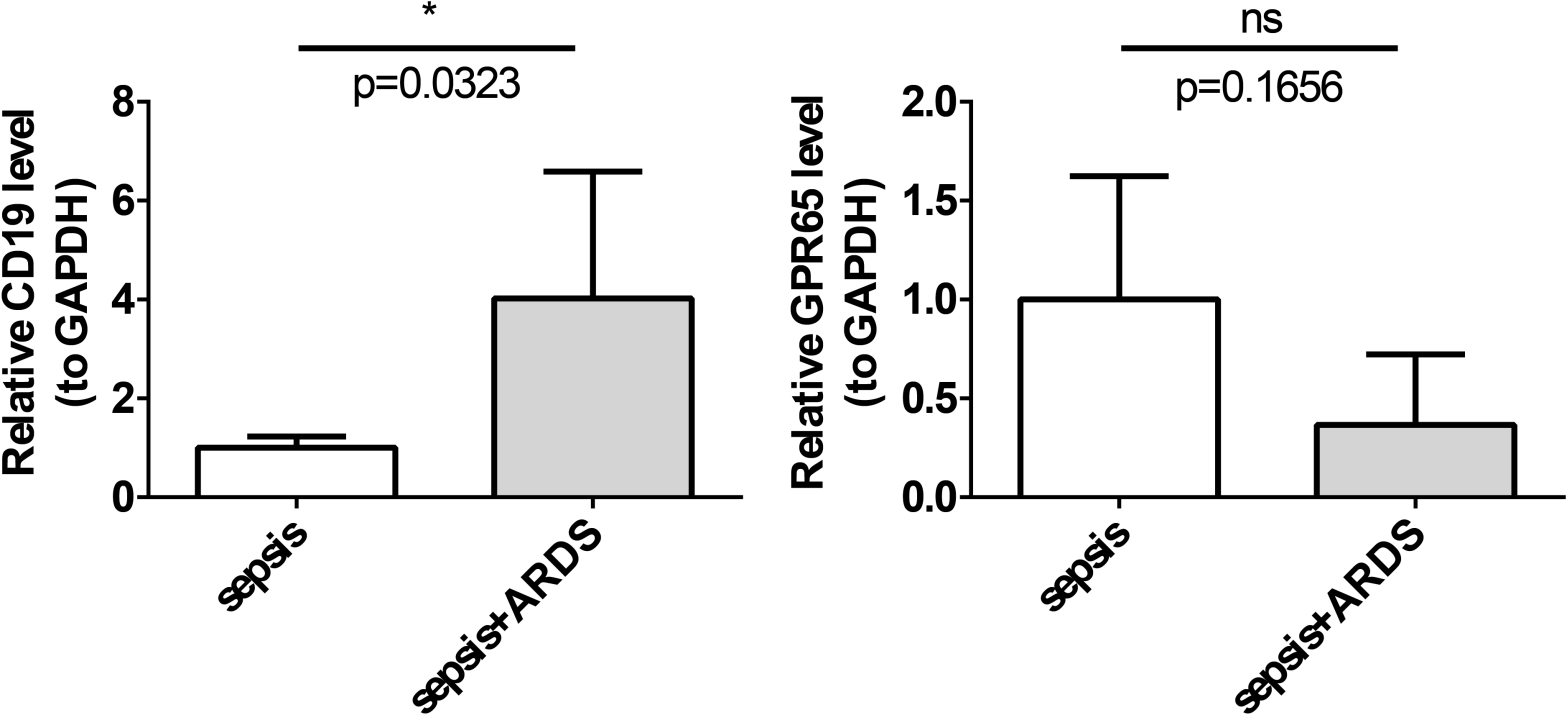
Expression evaluation of key genes. *, p < 0.05, ns, not significant; **(A)** The expression level of CD19 was significantly higher in the ARDS group; **(B)** The expression level of GPR65 was higher in the sepsis group.

The expression levels of the key genes in the ARDS and sepsis groups were assessed using RT-qPCR. The results demonstrated that the expression level of CD19 remained consistent with the public database, and its expression was significantly higher in the ARDS group, suggesting that the data from the public databases were reliable and that CD19 might serve as a potential biomarker. The expression trend of GPR65 remained consistent with the public database, yet it did not exhibit statistically significant differences in the sepsis and ARDS groups (*P* < 0.05) (**Figure 9**). This may be due to the small sample size in the PCR validation. These findings suggest that the role of GPR65 in sepsis and ARDS is complex, and further experimental validation and mechanistic studies are required.

## 4 Discussion

Acute Respiratory Distress Syndrome (ARDS) is a life-threatening condition characterized by heterogeneous etiologies (33). Among these, sepsis is the predominant cause, accounting for 32% of ARDS cases. Sialylation, a critical post-translational modification, significantly influences immune cell function and inflammatory responses (34). In ARDS, aberrant sialylation affects CD14 monocytes, which are key immune cells that express sialylated receptors. Dysregulated sialylation impairs the function of these monocytes, impacting their migration and ability to phagocytose pathogens. This dysfunction contributes to excessive inflammation and tissue damage. The underlying mechanism involves interactions between sialylated receptors and endothelial selectins. Emerging studies suggest that the sialylation of C1 esterase inhibitor may play a significant role in ARDS (35, 36). Additionally, sialylation can modulate the activity of cytokines and chemokines, thereby influencing the inflammatory cascade in ARDS. However, the specific role of sialylation in sepsis-induced ARDS remains to be fully elucidated. Therefore, investigating the potential biological functions of sialylation-related genes (SRGs) in sepsis-induced ARDS could provide deeper insights into its pathogenesis and offer valuable guidance for the diagnosis and treatment of patients with sepsis-induced ARDS.

In this study, we combined single-cell sequencing and transcriptome analysis to investigate the mechanisms of sialylation-related genes in sepsis-induced ARDS. We identified CD19 and GPR65 as key genes associated with sialylation in this context. The nomogram model we constructed demonstrated that CD19 and GPR65, when considered jointly, exhibited strong predictive power for sepsis-induced ARDS. Additionally, we found that CD19 and GPR65 were significantly enriched in pathways related to apoptosis and B-cell receptor signaling. Furthermore, we identified several important regulators and potential drug targets, including NEAT1, OIP5-AS1, alprostadil, and tacrolimus. Our scRNA-seq data analysis revealed nine distinct cell types, with CD14Mono emerging as the key cell type. CD14Mono exhibited extensive and intense communication with other cells and displayed various stages of differentiation. Notably, GPR65 expression underwent dynamic changes during the differentiation process of CD14Mono.

Our preliminary research indicates that CD19 and GPR65 are key sialylation-related genes in sepsis-induced ARDS, with CD19 upregulated and GPR65 downregulated in ARDS patients. CD19, a B-cell surface antigen and member of the immunoglobulin superfamily, is vital for B-cell development, proliferation, differentiation, and signaling (37–41). However, B cells can contribute to cytokine storms in severe infections, driving ARDS development (34, 42–45). Thus, CD19 may impact ARDS through its role in B cells. GPR65 (TDAG8), a proton-sensing G protein-coupled receptor, is involved in various biological functions. It has been shown to protect against LPS-induced acute lung injury and may influence ARDS pathology by modulating inflammatory mediator production and release, including cytokines like IL-6 (46). Furthermore, GPR65 may be involved in the pathological processes of ARDS by influencing the production and release of inflammatory mediators (47–50). Our study found significant upregulation of CD19 and downregulation of GPR65 in sepsis-induced ARDS patients, suggesting they could be new therapeutic targets for this condition.

This study shows that high expression of CD19 and GPR65 is significantly associated with pathways related to oxidative phosphorylation, ribosome function, apoptosis, B-cell receptor signaling, and NOD-like receptor signaling. These pathways are key in ARDS pathophysiology. Oxidative phosphorylation is modulated by mechanisms like MSC-EVs, ketone body metabolism, and S1PR3 inhibition (51). Ribosome-related genes are differentially expressed in sepsis-induced ARDS. Dysregulated apoptosis can worsen lung injury. Dysregulation of apoptotic processes can lead to excessive cell death, potentially exacerbating lung injury and impairing recovery from ARDS (52–55). BAP31 deficiency may improve ALI and ARDS by reducing neutrophil recruitment via the NF-κB pathway (56). NOD-like receptor signaling is involved in pathogen recognition and immune response modulation in ARDS. These findings suggest that sialylation plays a crucial role in sepsis-induced ARDS. However, previous phenotype classifications based on clinical markers were limited. Phenotype classification based on key gene functional analysis is necessary for precise treatment of sepsis-induced ARDS.

We also assessed the regulatory networks and drug predictions for sialylation-related genes (CD19 and GPR65). A lncRNA-miRNA-mRNA network with 52 nodes and 85 edges was constructed using multiple databases. Key lncRNAs regulating GPR65 expression include OIP5-AS1, NEAT1, TUG1, H19, and SNHG1. OIP5-AS1 worsens LPS-induced ALI/ARDS via the miR-223/NLRP3 axis (57), TUG1 reverses LPS-induced apoptosis and inflammation in macrophages (58, 59), and NEAT1 is linked to the inflammatory response in ARDS (60–63). Additionally, drugs like alprostadil, tacrolimus, and methotrexate were identified as targeting these key genes. Alprostadil protects against ARDS by inhibiting apoptosis and suppressing MAPK and NF-κB pathways (64–66), while tacrolimus can reverse ARDS (67). Further research on these lncRNAs and drugs is crucial for understanding the pathogenesis and developing treatments for sepsis-induced ARDS.

Single-cell RNA sequencing (scRNA-seq) has deepened our understanding of cellular heterogeneity and dynamics in ARDS. Our study identified nine distinct cell types, with CD14+ monocytes (CD14Mono) emerging as a key population. This aligns with recent scRNA-seq literature highlighting the critical role of CD14+ monocytes in ARDS pathogenesis. For instance, spatial transcriptomics has mapped immune-stromal interactions in lung niches, revealing immunosuppressive myeloid subsets that may parallel CD14Mono-mediated immune dysregulation (68). CD14-dependent pathways, potentially related to LPS, LBP, and sCD14 concentrations, have been implicated in pneumonia-related inflammation in ARDS. Changes in CD14+ monocytes may correlate with treatment outcomes in ARDS immunomodulatory therapies (69). Our computational analysis suggests novel interactions between CD14+ monocytes and other immune subsets, particularly through CD19 and GPR65. The identification of CD19 and GPR65 as modulators of monocyte behavior opens new avenues for precision immunology. Future studies should leverage AI-driven frameworks like iMLGAM to predict patient-specific responses to such interventions (10). Integrating these insights with spatial multi-omics and AI-driven analytics will be crucial for advancing ARDS therapeutics.

## 5 Conclusions

This study identified CD19 and GPR65 as key sialylation-related genes in sepsis-induced ARDS through bioinformatics analyses. A nomogram model was built to assess their predictive power for ARDS. Enrichment analysis, molecular regulatory network construction, and drug prediction were performed to explore their mechanisms. Single-cell sequencing revealed significant differences in CD19 and GPR65 expression in CD14Mono cells between ARDS and sepsis groups, with GPR65 showing an initial increase, then decrease, and a subsequent increase during differentiation. These findings offer new insights into ARDS diagnosis and treatment via sialylation, highlighting our ongoing commitment to monitor these mechanisms’ effects.

## Data Availability

The datasets analysed during the current study are available in the GEO: GSE32707 and GSE66890 and GSE151263 repository, [http://www.ncbi.nlm.nih.gov/geo/]; and MSigDB repository, [https://www.gsea-msigdb.org/ gsea/msigdb].

## Supplementary material

Supplementary Figure 1
**(A)** Violin plot before quality control; **(B)** Violin plot after quality control; **(C)** A total of 2,000 highly variable genes were identified, followed by labeling the top 10 highly variable genes; **(D)** Scatter plot of principal components for dimensionality reduction and clustering of single-cell sequencing data; **(E)** Scree plot of principal components for dimensionality reduction and clustering of single-cell sequencing data.

Supplementary Figure 2
**(A-H)** The expression levels of both CD19 and GPR65 in 8 cell types between two groups.

Supplementary Table 1 The primer sequences for PCR.

Supplementary Table 2 CD19 and GPR65 were significantly co-enriched pathways.

## References

[1] Bos LDJ Ware LB. Acute respiratory distress syndrome: causes, pathophysiology, and phenotypes. Lancet. (2022) 400:1145–56. doi: 10.1016/S0140-6736(22)01485-4 36070787

[2] Thompson BT Chambers RC Liu KD. Acute respiratory distress syndrome. N Engl J Med. (2017) 377:562–72. doi: 10.1056/NEJMra1608077 28792873

[3] Gorman EA O’Kane CM McAuley DF. Acute respiratory distress syndrome in adults: diagnosis, outcomes, long-term sequelae, and management. Lancet. (2022) 400:1157–70. doi: 10.1016/S0140-6736(22)01439-8 36070788

[4] Sinha P Meyer NJ Calfee CS. Biological phenotyping in sepsis and acute respiratory distress syndrome. Annu Rev Med. (2023) 74:457–71. doi: 10.1146/annurev-med-043021-014005 PMC10617629 36469902

[5] Xu H Sheng S Luo W Xu X Zhang Z. Acute respiratory distress syndrome heterogeneity and the septic ARDS subgroup. Front Immunol. (2023) 14:1277161. doi: 10.3389/fimmu.2023.1277161 38035100 PMC10682474

[6] Zhou M Lv S Hou Y Zhang R Wang W Yan Z. Characterization of sialylation-related long noncoding RNAs to develop a novel signature for predicting prognosis, immune landscape, and chemotherapy response in colorectal cancer. Front Immunol. (2022) 13:994874. doi: 10.3389/fimmu.2022.994874 36330513 PMC9623420

[7] Bongiovanni A Cusimano A Annunziata I d’Azzo A. Sialylation of host proteins as targetable risk factor for COVID-19 susceptibility and spreading: A hypothesis. FASEB Bioadv. (2021) 3:192–7. doi: 10.1096/fba.2020-00073 PMC7944874 33733058

[8] Wygrecka M Kosanovic D Wujak L Reppe K Henneke I Frey H. Antihistone properties of C1 esterase inhibitor protect against lung injury. Am J Respir Crit Care Med. (2017) 196:186–99. doi: 10.1164/rccm.201604-0712OC 28005404

[9] Chen H Chen X Ding J Xue H Tang X Li X. Single nuclear RNA sequencing and analysis of basal cells in pulmonary acute respiratory distress syndrome. Gene. (2025) 936:149131. doi: 10.1016/j.gene.2024.149131 39622393

[10] Ye B Fan J Xue L Zhuang Y Luo P Jiang A. iMLGAM: Integrated Machine Learning and Genetic Algorithm-driven Multiomics analysis for pan-cancer immunotherapy response prediction. iMeta. (2025) e70011. doi: 10.1002/imt2.70011 40236779 PMC11995183

[11] Ye B Jiang A Liang F Wang C Liang X Zhang P. Navigating the immune landscape with plasma cells: A pan-cancer signature for precision immunotherapy. Biofactors. (2025) 51:e2142. doi: 10.1002/biof.v51.1 39495620

[12] Sun W Zhang P Ye B Situ MY Wang W Yu Y. Systemic immune-inflammation index predicts survival in patients with resected lung invasive mucinous adenocarcinoma. Transl Oncol. (2024) 40:101865. doi: 10.1016/j.tranon.2023.101865 38101174 PMC10727949

[13] Yuan K Zhao S Ye B Wang Q Liu Y Zhang P. A novel T-cell exhaustion-related feature can accurately predict the prognosis of OC patients. Front Pharmacol. (2023) 14:1192777. doi: 10.3389/fphar.2023.1192777 37284314 PMC10239809

[14] He D Yu Q Zeng X Feng J Yang R Wan H. Single-cell RNA sequencing and transcriptome analysis revealed the immune microenvironment and gene markers of acute respiratory distress syndrome. J Inflammation Res. (2023) 16:3205–17. doi: 10.2147/JIR.S419576 PMC10404049 37547124

[15] Ritchie ME Phipson B Wu D Hu Y Law CW Shi W. limma powers differential expression analyses for RNA-sequencing and microarray studies. Nucleic Acids Res. (2015) 43:e47. doi: 10.1093/nar/gkv007 25605792 PMC4402510

[16] Gustavsson EK Zhang D Reynolds RH Garcia-Ruiz S Ryten M. ggtranscript: an R package for the visualization and interpretation of transcript isoforms using ggplot2. Bioinformatics. (2022) 38:3844–6. doi: 10.1093/bioinformatics/btac409 PMC9344834 35751589

[17] Gu Z Eils R Schlesner M. Complex heatmaps reveal patterns and correlations in multidimensional genomic data. Bioinformatics. (2016) 32:2847–9. doi: 10.1093/bioinformatics/btw313 27207943

[18] Hänzelmann S Castelo R Guinney J. GSVA: gene set variation analysis for microarray and RNA-seq data. BMC Bioinf. (2013) 14:7. doi: 10.1186/1471-2105-14-7 PMC3618321 23323831

[19] Langfelder P Horvath S. WGCNA: an R package for weighted correlation network analysis. BMC Bioinf. (2008) 9:559. doi: 10.1186/1471-2105-9-559 PMC2631488 19114008

[20] Chen H Boutros PC. VennDiagram: a package for the generation of highly-customizable Venn and Euler diagrams in R. BMC Bioinf. (2011) 12:35. doi: 10.1186/1471-2105-12-35 PMC3041657 21269502

[21] Wu T Hu E Xu S Chen M Guo P Dai Z. clusterProfiler 4.0: A universal enrichment tool for interpreting omics data. . Innovation (Camb). (2021) 2:100141. doi: 10.1016/j.xinn.2021.100141 34557778 PMC8454663

[22] Sasikumar D Takano Y Zhao H Kohara R Hamada M Kobori Y. Caging and photo-triggered uncaging of singlet oxygen by excited state engineering of electron donor-acceptor-linked molecular sensors. Sci Rep. (2022) 12:11371. doi: 10.1038/s41598-022-15054-4 35790770 PMC9256616

[23] Twait EL Andaur Navarro CL Gudnason V Hu YH Launer LJ Geerlings MI. Dementia prediction in the general population using clinically accessible variables: a proof-of-concept study using machine learning. The AGES-Reykjavik study. BMC Med Inform Decis Mak. (2023) 23:168. doi: 10.1186/s12911-023-02244-x 37641038 PMC10463542

[24] Hou N Li M He L Xie B Wang L Zhang R. Predicting 30-days mortality for MIMIC-III patients with sepsis-3: a machine learning approach using XGboost. J Transl Med. (2020) 18:462. doi: 10.1186/s12967-020-02620-5 33287854 PMC7720497

[25] Zhang H Meltzer P Davis S. RCircos: an R package for Circos 2D track plots. BMC Bioinf. (2013) 14:244. doi: 10.1186/1471-2105-14-244 PMC3765848 23937229

[26] Xu J Yang T Wu F Chen T Wang A Hou S. A nomogram for predicting prognosis of patients with cervical cerclage. Heliyon. (2023) 9:e21147. doi: 10.1016/j.heliyon.2023.e21147 37885715 PMC10598483

[27] Shannon P Markiel A Ozier O Baliga NS Wang JT Ramage D. Cytoscape: a software environment for integrated models of biomolecular interaction networks. Genome Res. (2003) 13:2498–504. doi: 10.1101/gr.1239303 PMC403769 14597658

[28] Hao Y Hao S Andersen-Nissen E Mauck WM 3rd Zheng S Butler A. Integrated analysis of multimodal single-cell data. Cell. (2021) 184:3573–87.e29. doi: 10.1016/j.cell.2021.04.048 34062119 PMC8238499

[29] Zhang D Lu W Cui S Mei H Wu X Zhuo Z. Establishment of an ovarian cancer omentum metastasis-related prognostic model by integrated analysis of scRNA-seq and bulk RNA-seq. J Ovarian Res. (2022) 15:123. doi: 10.1186/s13048-022-01059-0 36424614 PMC9686070

[30] Jin S Guerrero-Juarez CF Zhang L Chang I Ramos R Kuan CH. Inference and analysis of cell-cell communication using CellChat. Nat Commun. (2021) 12:1088. doi: 10.1038/s41467-021-21246-9 33597522 PMC7889871

[31] Qiu X Mao Q Tang Y Wang L Chawla R Pliner HA. Reversed graph embedding resolves complex single-cell trajectories. Nat Methods. (2017) 14:979–82. doi: 10.1038/nmeth.4402 PMC5764547 28825705

[32] Shang Y Zhang Y Liu J Chen L Yang X Zhu Z. Decreased E2F2 expression correlates with poor prognosis and immune infiltrates in patients with colorectal cancer. J Cancer. (2022) 13:653–68. doi: 10.7150/jca.61415 PMC8771517 35069909

[33] Matthay MA Arabi YM Siegel ER Ware LB Bos LDJ Sinha P. Phenotypes and personalized medicine in the acute respiratory distress syndrome. Intensive Care Med. (2020) 46(12):2136–52. doi: 10.1007/s00134-020-06296-9 33206201 PMC7673253

[34] Gong R Luo H Long G Xu J Huang C Zhou X. Integrative proteomic profiling of lung tissues and blood in acute respiratory distress syndrome. Front Immunol. (2023) 14:1158951. doi: 10.3389/fimmu.2023.1158951 37197655 PMC10184823

[35] Xia F Chen H Liu Y Huang L Meng S Xu J. Development of genomic phenotype and immunophenotype of acute respiratory distress syndrome using autophagy and metabolism-related genes. Front Immunol. (2023) 14:1209959. doi: 10.3389/fimmu.2023.1209959 37936685 PMC10626539

[36] Sun M Yang Q Hu C Zhang H Xing L. Identification and validation of autophagy-related genes in sepsis-induced acute respiratory distress syndrome and immune infiltration. J Inflammation Res. (2022) 15:2199–212. doi: 10.2147/JIR.S355225 PMC8994633 35411170

[37] Fujimoto M Sato S. B cell signaling and autoimmune diseases: CD19/CD22 loop as a B cell signaling device to regulate the balance of autoimmunity. J Dermatol Sci. (2007) 46:1–9. doi: 10.1016/j.jdermsci.2006.12.004 17223015

[38] Lee DSW Rojas OL Gommerman JL. B cell depletion therapies in autoimmune disease: advances and mechanistic insights. Nat Rev Drug Discov. (2021) 20:179–99. doi: 10.1038/s41573-020-00092-2 PMC7737718 33324003

[39] Sermer D Elavalakanar P Abramson JS Palomba ML Salles G Arnason J. Targeting CD19 for diffuse large B cell lymphoma in the era of CARs: Other modes of transportation. Blood Rev. (2023) 57:101002. doi: 10.1016/j.blre.2022.101002 35989138

[40] Locke FL Filosto S Chou J Vardhanabhuti S Perbost R Dreger P. Impact of tumor microenvironment on efficacy of anti-CD19 CAR T cell therapy or chemotherapy and transplant in large B cell lymphoma. Nat Med. (2024) 30:507–18. doi: 10.1038/s41591-023-02754-1 PMC10878966 38233586

[41] Shi T Xu L Li X Huang L. The CD19(+) B cell as a marker for the febrile children infected with influenza A and Omicron variant. J Med Virol. (2023) 95:e29097. doi: 10.1002/jmv.v95.10 37828727

[42] Rossetti CL Cazarin J Hecht F Beltrão FEL Ferreira ACF Fortunato RS. COVID-19 and thyroid function: What do we know so far? Front Endocrinol (Lausanne). (2022) 13:1041676. doi: 10.3389/fendo.2022.1041676 36601011 PMC9806267

[43] Osto M Rehman R Ko A. A rare presentation of acute respiratory distress due to diffuse large B-cell lymphoma of the tongue base. Cureus. (2021) 13:e15124. doi: 10.7759/cureus.15124 34159026 PMC8213379

[44] Buchtele N Wohlfarth P Staudinger T Schellongowski P Traby L Vossen M. CD19 CAR T-cell infusion during severe COVID-19 acute respiratory distress syndrome in large B-cell lymphoma. Ann Hematol. (2023) 102:231–4. doi: 10.1007/s00277-022-05029-w PMC9672583 36399193

[45] Lu J Zeng X Lu W Feng J Yang Y Wei Y. Documenting the immune response in patients with COVID-19-induced acute respiratory distress syndrome. Front Cell Dev Biol. (2023) 11:1207960. doi: 10.3389/fcell.2023.1207960 37363730 PMC10288867

[46] Tsurumaki H Mogi C Aoki-Saito H Tobo M Kamide Y Yatomi M. Protective role of proton-sensing TDAG8 in lipopolysaccharide-induced acute lung injury. Int J Mol Sci. (2015) 16:28931–42. doi: 10.3390/ijms161226145 PMC4691092 26690120

[47] Zhang K Zhang MX Meng XX Zhu J Wang JJ He YF. Targeting GPR65 alleviates hepatic inflammation and fibrosis by suppressing the JNK and NF-κB pathways. Mil Med Res. (2023) 10:56. doi: 10.1186/s40779-023-00494-4 38001521 PMC10675918

[48] Lin R Wu W Chen H Gao H Wu X Li G. GPR65 promotes intestinal mucosal Th1 and Th17 cell differentiation and gut inflammation through downregulating NUAK2. Clin Transl Med. (2022) 12:e771. doi: 10.1002/ctm2.v12.3 35343079 PMC8958354

[49] Li G Lin J Gao X Su H Lin R Gao H. Intestinal epithelial pH-sensing receptor GPR65 maintains mucosal homeostasis via regulating antimicrobial defense and restrains gut inflammation in inflammatory bowel disease. Gut Microbes. (2023) 15:2257269. doi: 10.1080/19490976.2023.2257269 37749885 PMC10524779

[50] Marie MA Sanderlin EJ Satturwar S Hong H Lertpiriyapong K Donthi D. GPR65 (TDAG8) inhibits intestinal inflammation and colitis-associated colorectal cancer development in experimental mouse models. Biochim Biophys Acta Mol Basis Dis. (2022) 1868:166288. doi: 10.1016/j.bbadis.2021.166288 34628032 PMC8629932

[51] Sharma A Ahmad S Ahmad T Ali S Syed MA. Mitochondrial dynamics and mitophagy in lung disorders. Life Sci. (2021) 284:119876. doi: 10.1016/j.lfs.2021.119876 34389405

[52] He F Gu L Cai N Ni J Liu Y Zhang Q. The HMGB1-RAGE axis induces apoptosis in acute respiratory distress syndrome through PERK/eIF2α/ATF4-mediated endoplasmic reticulum stress. Inflammation Res. (2022) 71:1245–60. doi: 10.1007/s00011-022-01613-y 35871648

[53] Gouda MM Shaikh SB Bhandary YP. Inflammatory and fibrinolytic system in acute respiratory distress syndrome. Lung. (2018) 196:609–16. doi: 10.1007/s00408-018-0150-6 30121847

[54] Galani V Tatsaki E Bai M Kitsoulis P Lekka M Nakos G. The role of apoptosis in the pathophysiology of Acute Respiratory Distress Syndrome (ARDS): an up-to-date cell-specific review. Pathol Res Pract. (2010) 206:145–50. doi: 10.1016/j.prp.2009.12.002 20097014

[55] Chen J Ding W Zhang Z Li Q Wang M Feng J. Shenfu injection targets the PI3K-AKT pathway to regulate autophagy and apoptosis in acute respiratory distress syndrome caused by sepsis. Phytomedicine. (2024) 129:155627. doi: 10.1016/j.phymed.2024.155627 38696924

[56] Li GX Jiang XH Zang JN Zhu BZ Jia CC Niu KW. B-cell receptor associated protein 31 deficiency decreases the expression of adhesion molecule CD11b/CD18 and PSGL-1 in neutrophils to ameliorate acute lung injury. Int J Biochem Cell Biol. (2022) 152:106299. doi: 10.1016/j.biocel.2022.106299 36210579 PMC9484107

[57] Ji J Ye W Sun G. lncRNA OIP5-AS1 knockdown or miR-223 overexpression can alleviate LPS-induced ALI/ARDS by interfering with miR-223/NLRP3-mediated pyroptosis. J Gene Med. (2022) 24:e3385. doi: 10.1002/jgm.v24.4 34346534

[58] Hu L Ye H Liao J. LncRNA TUG1 reverses LPS-induced cell apoptosis and inflammation of macrophage via targeting MiR-221-3p/SPRED2 axis. Biosci Biotechnol Biochem. (2020) 84:2458–65. doi: 10.1080/09168451.2020.1806704 32841583

[59] Hu J Ge S Sun B Ren J Xie J Zhu G. Comprehensive analysis of potential ceRNA network and different degrees of immune cell infiltration in acute respiratory distress syndrome. Front Genet. (2022) 13:895629. doi: 10.3389/fgene.2022.895629 35719385 PMC9198558

[60] Ge S Hu J Gao S Ren J Zhu G. LncRNA NEAT1: A novel regulator associated with the inflammatory response in acute respiratory distress syndrome. Gene. (2023) 878:147582. doi: 10.1016/j.gene.2023.147582 37353041

[61] Yang Y Yang L Liu Z Wang Y Yang J. Long noncoding RNA NEAT 1 and its target microRNA-125a in sepsis: Correlation with acute respiratory distress syndrome risk, biochemical indexes, disease severity, and 28-day mortality. J Clin Lab Anal. (2020) 34:e23509. doi: 10.1002/jcla.v34.12 32785981 PMC7755762

[62] Zhou H Wang X Zhang B. Depression of lncRNA NEAT1 Antagonizes LPS-Evoked Acute Injury and Inflammatory Response in Alveolar Epithelial Cells via HMGB1-RAGE Signaling. Mediators Inflamm. (2020) 2020:8019467. doi: 10.1155/2020/8019467 32089649 PMC7025070

[63] Lv X Zhang XY Zhang Q Nie YJ Luo GH Fan X. lncRNA NEAT1 aggravates sepsis-induced lung injury by regulating the miR-27a/PTEN axis. Lab Invest. (2021) 101:1371–81. doi: 10.1038/s41374-021-00620-7 34239033

[64] Yan X Li Y Choi YH Wang C Piao Y Ye J. Protective effect and mechanism of alprostadil in acute respiratory distress syndrome induced by oleic acid in rats. Med Sci Monit. (2018) 24:7186–98. doi: 10.12659/MSM.909678 PMC6190919 30296789

[65] Adhikari N Burns KE Meade MO. Pharmacologic treatments for acute respiratory distress syndrome and acute lung injury: systematic review and meta-analysis. Treat Respir Med. (2004) 3:307–28. doi: 10.2165/00151829-200403050-00005 15606221

[66] Seo H Lopez CN Succar L Donahue KR. Evaluation of inhaled alprostadil in hospitalized adult patients. Ann Pharmacother. (2022) 56:671–8. doi: 10.1177/10600280211042675 34486414

[67] Guglielmi S Merz TM Gugger M Suter C Nicod LP. Acute respiratory distress syndrome secondary to antisynthetase syndrome is reversible with tacrolimus. Eur Respir J. (2008) 31:213–7. doi: 10.1183/09031936.00014707 18166599

[68] de Almeida Chuffa LG Freire PP Dos Santos Souza J de Mello MC de Oliveira Neto M Carvalho RF. Aging whole blood transcriptome reveals candidate genes for SARS-CoV-2-related vascular and immune alterations. J Mol Med (Berl). (2022) 100(2):285–301. 10.1007/s00109-021-02161-4 PMC8571664 34741638

[69] Martin TR Rubenfeld GD Ruzinski JT Goodman RB Steinberg KP Leturcq DJ. Relationship between soluble CD14, lipopolysaccharide binding protein, and the alveolar inflammatory response in patients with acute respiratory distress syndrome. Am J Respir Crit Care Med. (1997) 155:937–44. doi: 10.1164/ajrccm.155.3.9117029 9117029

