# Supplementary figures and images for: Integrating single-cell sequencing and transcriptome analysis to unravel the mechanistic role of sialylation-related genes in sepsis-induced acute respiratory distress syndrome
Source: Front Immunol. 2025 May 1;16:1528769. doi: 10.3389/fimmu.2025.1528769 (PMC12078151; doi:10.3389/fimmu.2025.1528769)

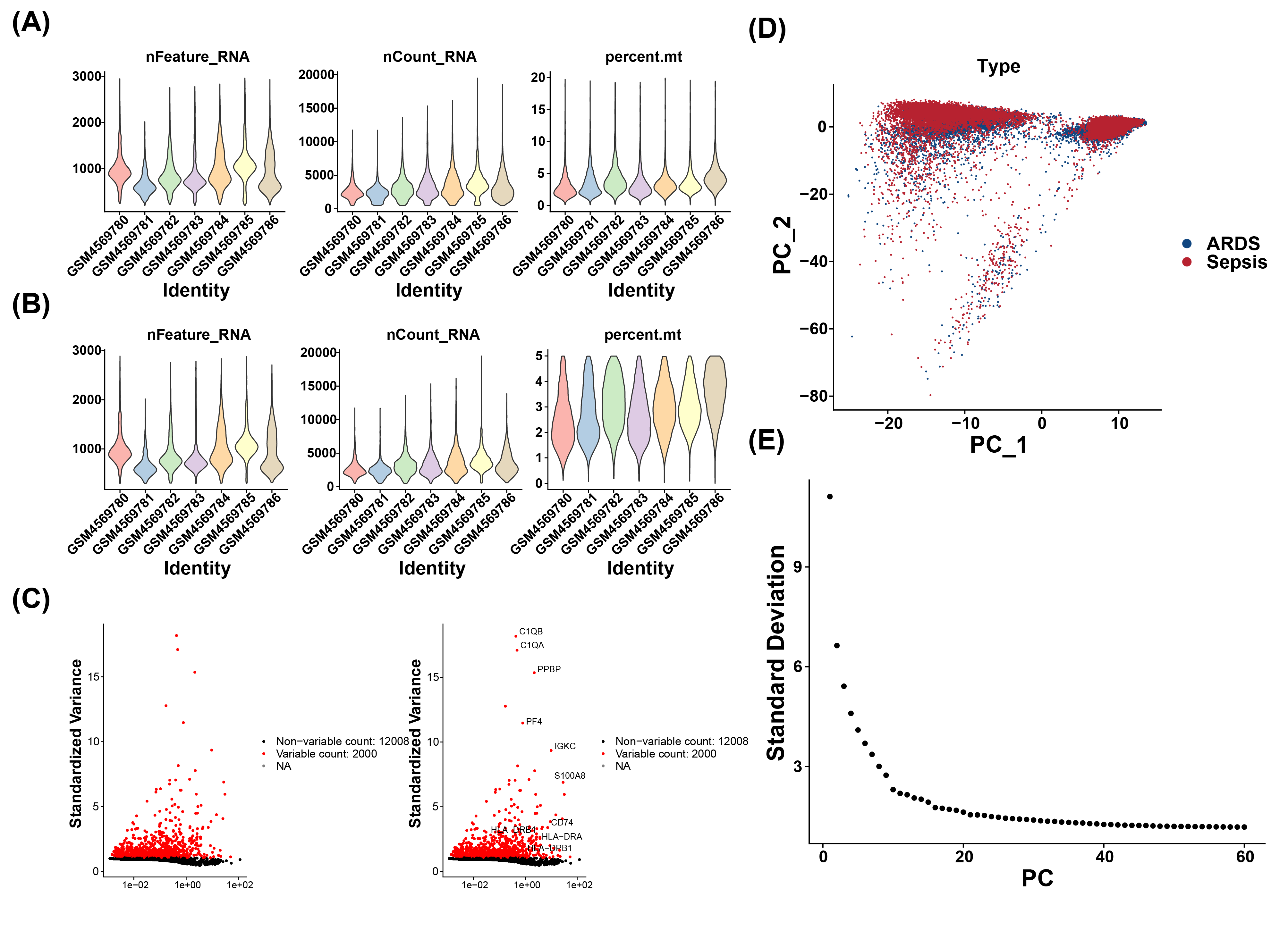

Supplement: Supplementary Figure 1 — (A) Violin plot before quality control; (B) Violin plot after quality control; (C) A total of 2,000 highly variable genes were identified, followed by labeling the top 10 highly variable genes; (D) Scatter plot of principal components for dimensionality reduction and clustering of single-cell sequencing data; (E) Scree plot of principal components for dimensionality reduction and clustering of single-cell sequencing data. [file Image1.tif]

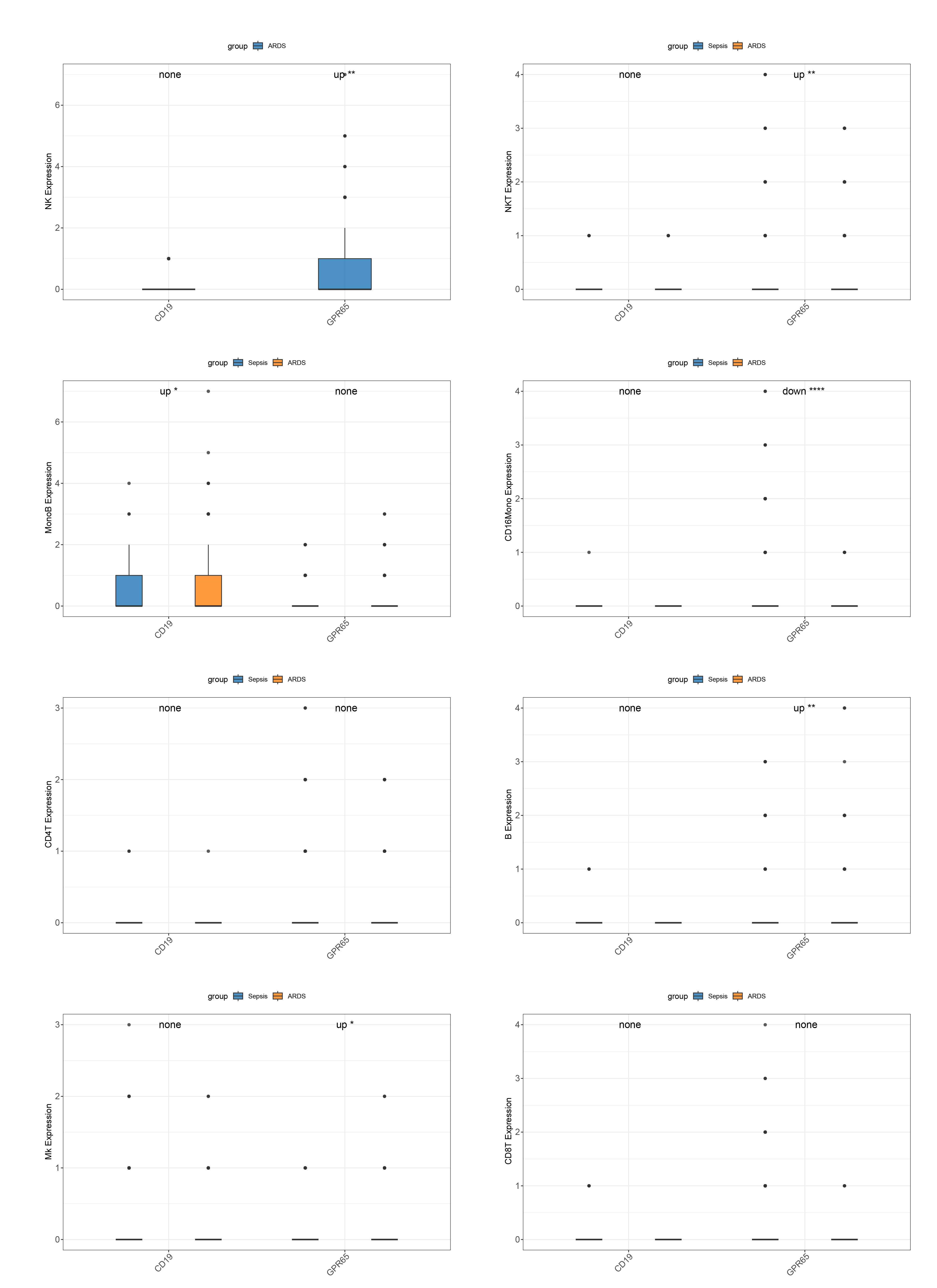

Supplement: Supplementary Figure 2 — (A-H) The expression levels of both CD19 and GPR65 in 8 cell types between two groups. [file Image2.tif]

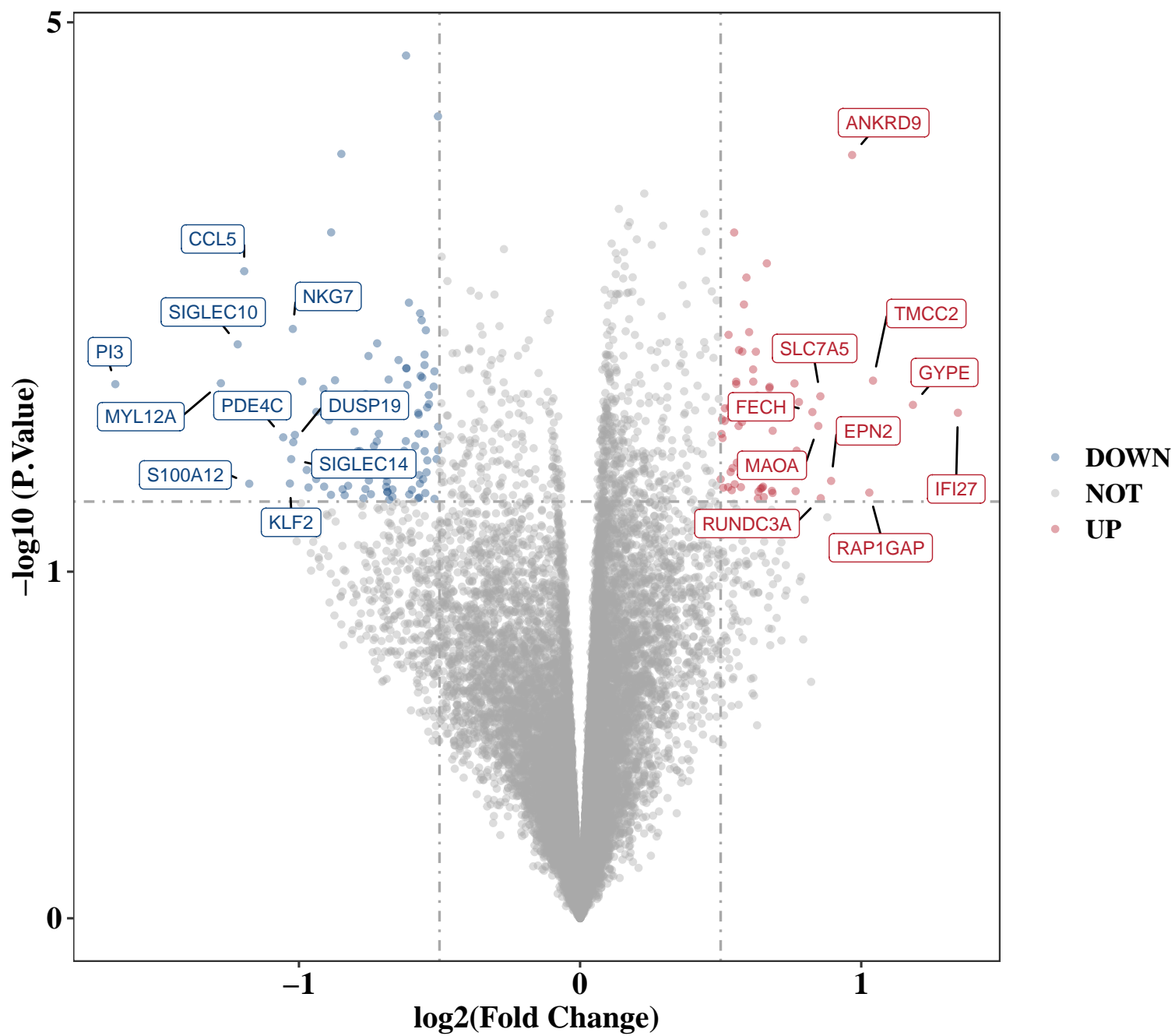

Supplement: Supplementary Table 1 — The primer sequences for PCR. [file DataSheet1.zip › Original data/01_DEGs/01.volcano(GSE32707).pdf]

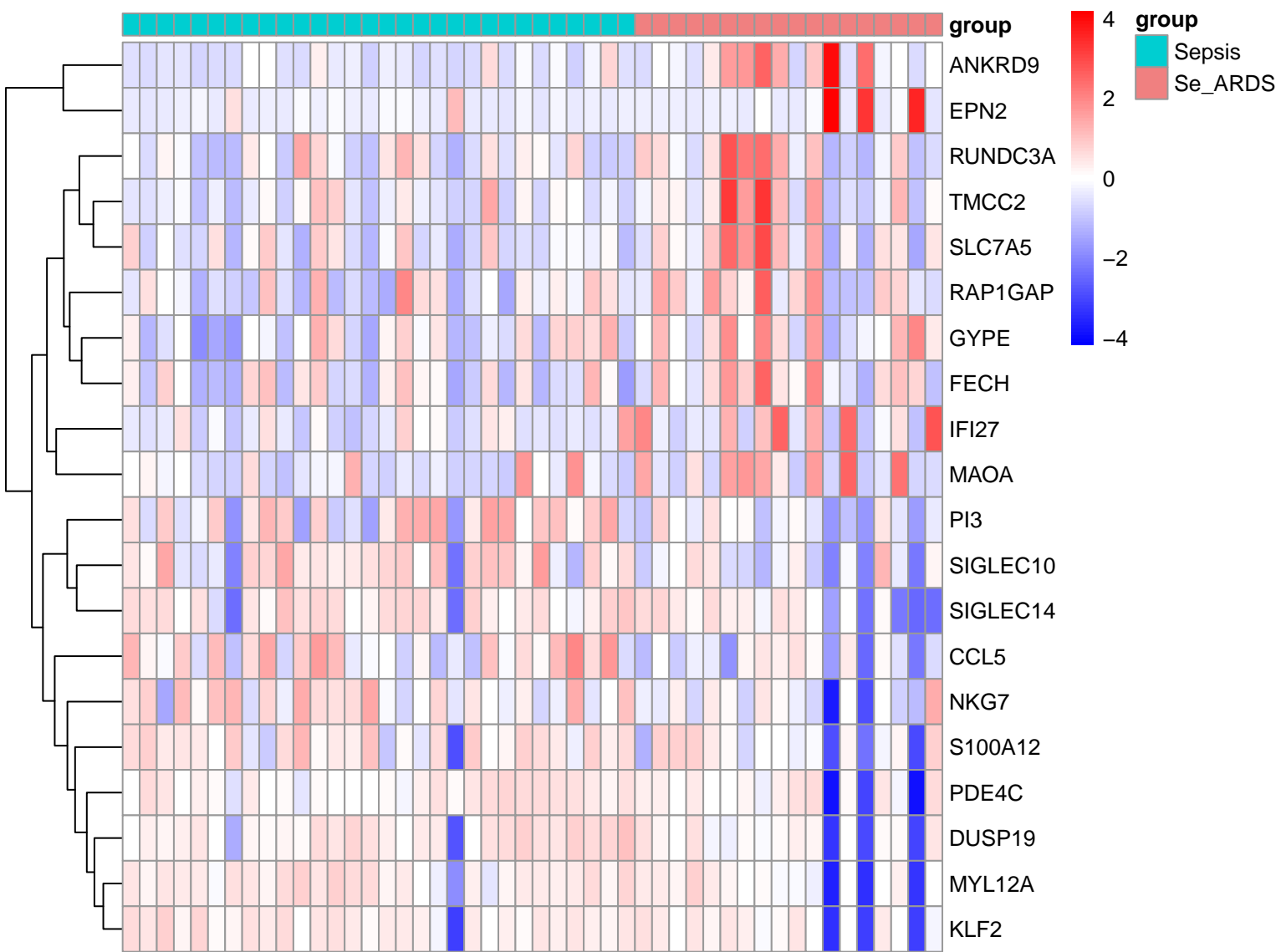

Supplement: Supplementary Table 1 — The primer sequences for PCR. [file DataSheet1.zip › Original data/01_DEGs/02.heatmap(GSE32707).pdf]

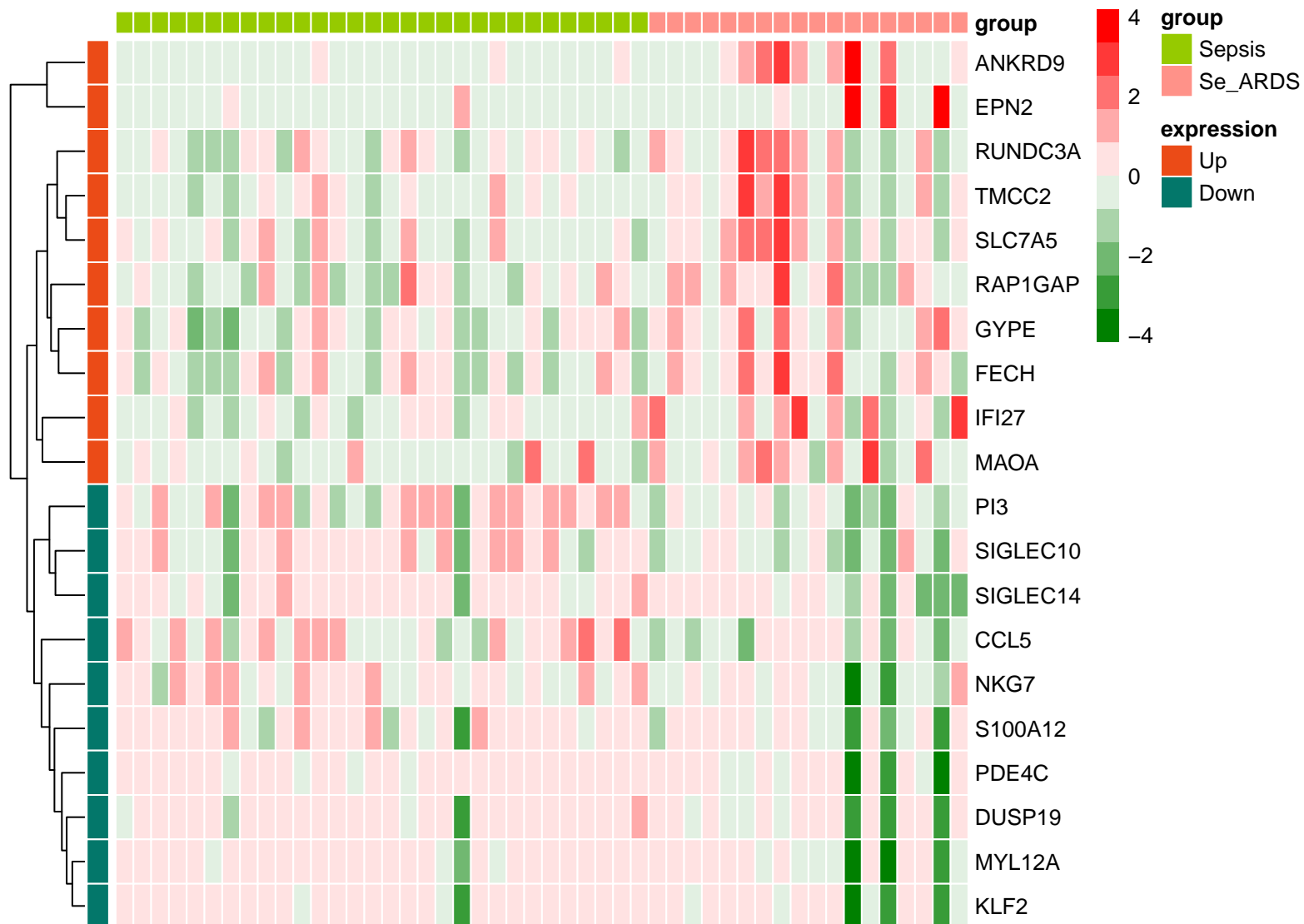

Supplement: Supplementary Table 1 — The primer sequences for PCR. [file DataSheet1.zip › Original data/01_DEGs/03.DEGs_heatmap(GSE32707).pdf]

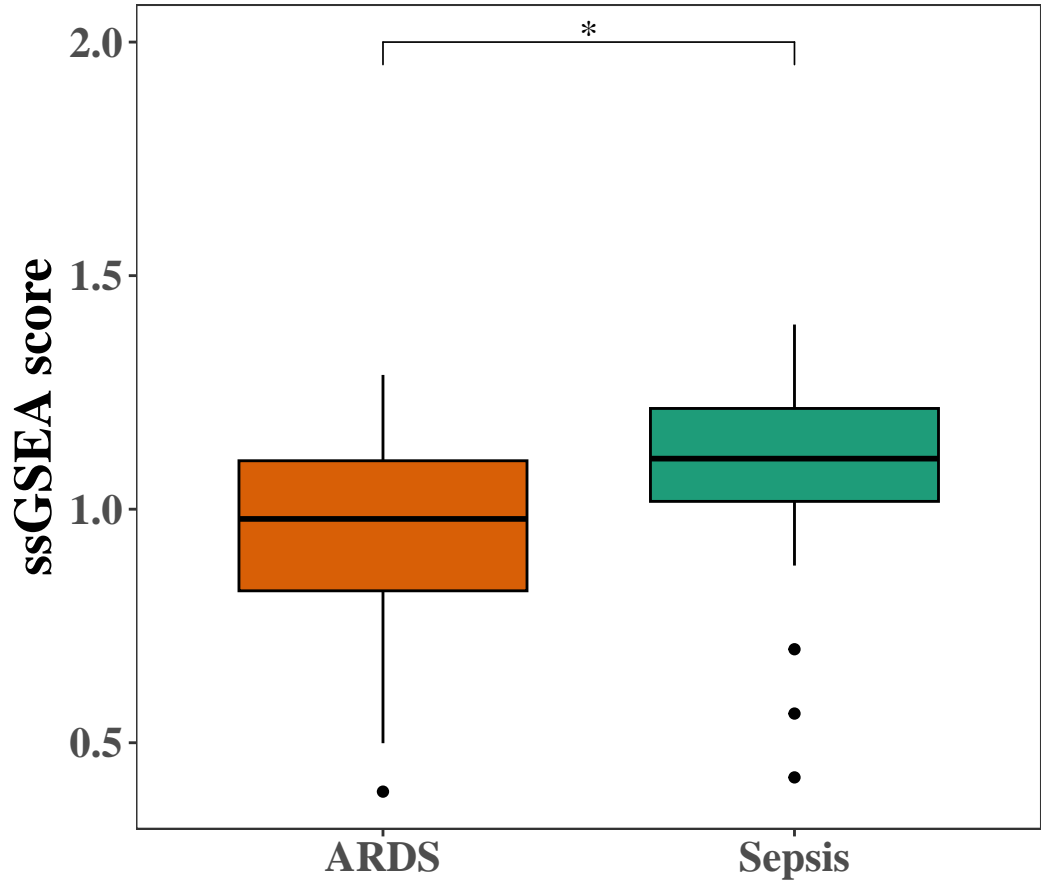

Supplement: Supplementary Table 1 — The primer sequences for PCR. [file DataSheet1.zip › Original data/02_WGCNA/03.ssgsea_score_boxplot.pdf]

# Sample Clustering

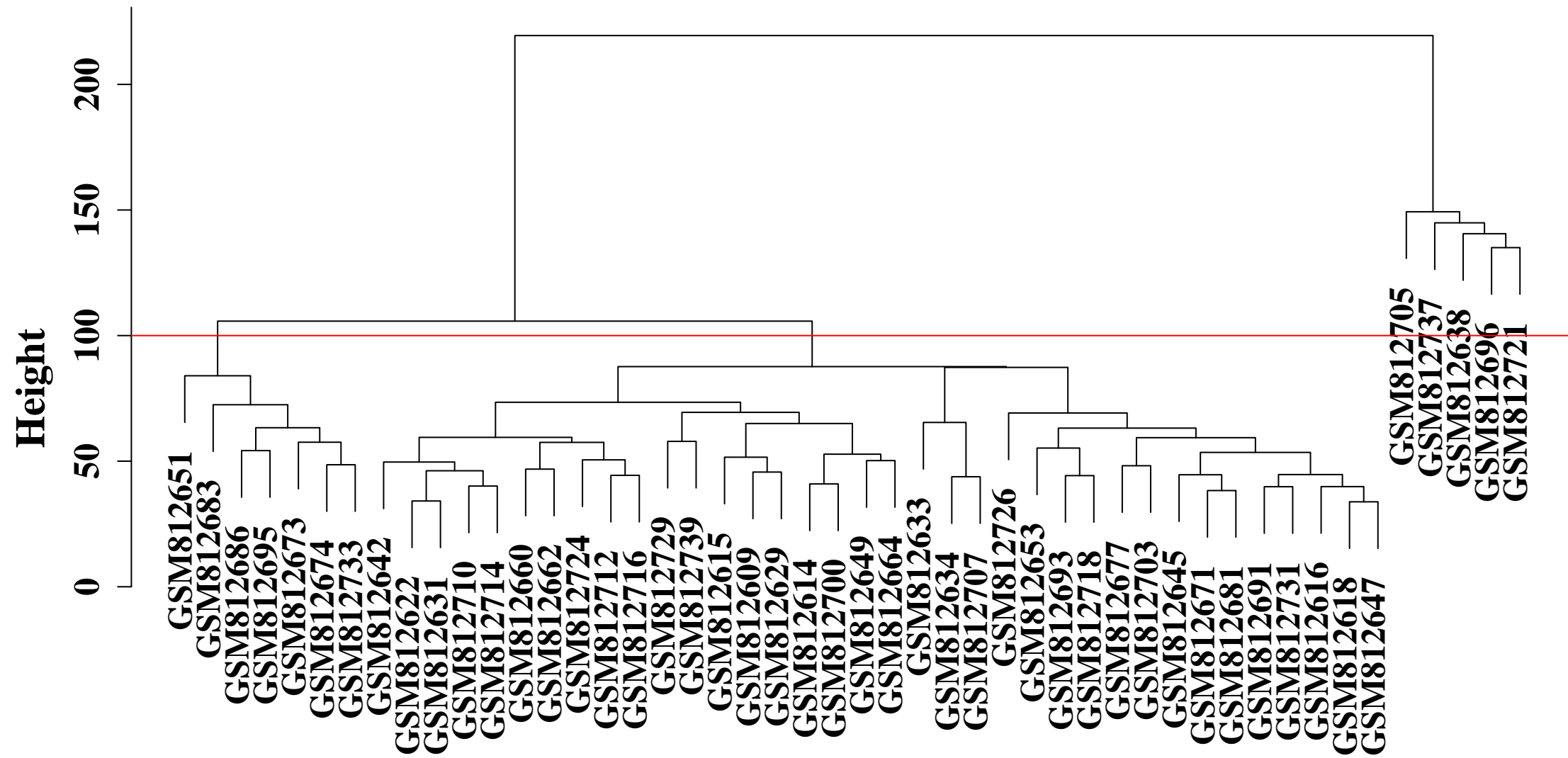

Supplement: Supplementary Table 1 — The primer sequences for PCR. [file DataSheet1.zip › Original data/02_WGCNA/04.sampleClustering.pdf]

# Sample Clustering and trait heatmap

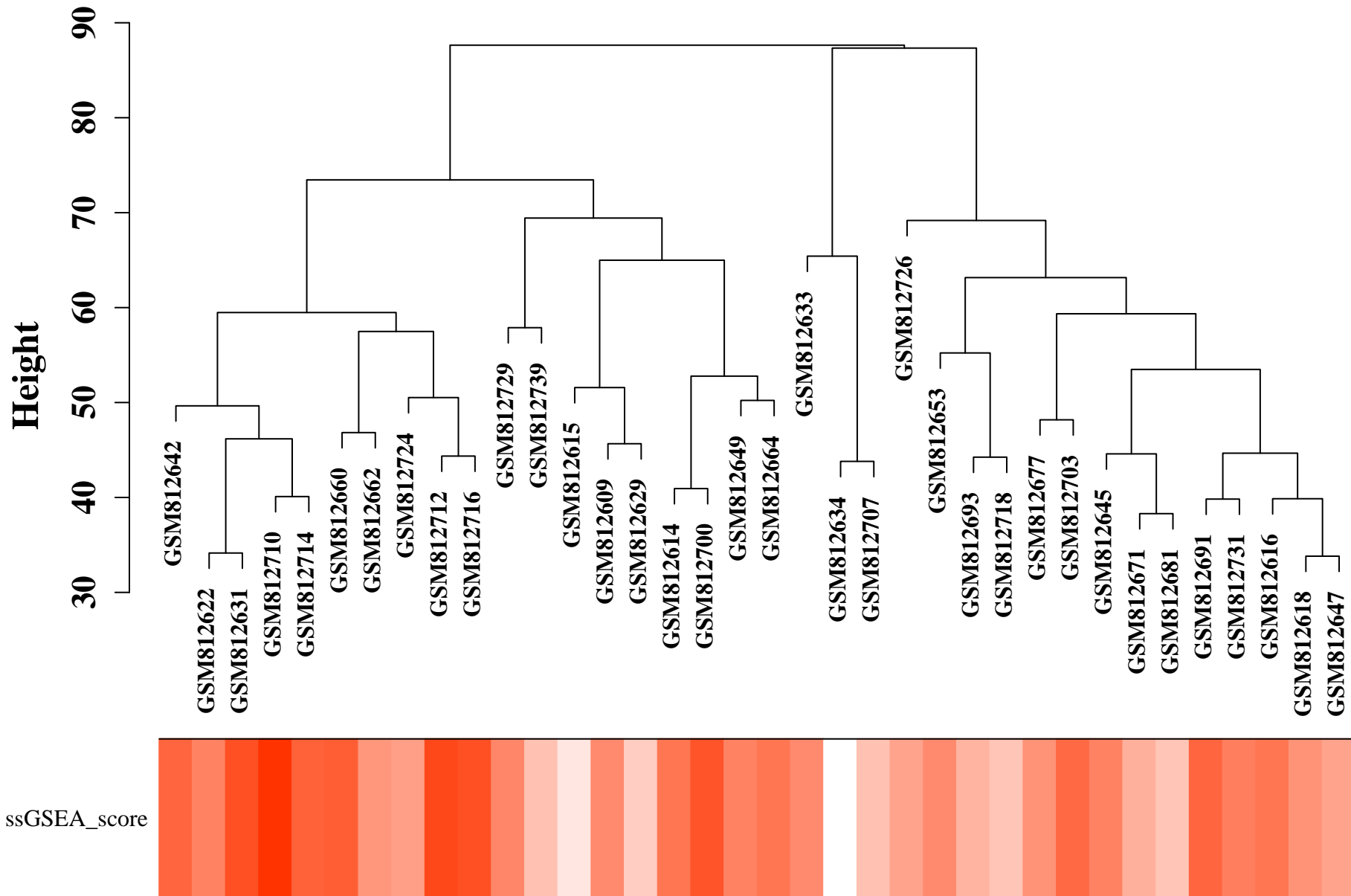

Supplement: Supplementary Table 1 — The primer sequences for PCR. [file DataSheet1.zip › Original data/02_WGCNA/05.sampleClustering2.pdf]

**Scale independence**

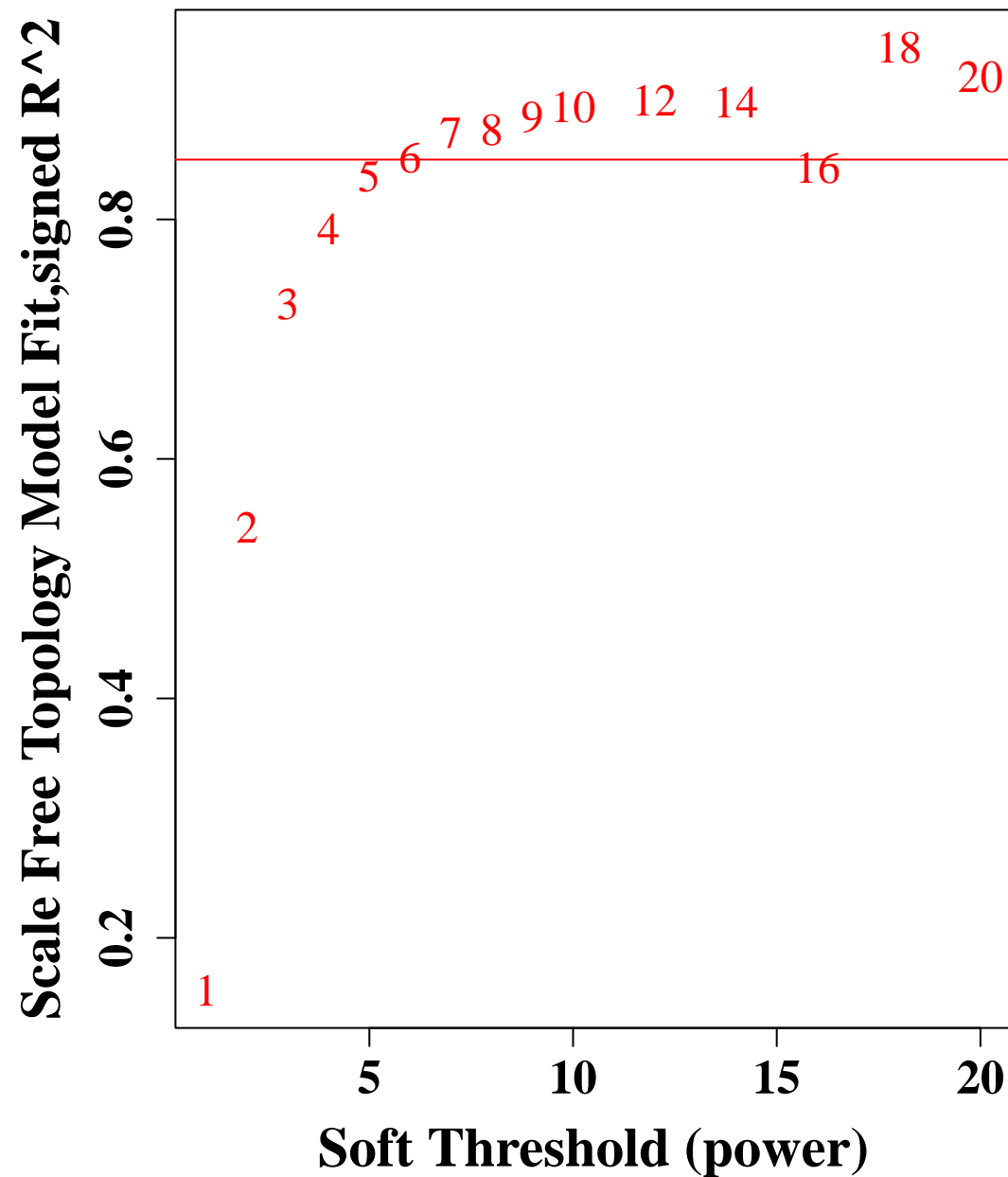

**Mean connectivity**

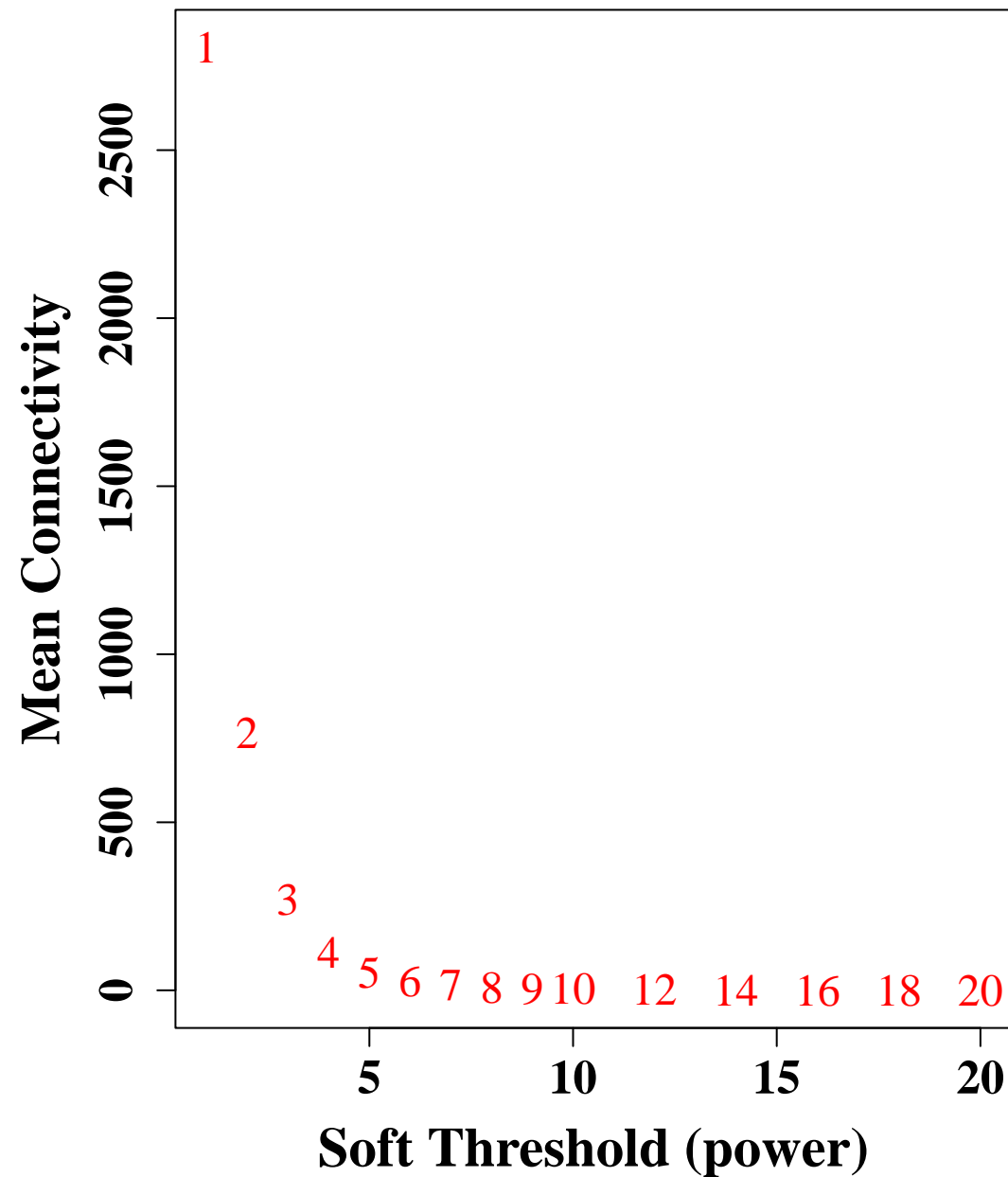

Supplement: Supplementary Table 1 — The primer sequences for PCR. [file DataSheet1.zip › Original data/02_WGCNA/06.softThreshold.pdf]

# Cluster Dendrogram

Height

0.95  
0.90  
0.85  
0.80  
0.75

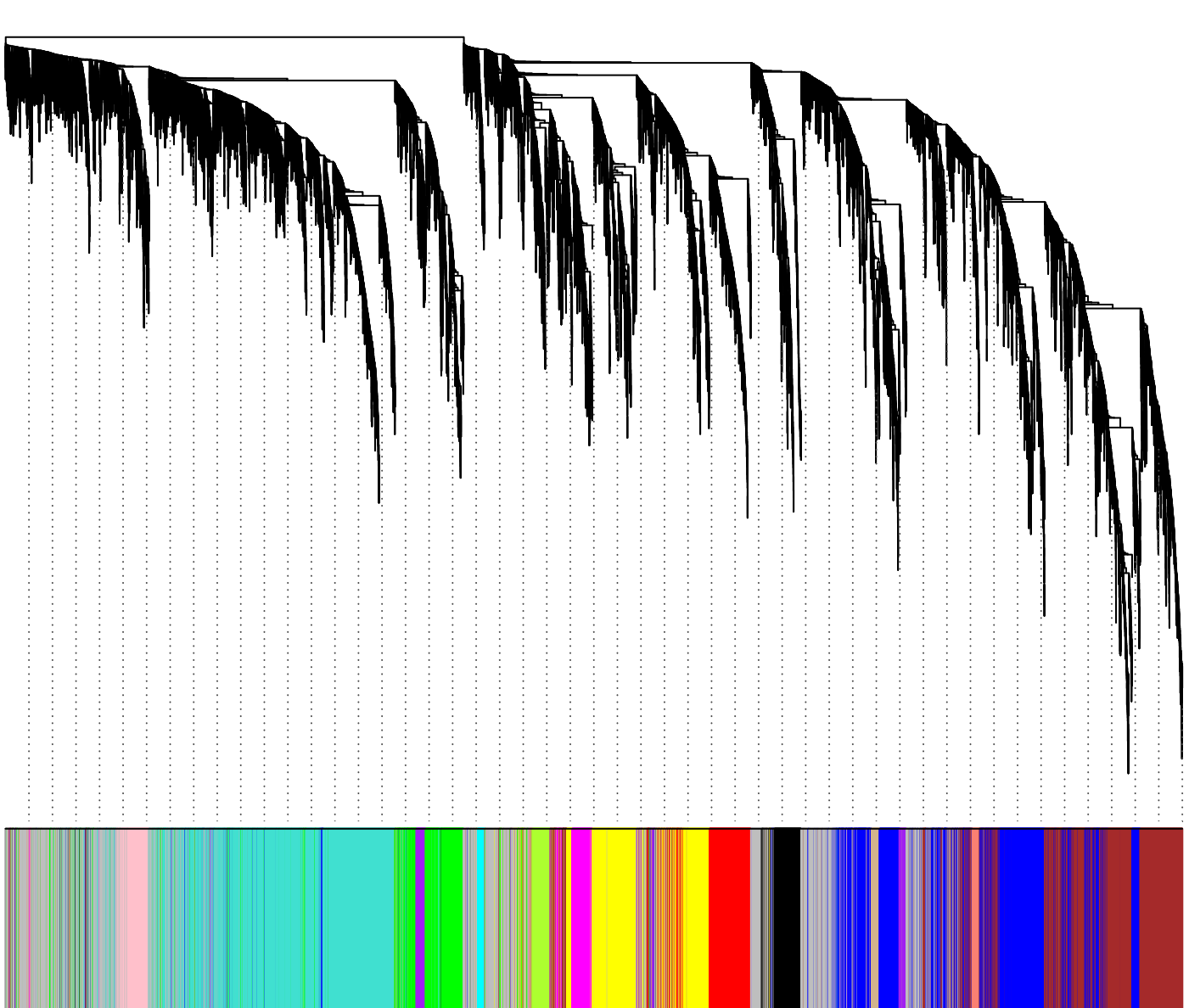

Module colors

Supplement: Supplementary Table 1 — The primer sequences for PCR. [file DataSheet1.zip › Original data/02_WGCNA/07.wgcna.dendroColors.pdf]

Module–trait relationships

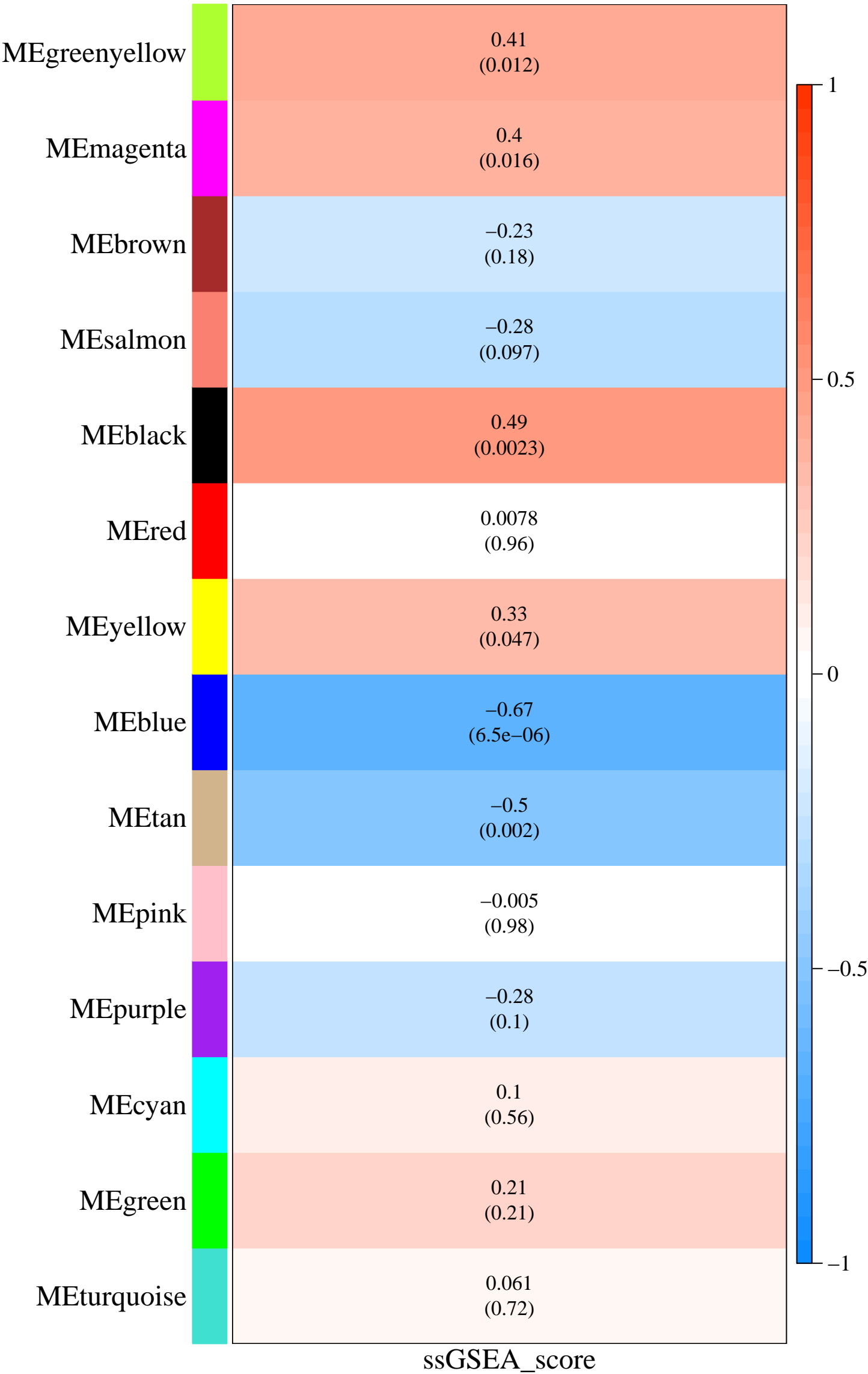

Supplement: Supplementary Table 1 — The primer sequences for PCR. [file DataSheet1.zip › Original data/02_WGCNA/08.wgcna.Module-trait.heatmap.pdf]

WGCNA

DEGs

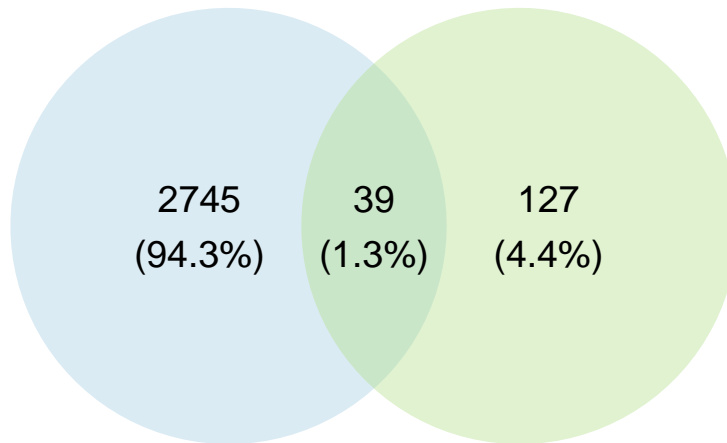

Supplement: Supplementary Table 1 — The primer sequences for PCR. [file DataSheet1.zip › Original data/03_Candidated_genes/02.intersect_genes.pdf]

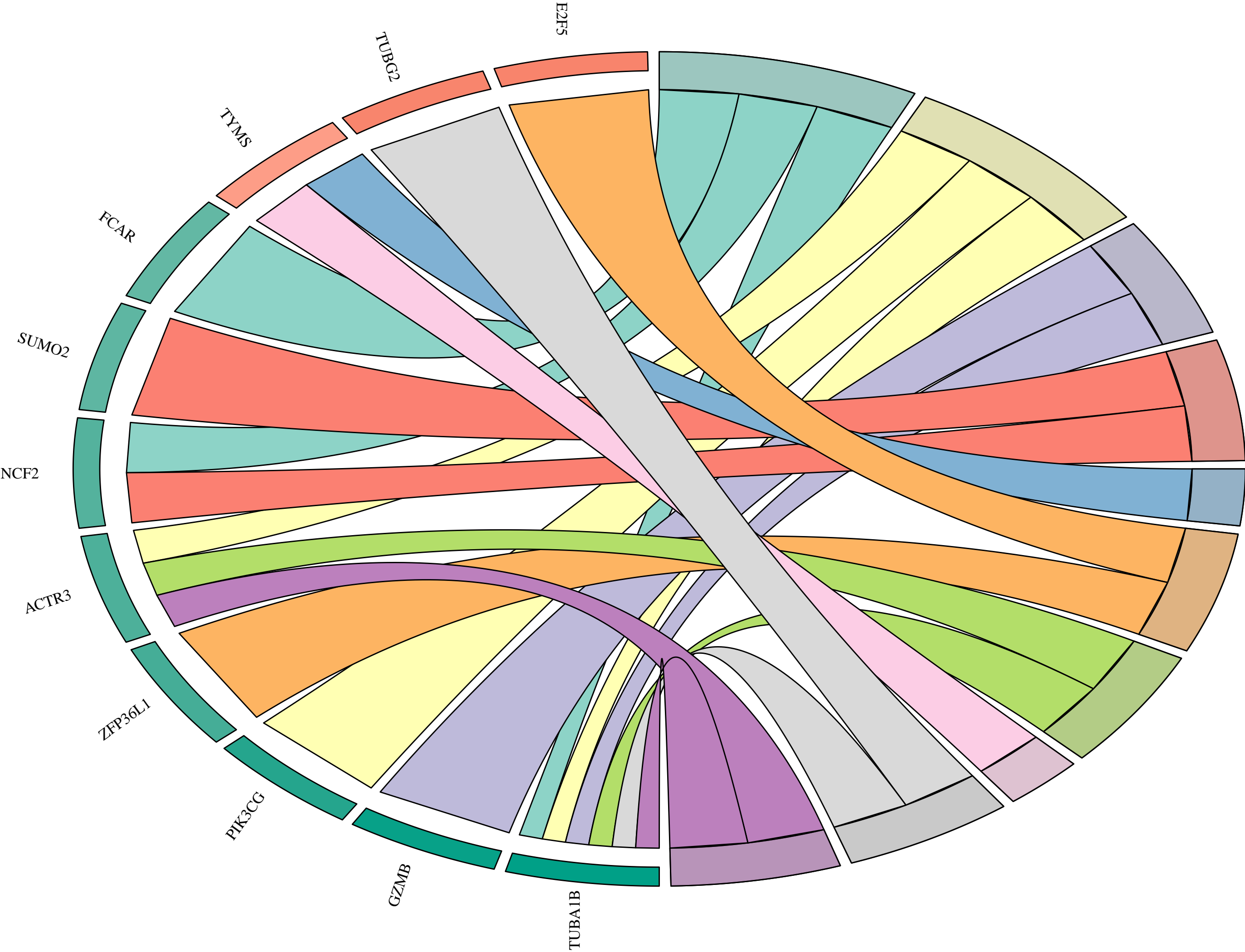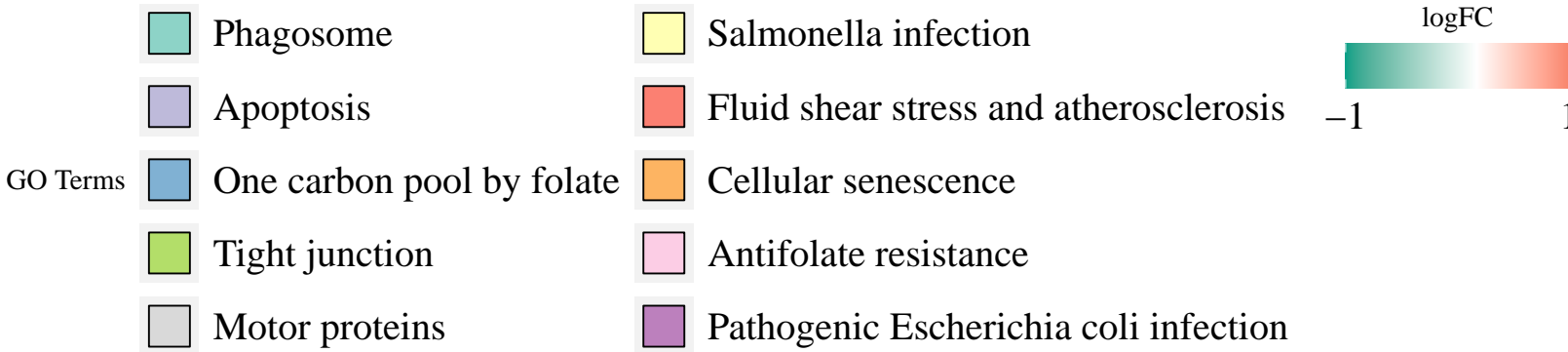

Supplement: Supplementary Table 1 — The primer sequences for PCR. [file DataSheet1.zip › Original data/03_Candidated_genes/04.KEGG.pdf]

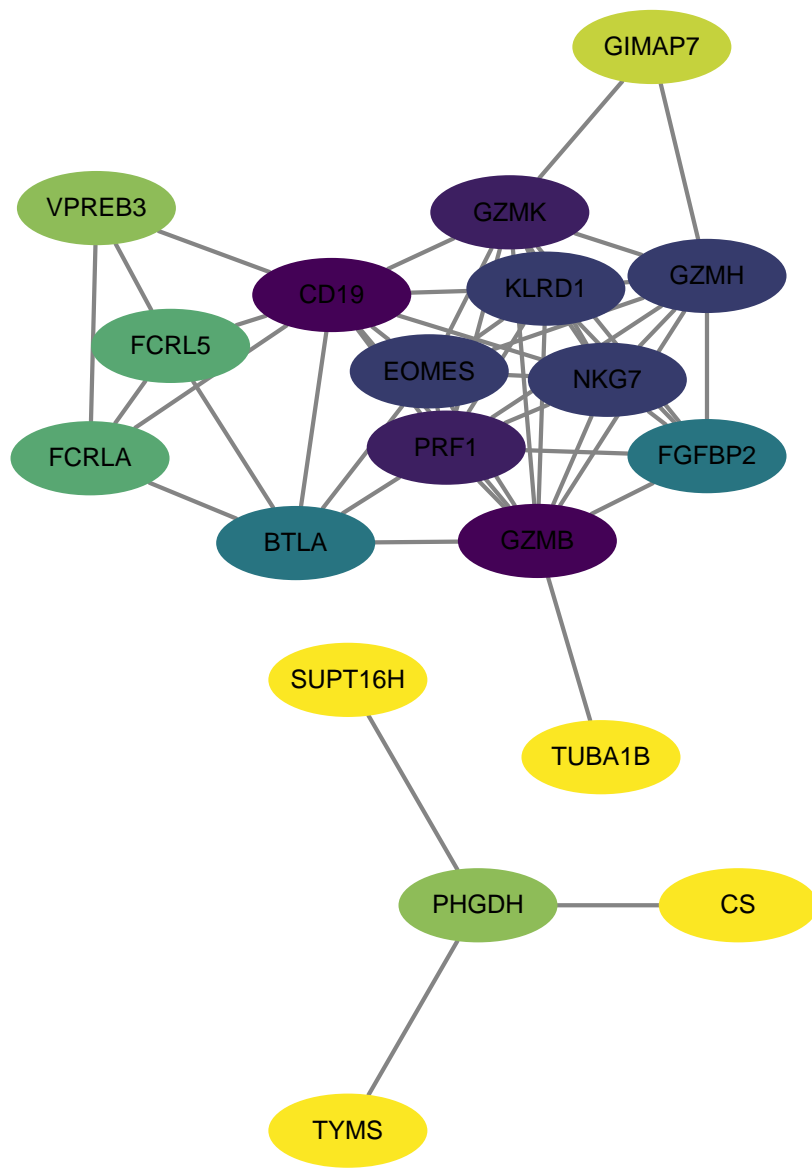

Supplement: Supplementary Table 1 — The primer sequences for PCR. [file DataSheet1.zip › Original data/04_PPI/ppi.pdf]

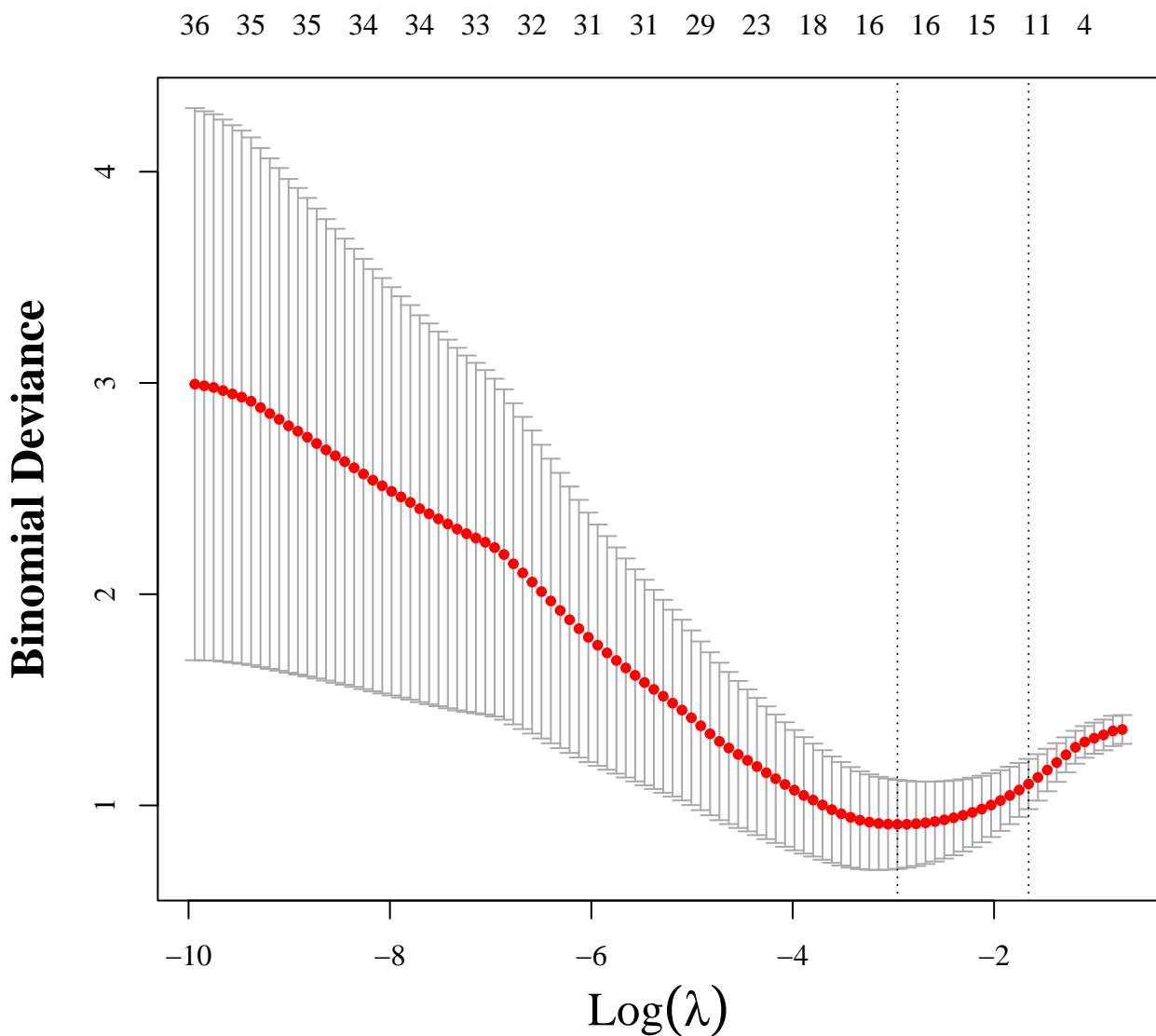

Supplement: Supplementary Table 1 — The primer sequences for PCR. [file DataSheet1.zip › Original data/05_Mobel/01.LASSO.CV.pdf]

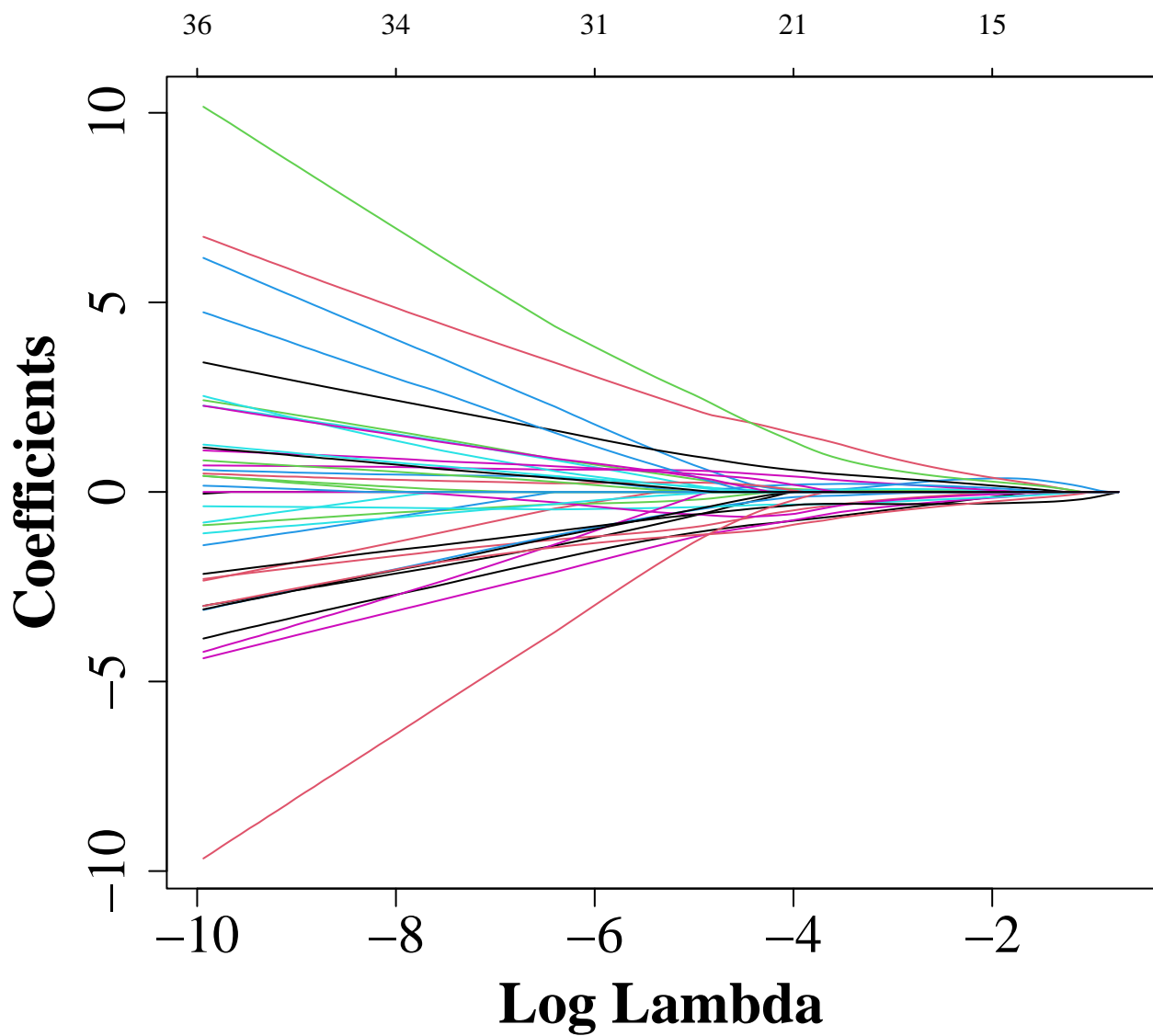

Supplement: Supplementary Table 1 — The primer sequences for PCR. [file DataSheet1.zip › Original data/05_Mobel/02.LASSO.Coef.pdf]

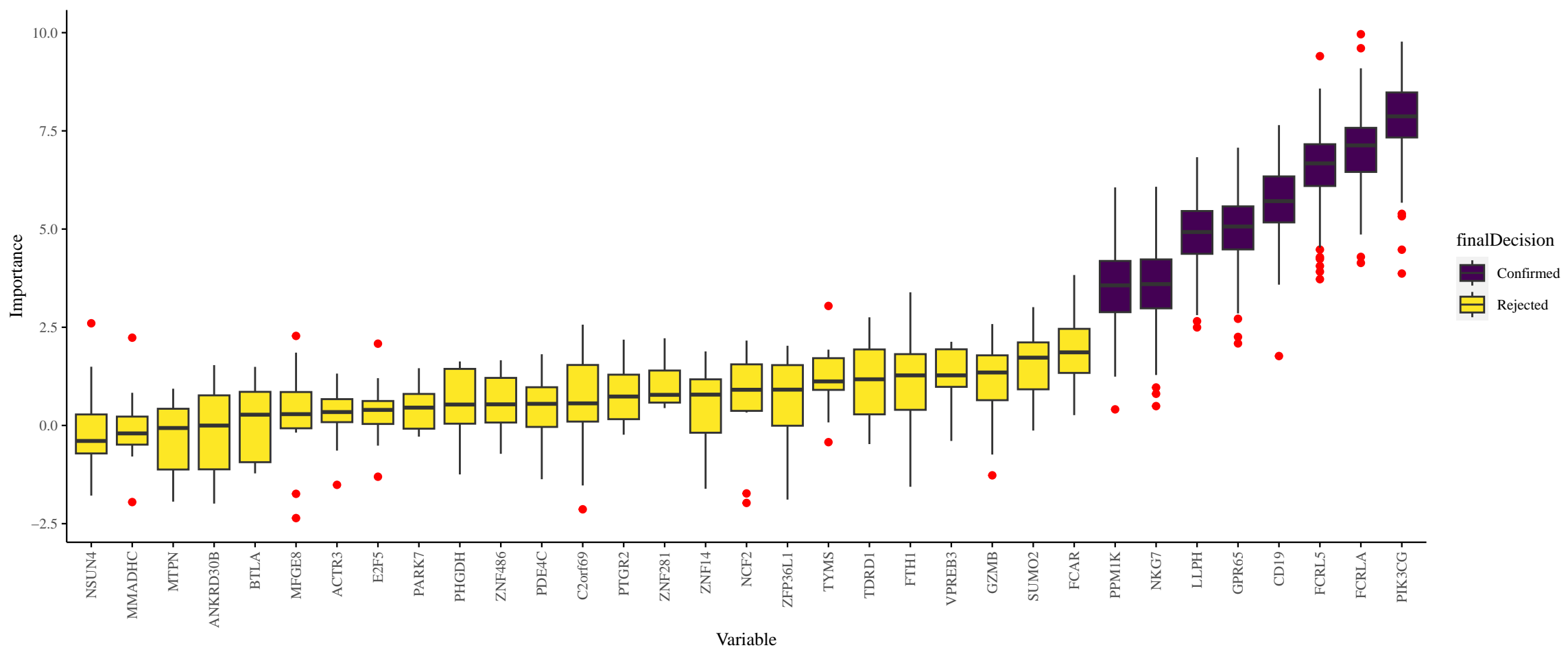

Supplement: Supplementary Table 1 — The primer sequences for PCR. [file DataSheet1.zip › Original data/05_Mobel/05.Boruta.pdf]

# XGBoost

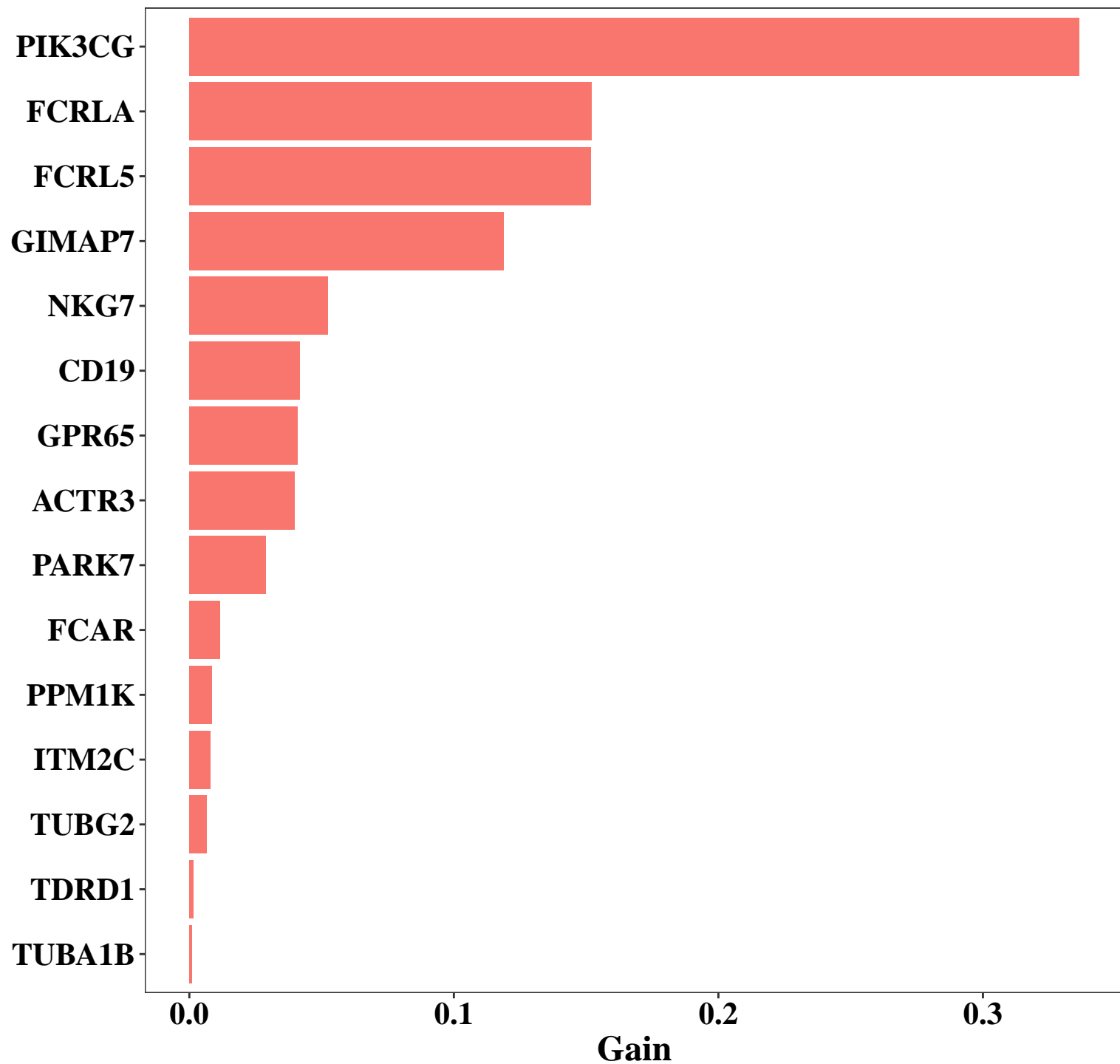

Supplement: Supplementary Table 1 — The primer sequences for PCR. [file DataSheet1.zip › Original data/05_Mobel/06.XGBoost_importance.pdf]

XGBoost

Lasso

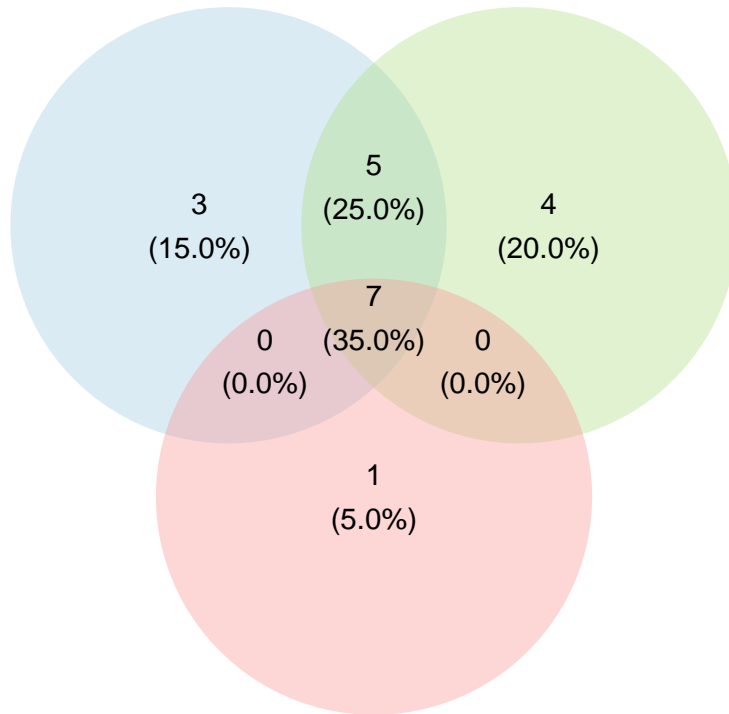

Boruta

Supplement: Supplementary Table 1 — The primer sequences for PCR. [file DataSheet1.zip › Original data/05_Mobel/08.gene_venn.pdf]

GSE32707

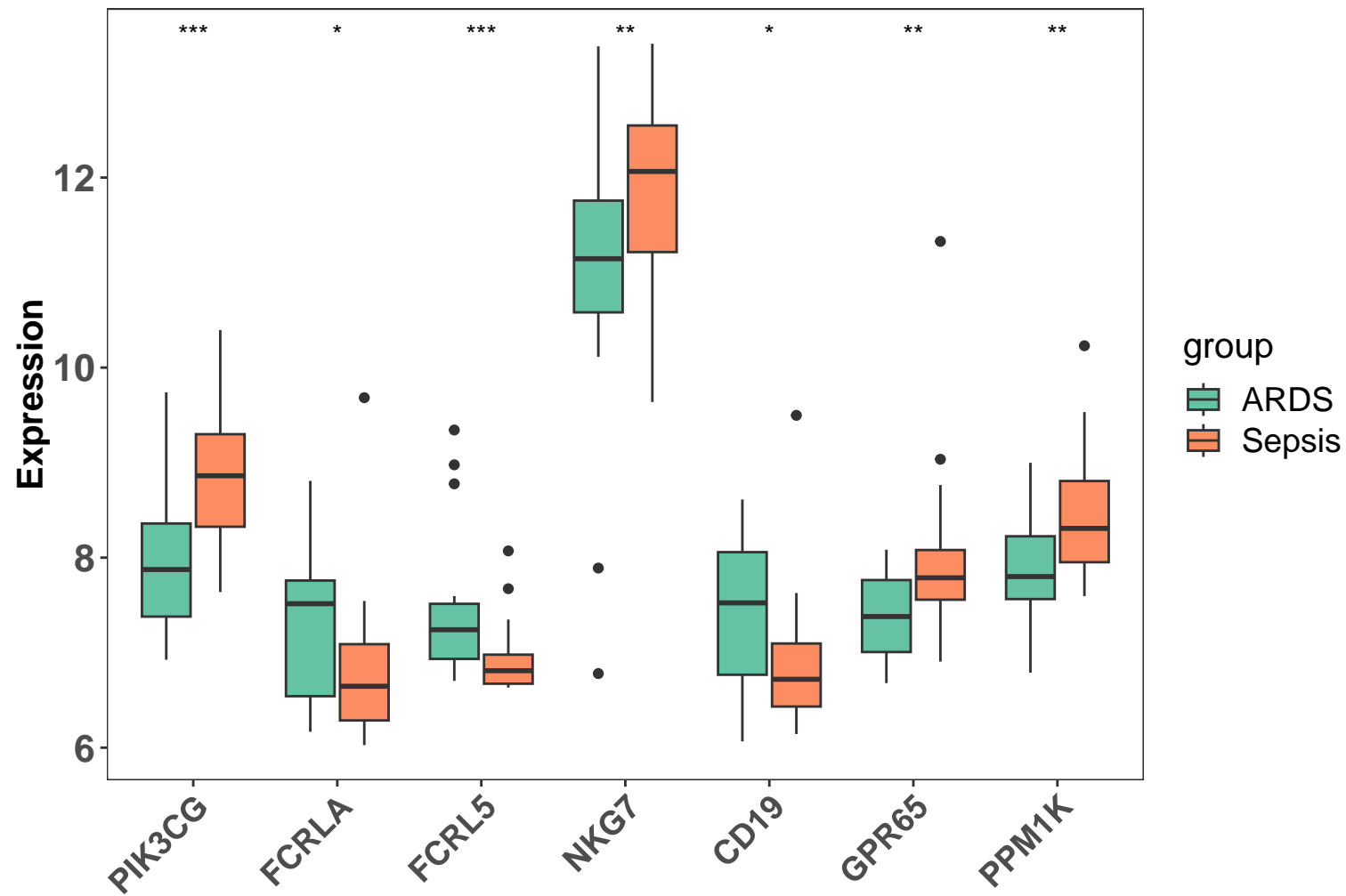

Supplement: Supplementary Table 1 — The primer sequences for PCR. [file DataSheet1.zip › Original data/06_Exp/02.verify_exp_boxplot_GSE32707.pdf]

GSE66890

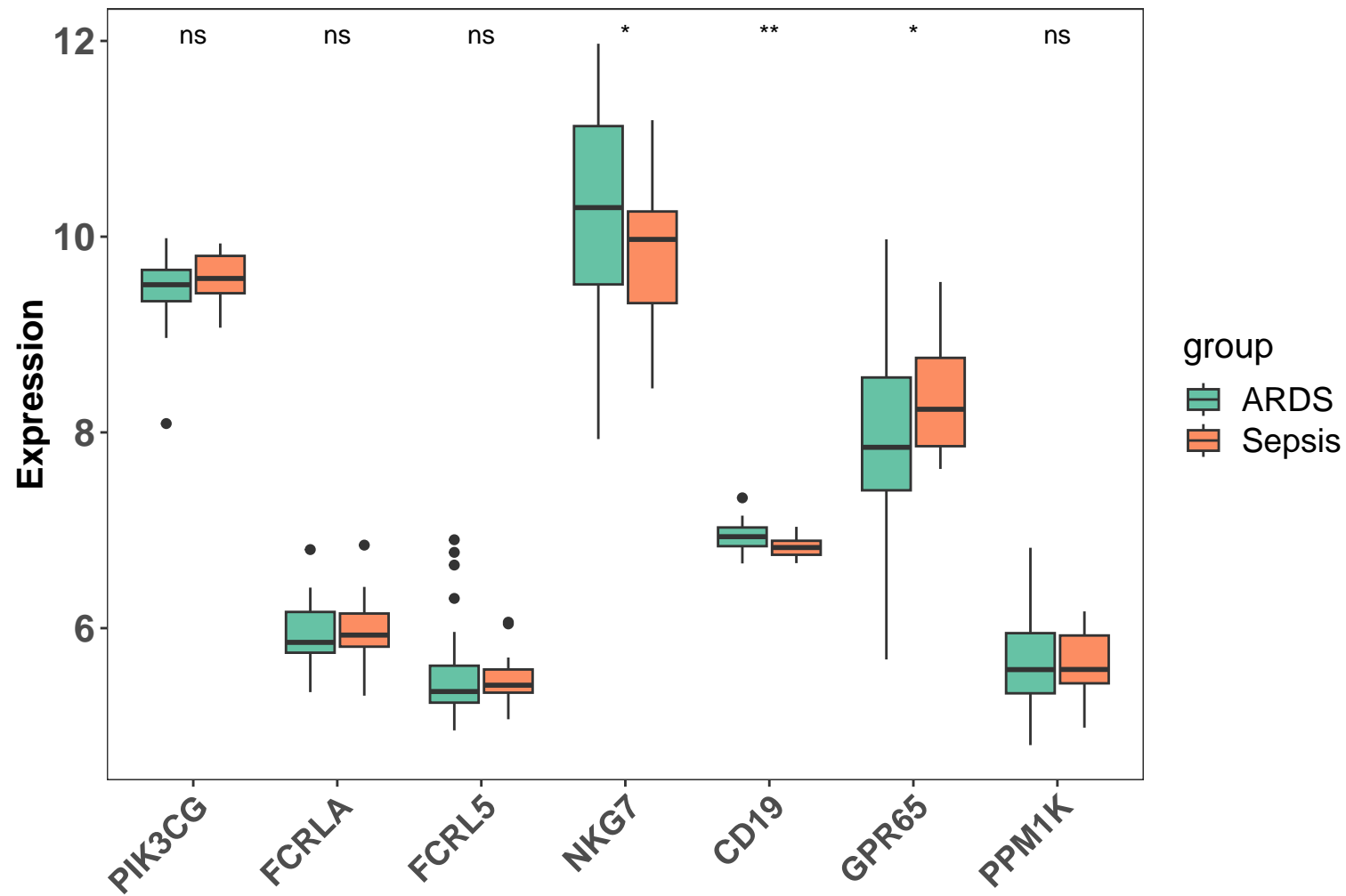

Supplement: Supplementary Table 1 — The primer sequences for PCR. [file DataSheet1.zip › Original data/06_Exp/02.verify_exp_boxplot_GSE66890.pdf]

**Points**

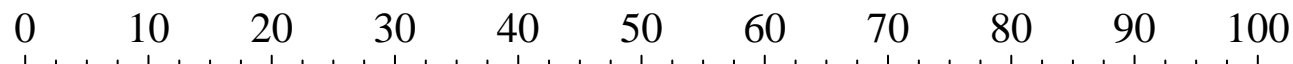

**CD19**

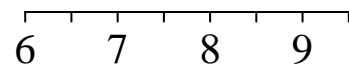

**GPR65**

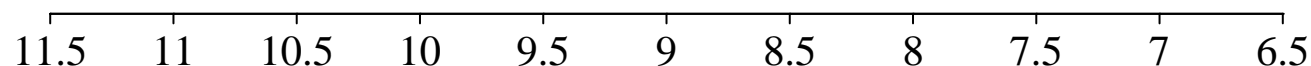

**Total Points**

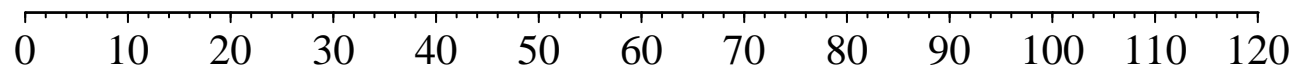

**Risk of ARDS**

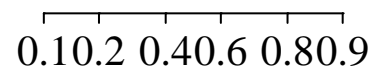

Supplement: Supplementary Table 1 — The primer sequences for PCR. [file DataSheet1.zip › Original data/07_Logistic/02.nomogram_line_points.pdf]

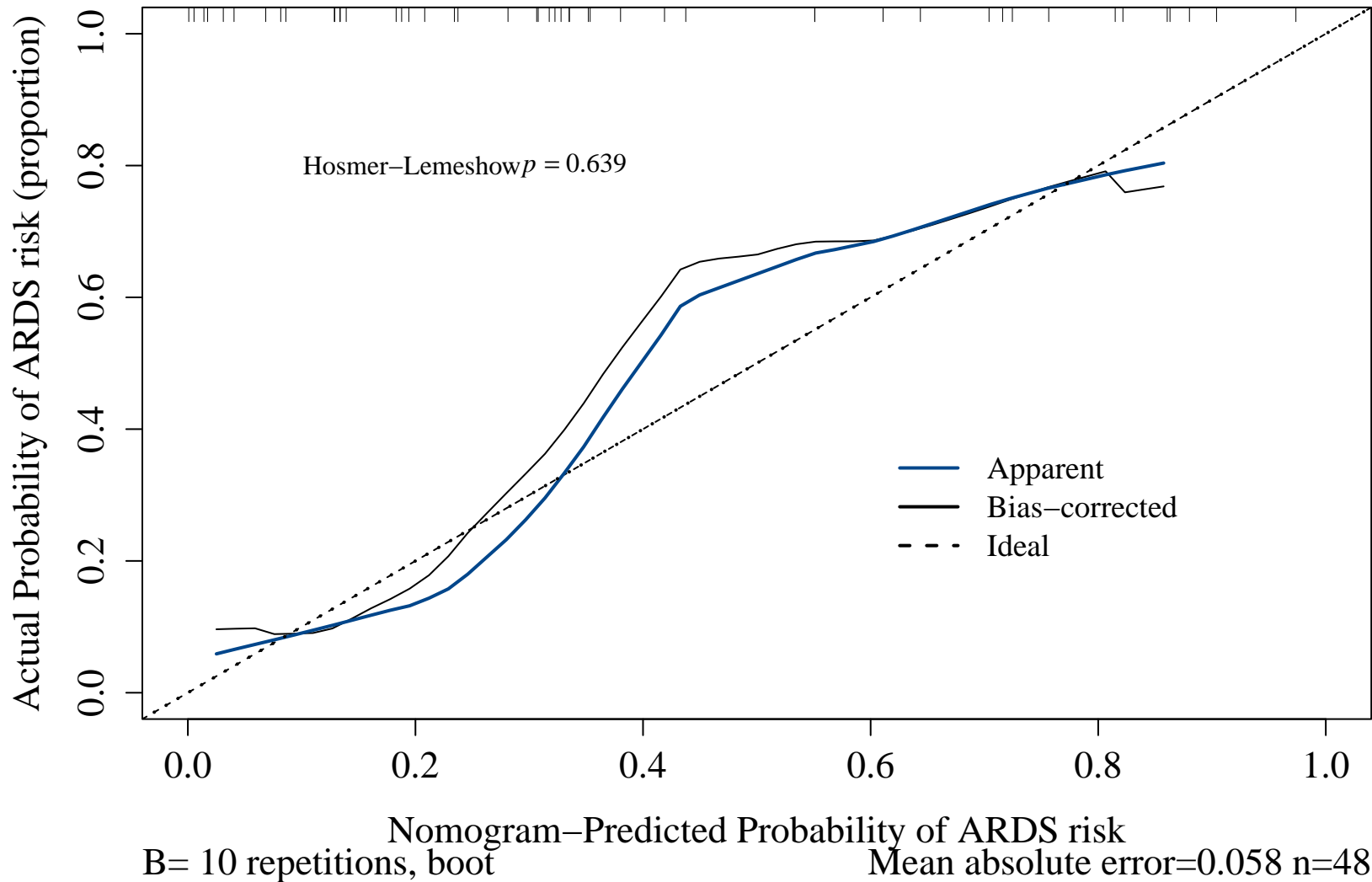

Supplement: Supplementary Table 1 — The primer sequences for PCR. [file DataSheet1.zip › Original data/07_Logistic/03.calibrate.pdf]

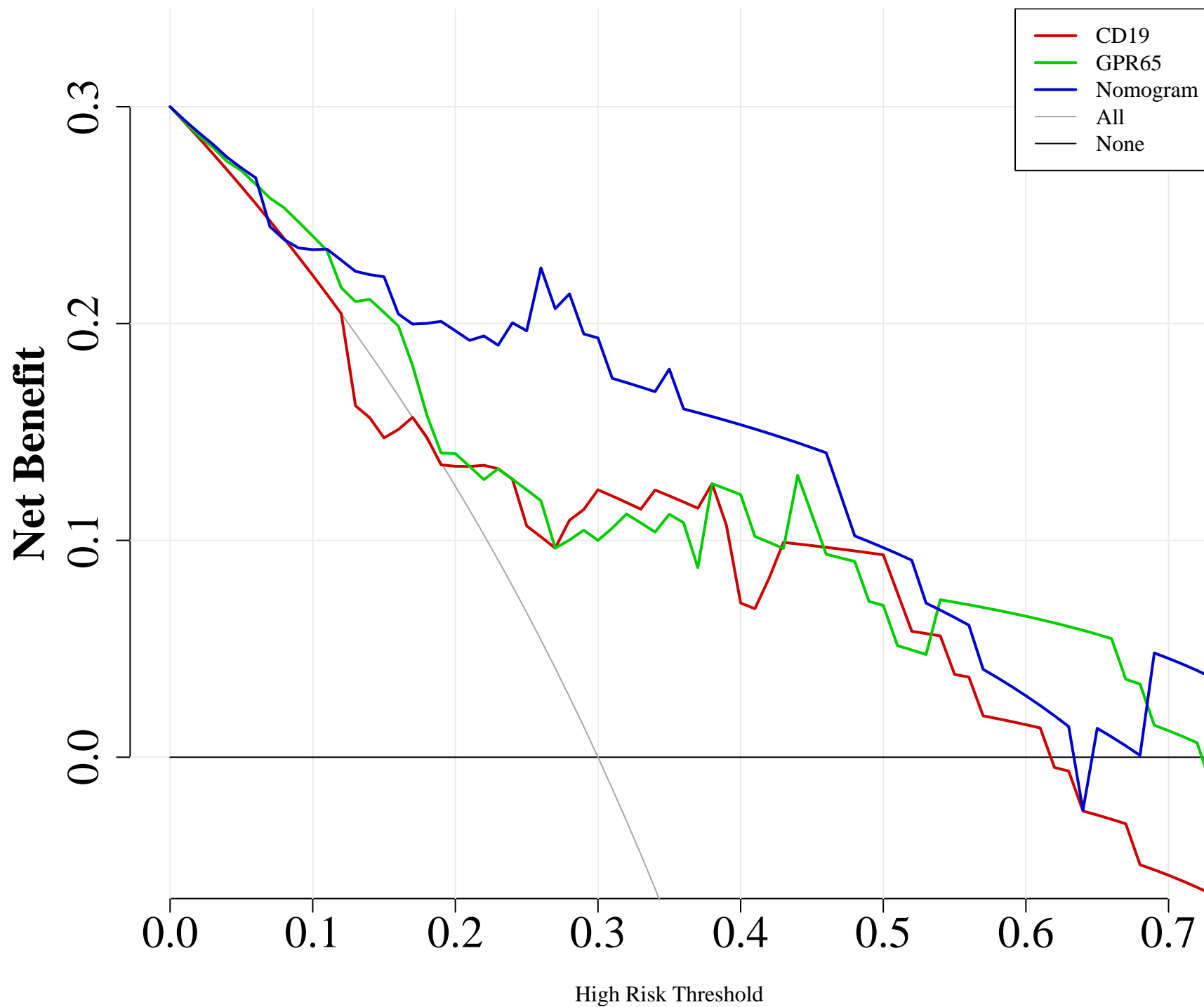

Supplement: Supplementary Table 1 — The primer sequences for PCR. [file DataSheet1.zip › Original data/07_Logistic/04.DCA.pdf]

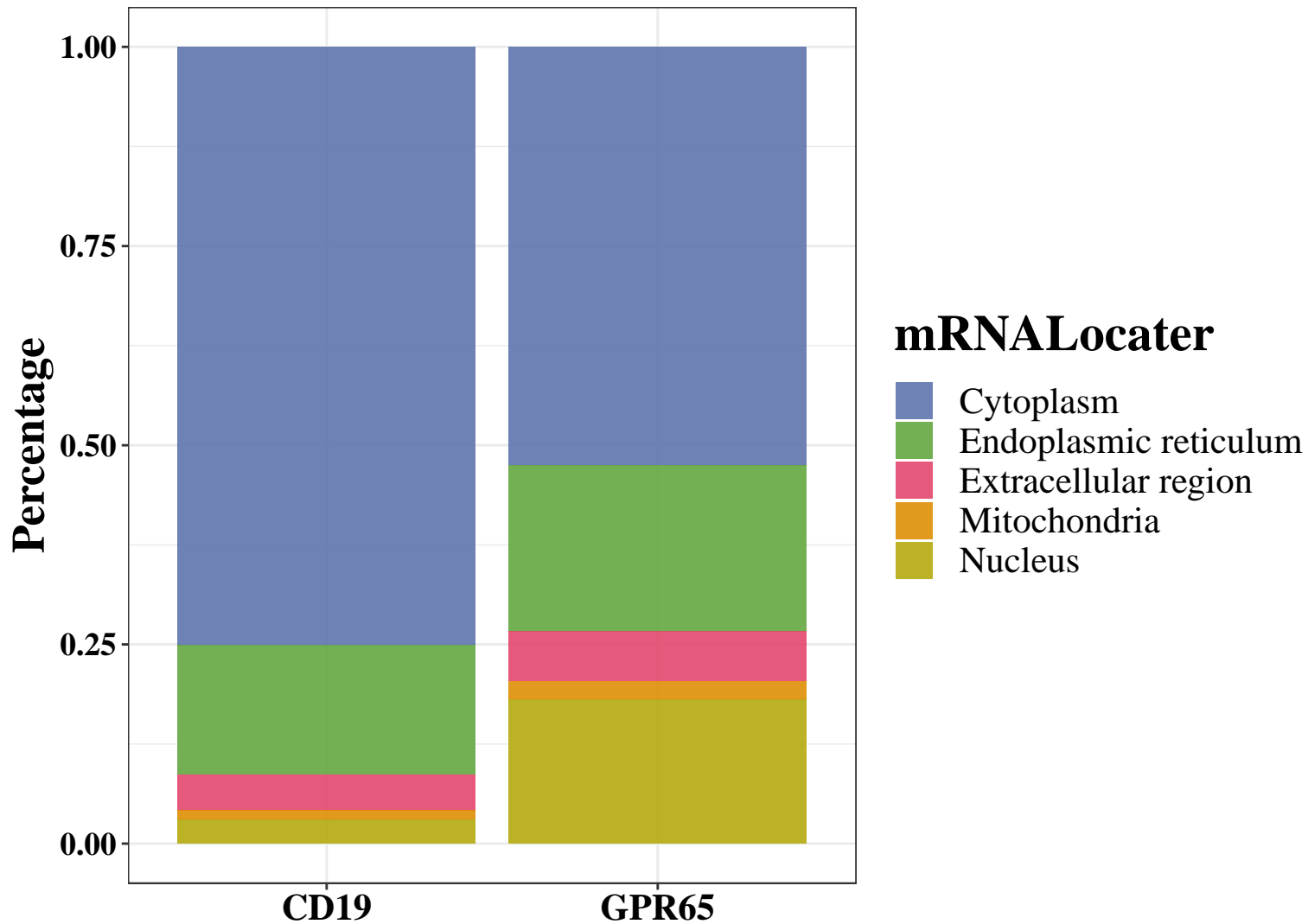

Supplement: Supplementary Table 1 — The primer sequences for PCR. [file DataSheet1.zip › Original data/08_mRNALocater/01.mRNAlocator.pdf]

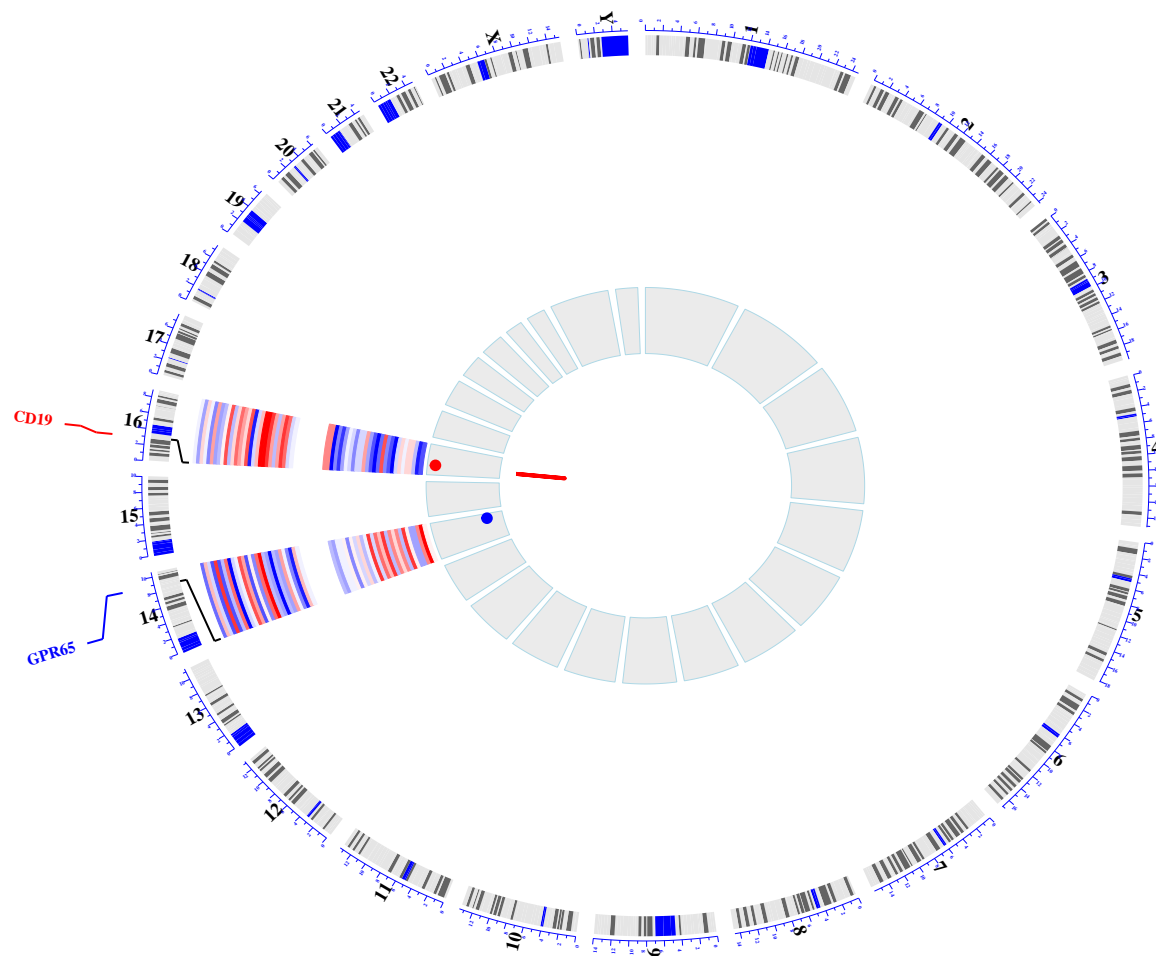

Supplement: Supplementary Table 1 — The primer sequences for PCR. [file DataSheet1.zip › Original data/08_mRNALocater/02.gene_OmicCircos.pdf]

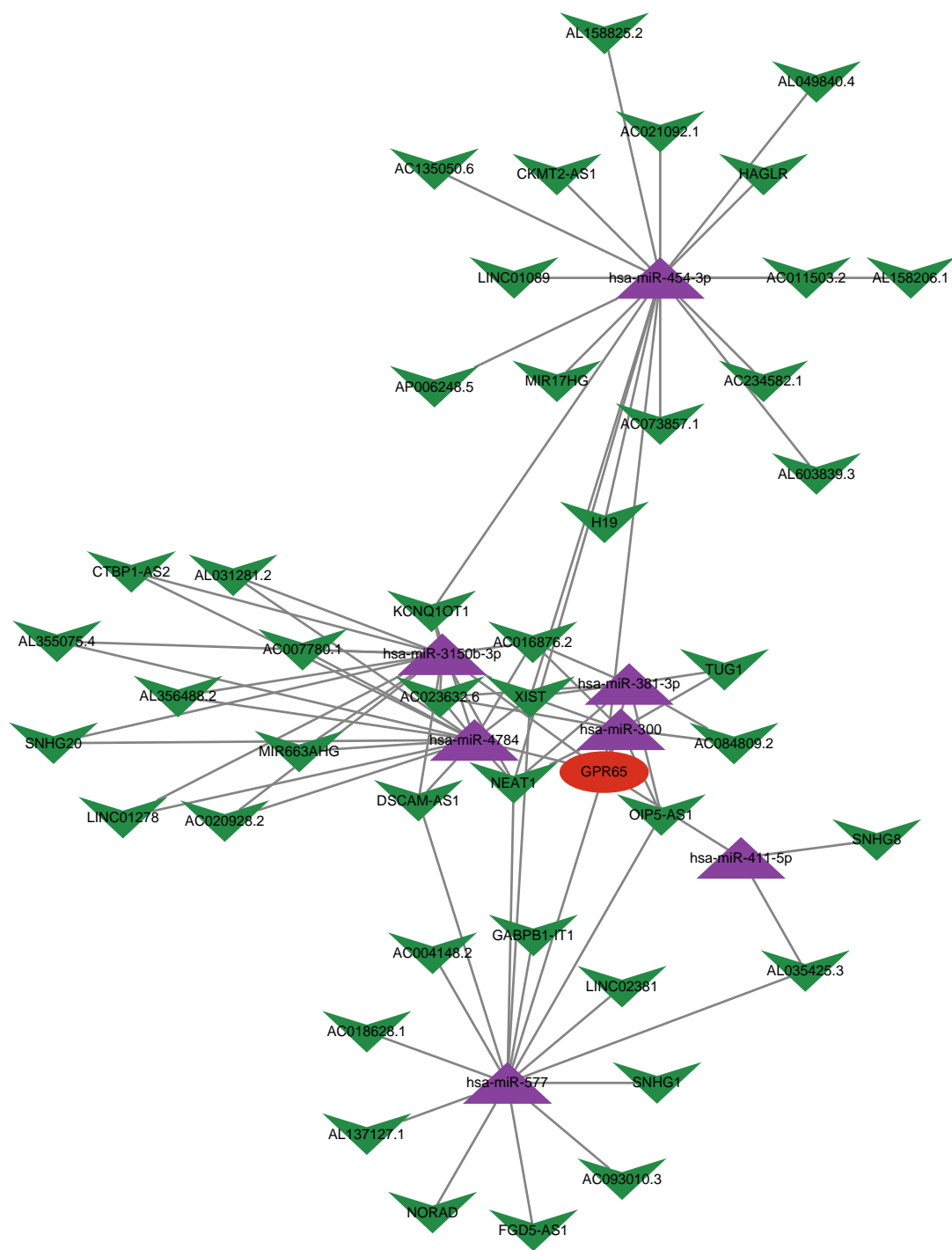

Supplement: Supplementary Table 1 — The primer sequences for PCR. [file DataSheet1.zip › Original data/09_ceRNA/ceRNA.pdf]

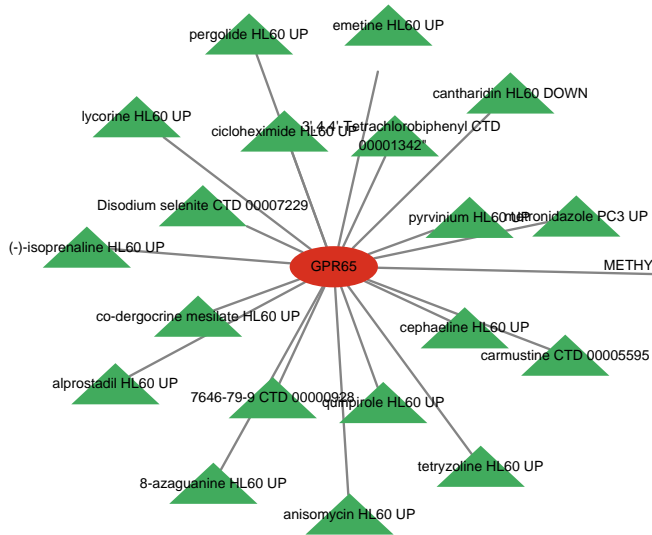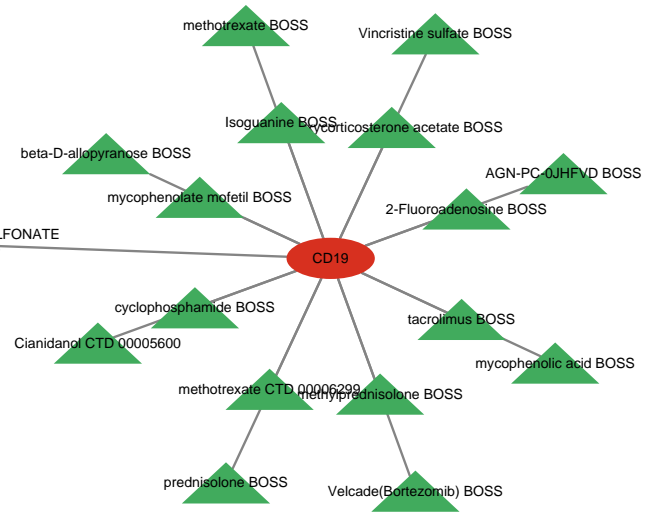

Supplement: Supplementary Table 1 — The primer sequences for PCR. [file DataSheet1.zip › Original data/11_drug_target/drug.pdf]

**nFeature\_RNA**

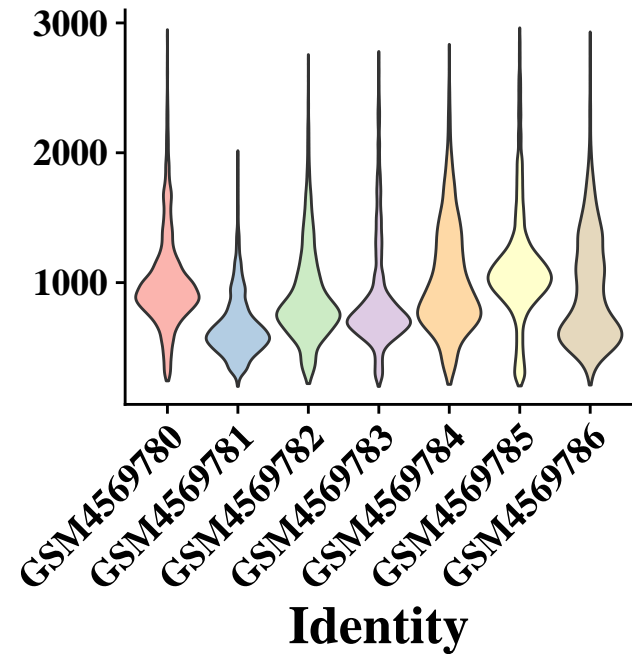

**nCount\_RNA**

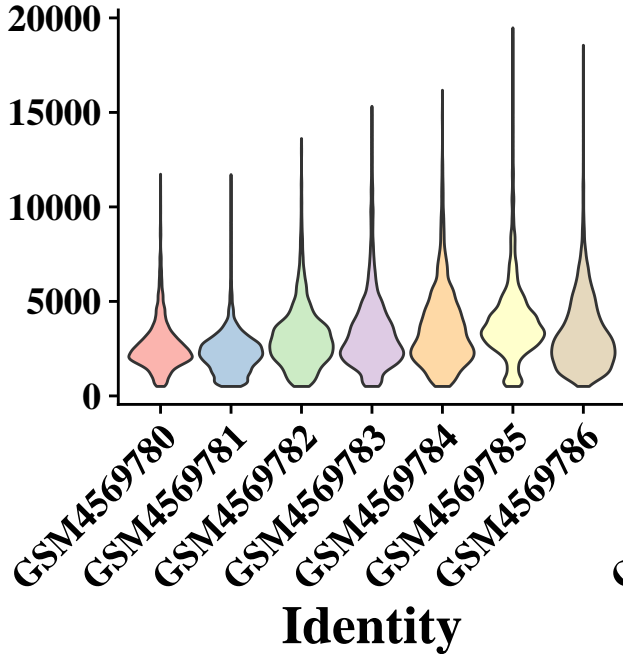

**percent.mt**

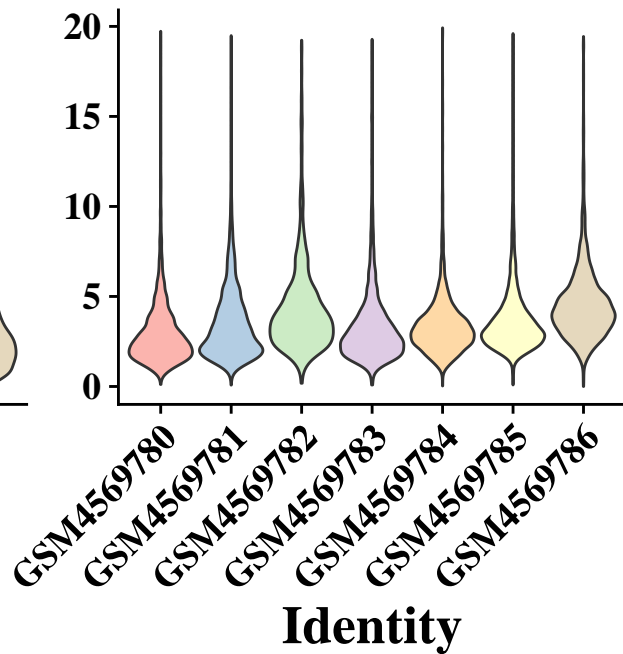

Supplement: Supplementary Table 1 — The primer sequences for PCR. [file DataSheet1.zip › Original data/12_Single_cell/01.vlnplot_before_qc.pdf]

**nFeature\_RNA**

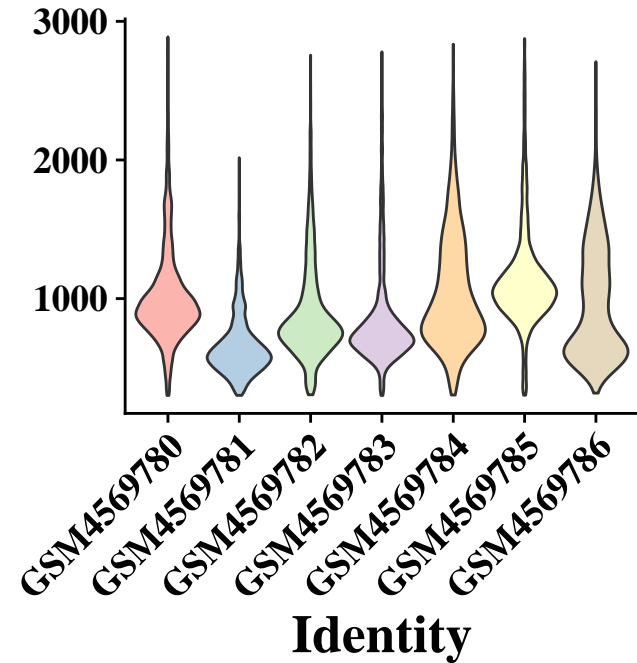

**nCount\_RNA**

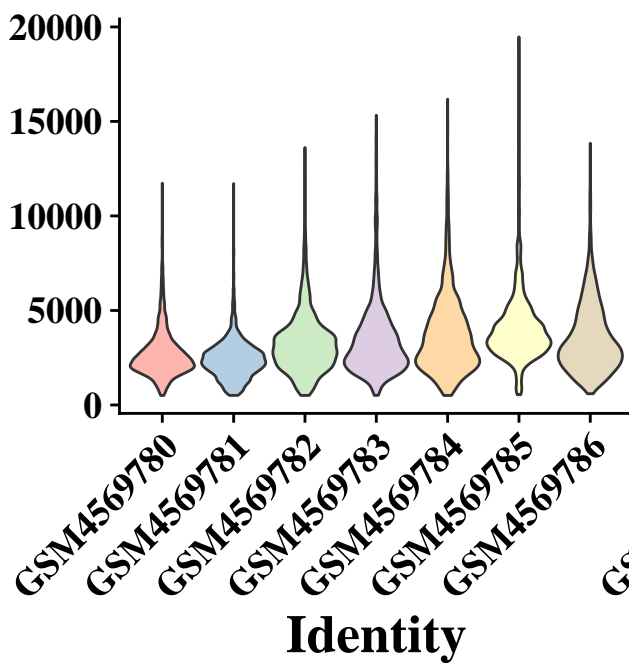

**percent.mt**

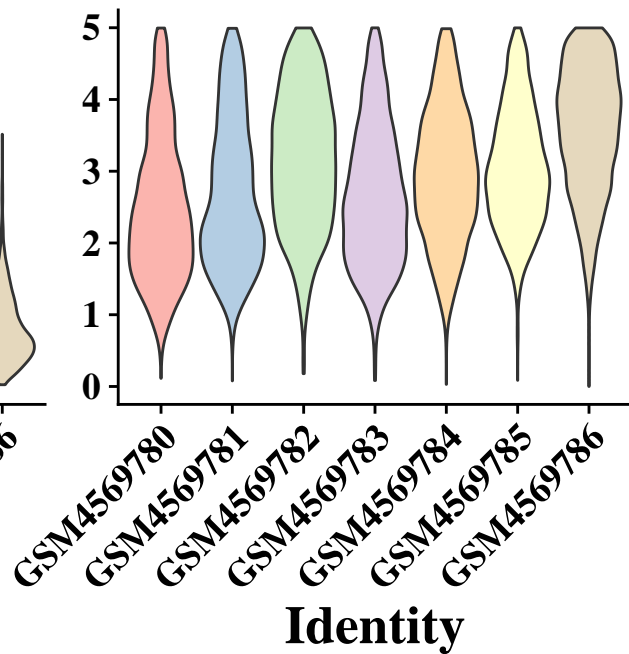

Supplement: Supplementary Table 1 — The primer sequences for PCR. [file DataSheet1.zip › Original data/12_Single_cell/02.vlnplot_after_qc.pdf]

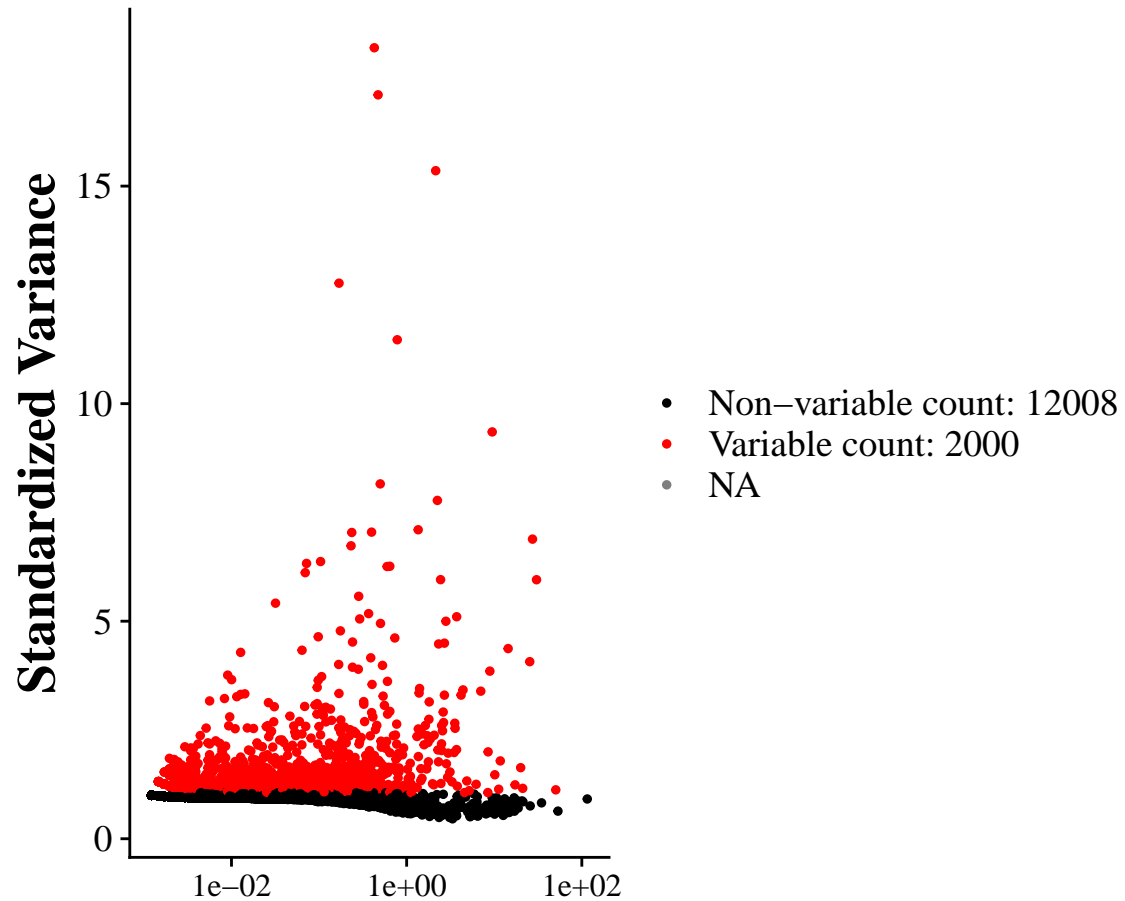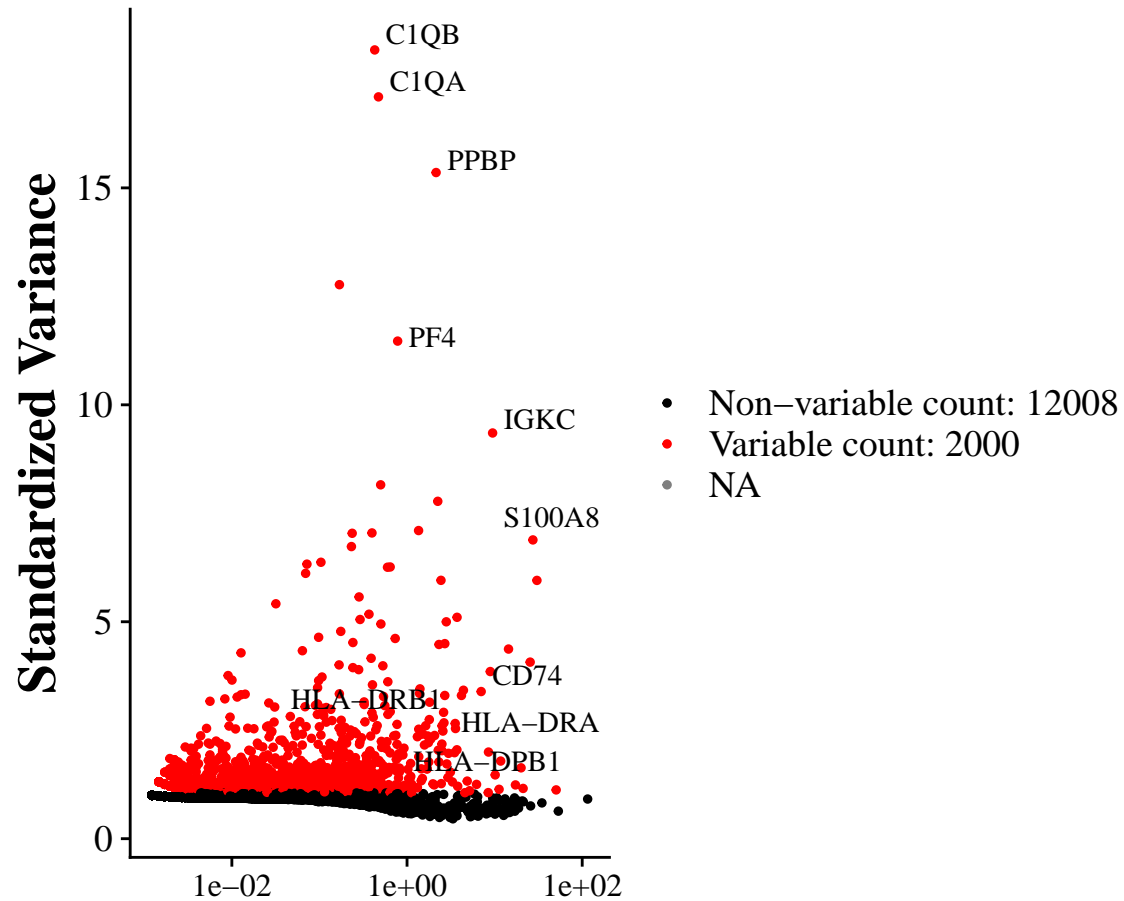

Supplement: Supplementary Table 1 — The primer sequences for PCR. [file DataSheet1.zip › Original data/12_Single_cell/03.featureVar.pdf]

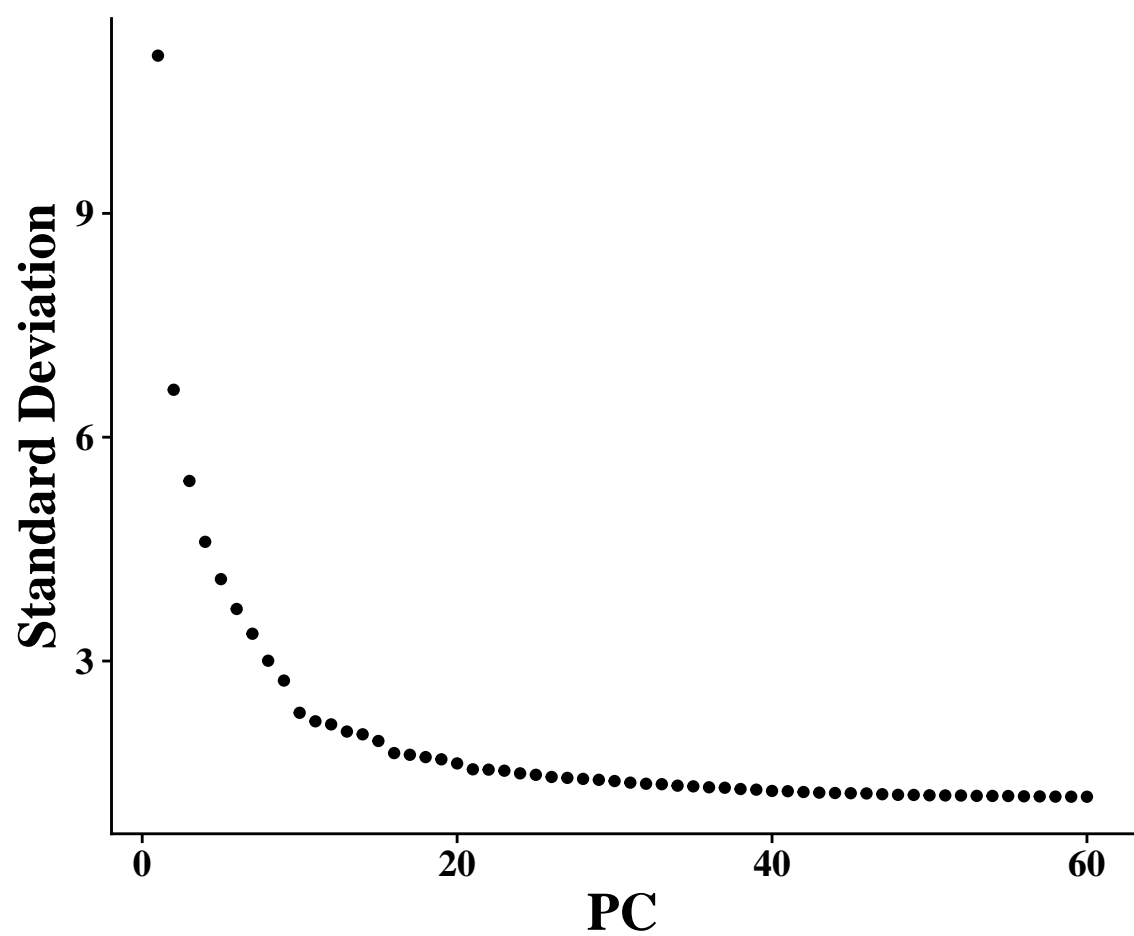

Supplement: Supplementary Table 1 — The primer sequences for PCR. [file DataSheet1.zip › Original data/12_Single_cell/04.ElbowPlot.pdf]

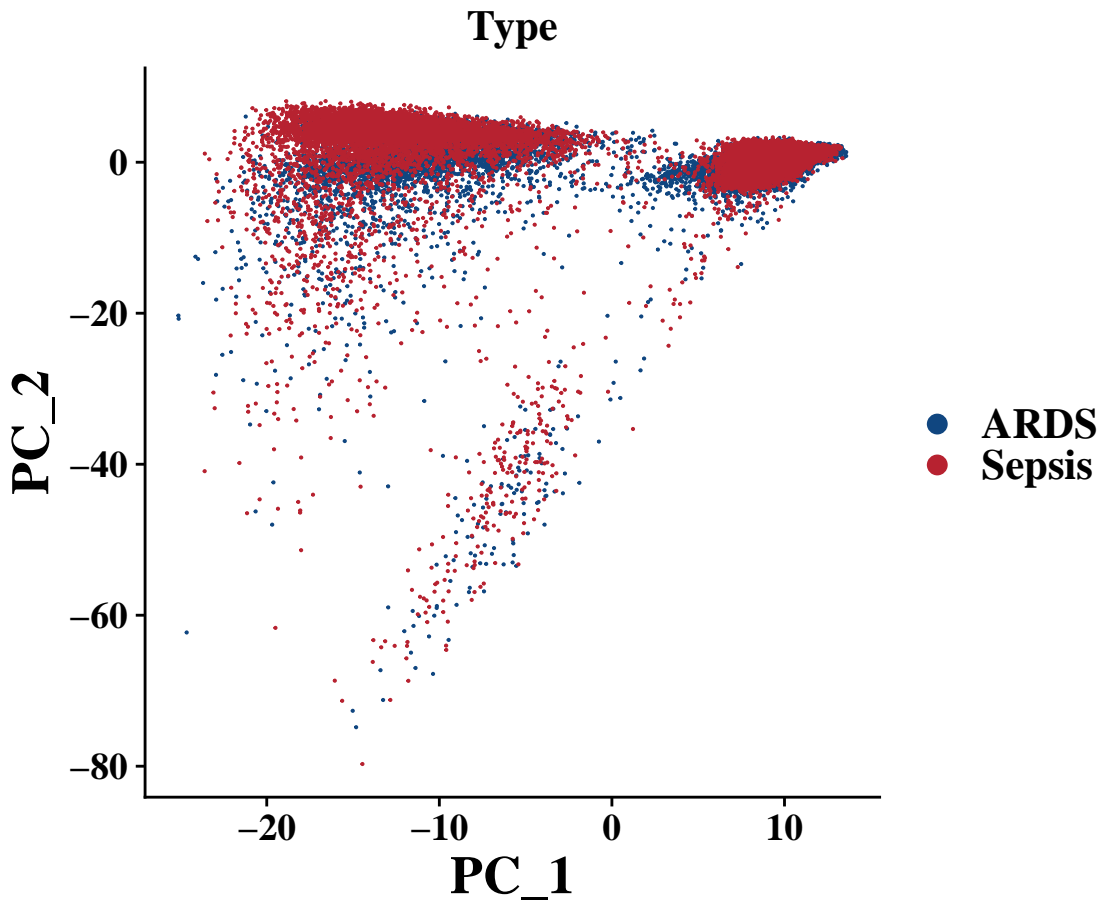

Supplement: Supplementary Table 1 — The primer sequences for PCR. [file DataSheet1.zip › Original data/12_Single_cell/05.PCA.pdf]

Sample

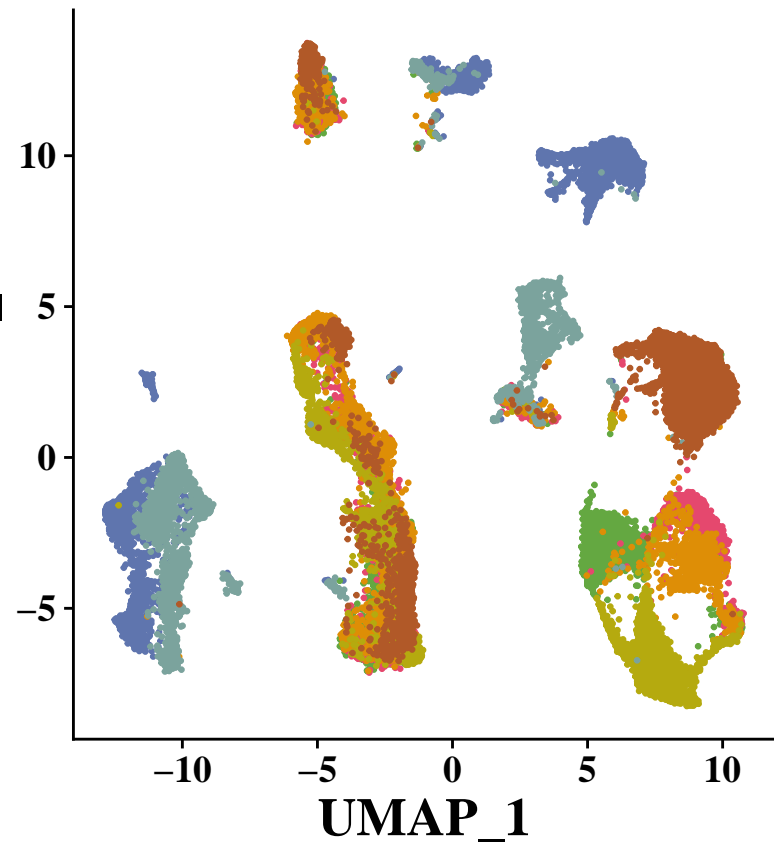

Type

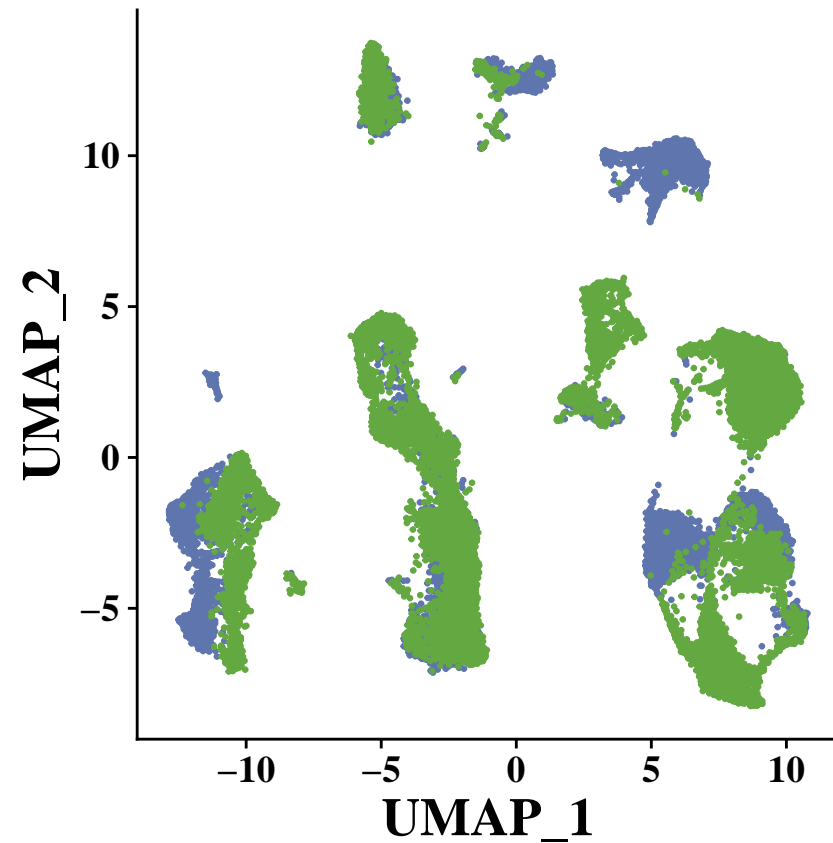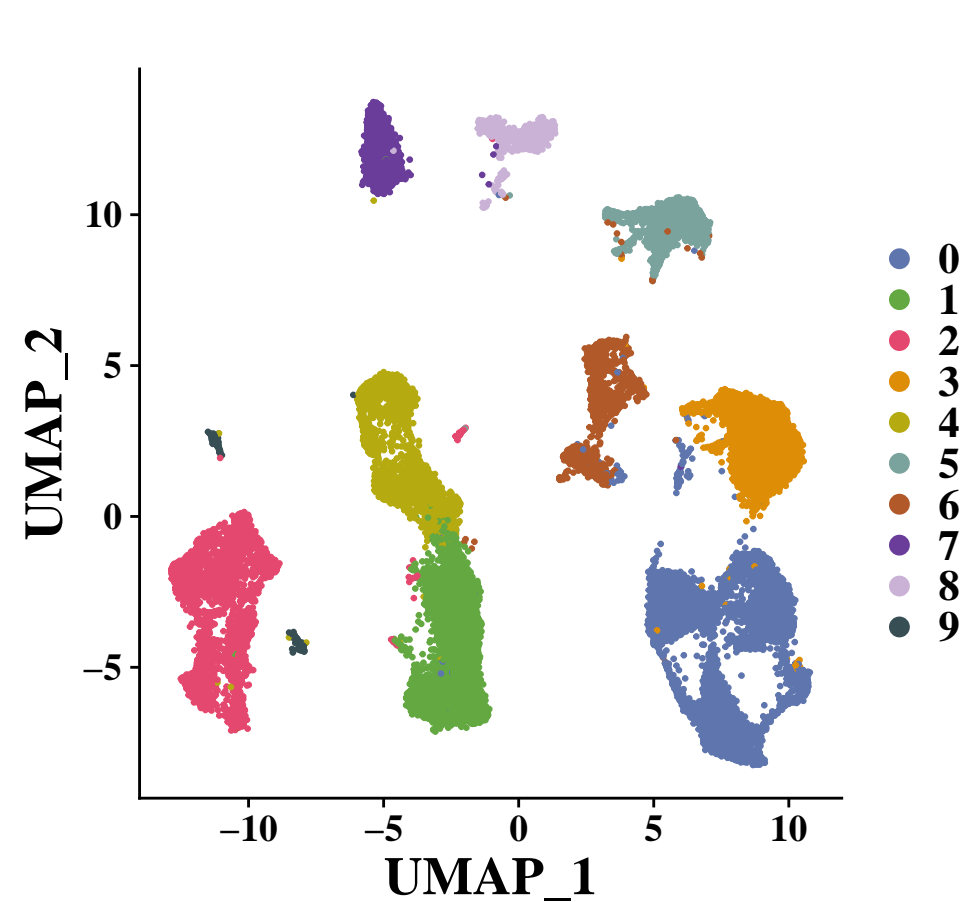

Supplement: Supplementary Table 1 — The primer sequences for PCR. [file DataSheet1.zip › Original data/12_Single_cell/06.UMAP.pdf]

group ARDS

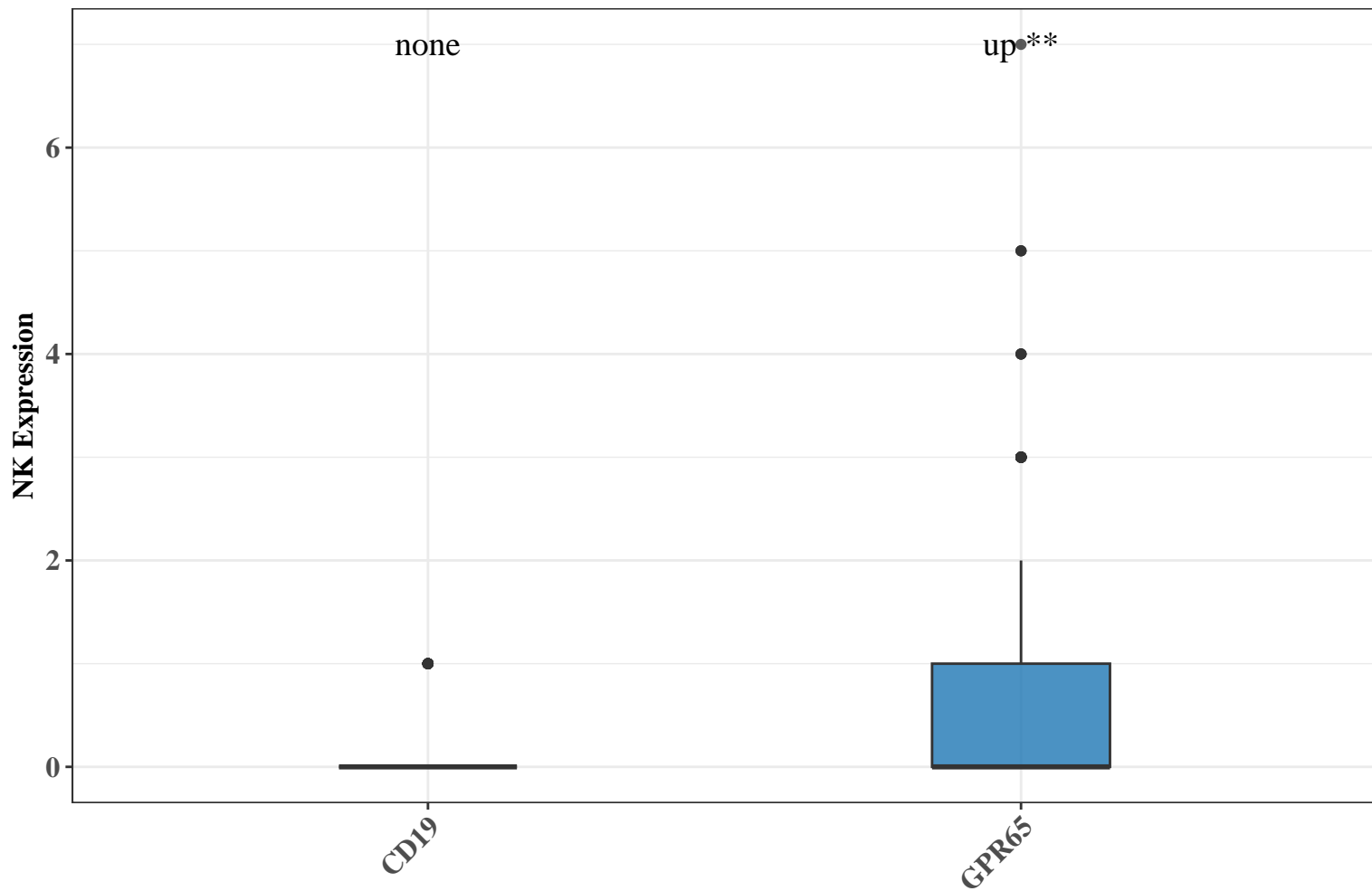

Supplement: Supplementary Table 1 — The primer sequences for PCR. [file DataSheet1.zip › Original data/12_Single_cell/07.keyCell/01.NK_exp.pdf]

group Sepsis ARDS

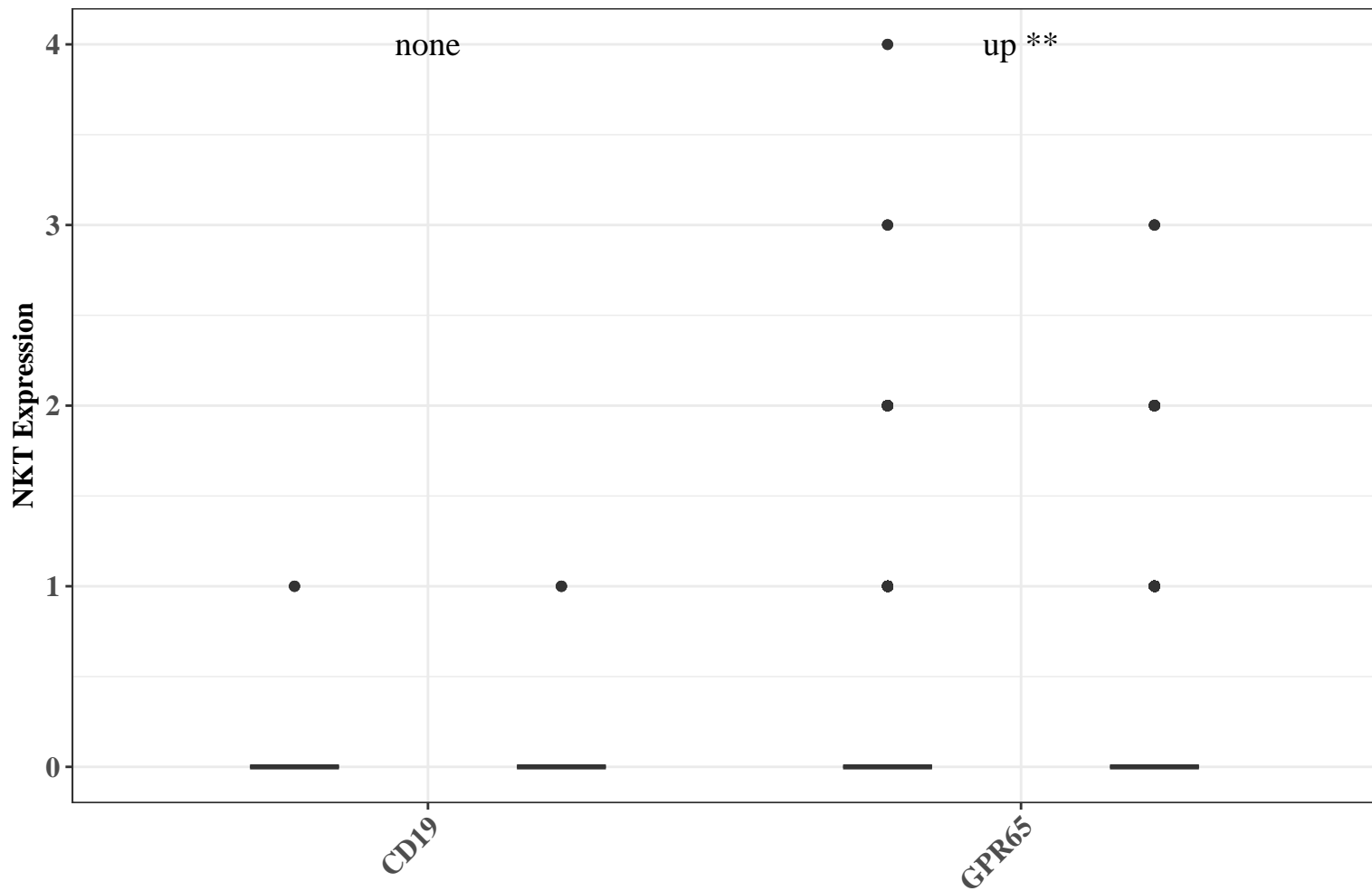

Supplement: Supplementary Table 1 — The primer sequences for PCR. [file DataSheet1.zip › Original data/12_Single_cell/07.keyCell/02.NKT_exp.pdf]

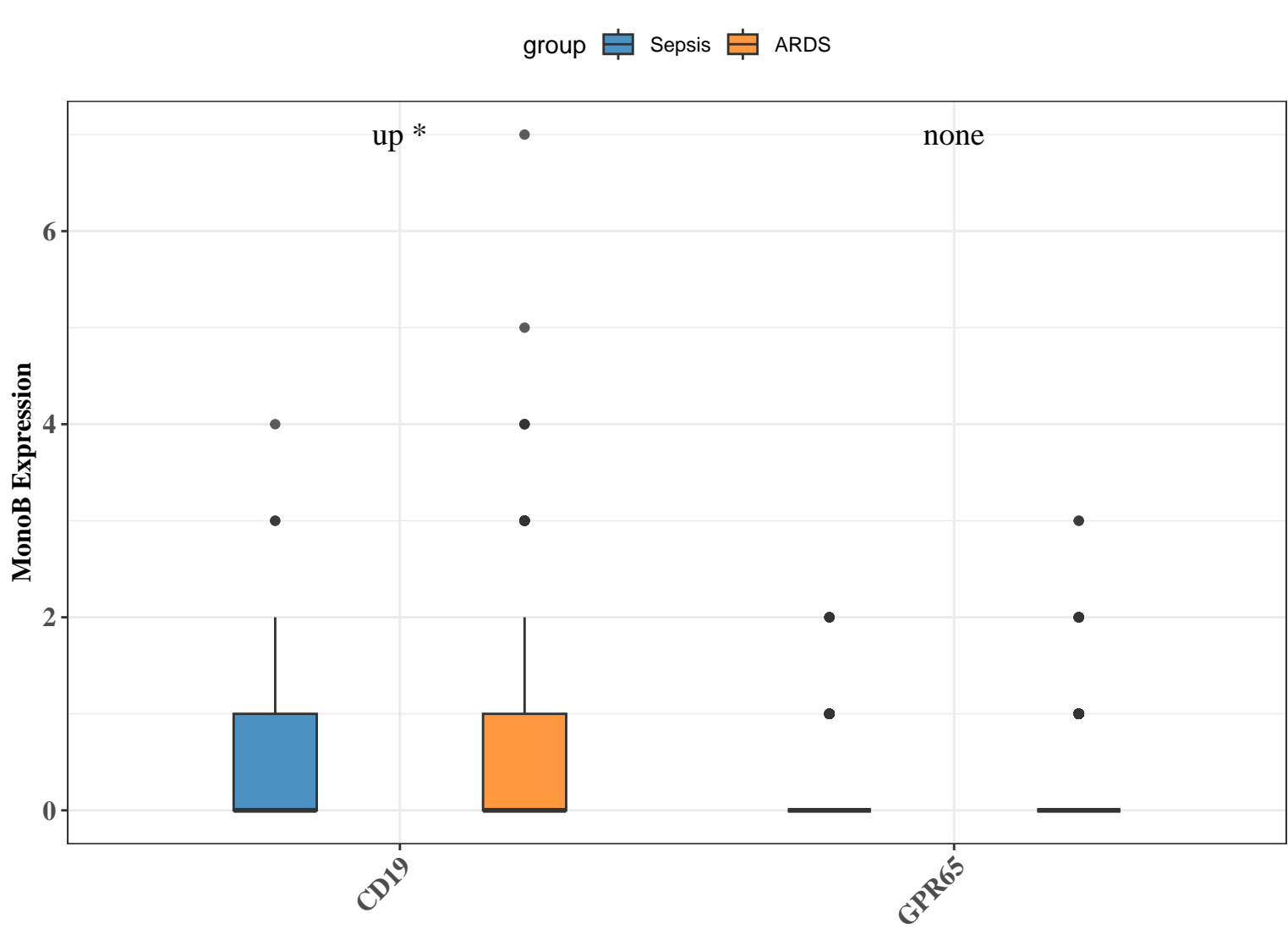

Supplement: Supplementary Table 1 — The primer sequences for PCR. [file DataSheet1.zip › Original data/12_Single_cell/07.keyCell/03.MonoB_exp.pdf]

group Sepsis ARDS

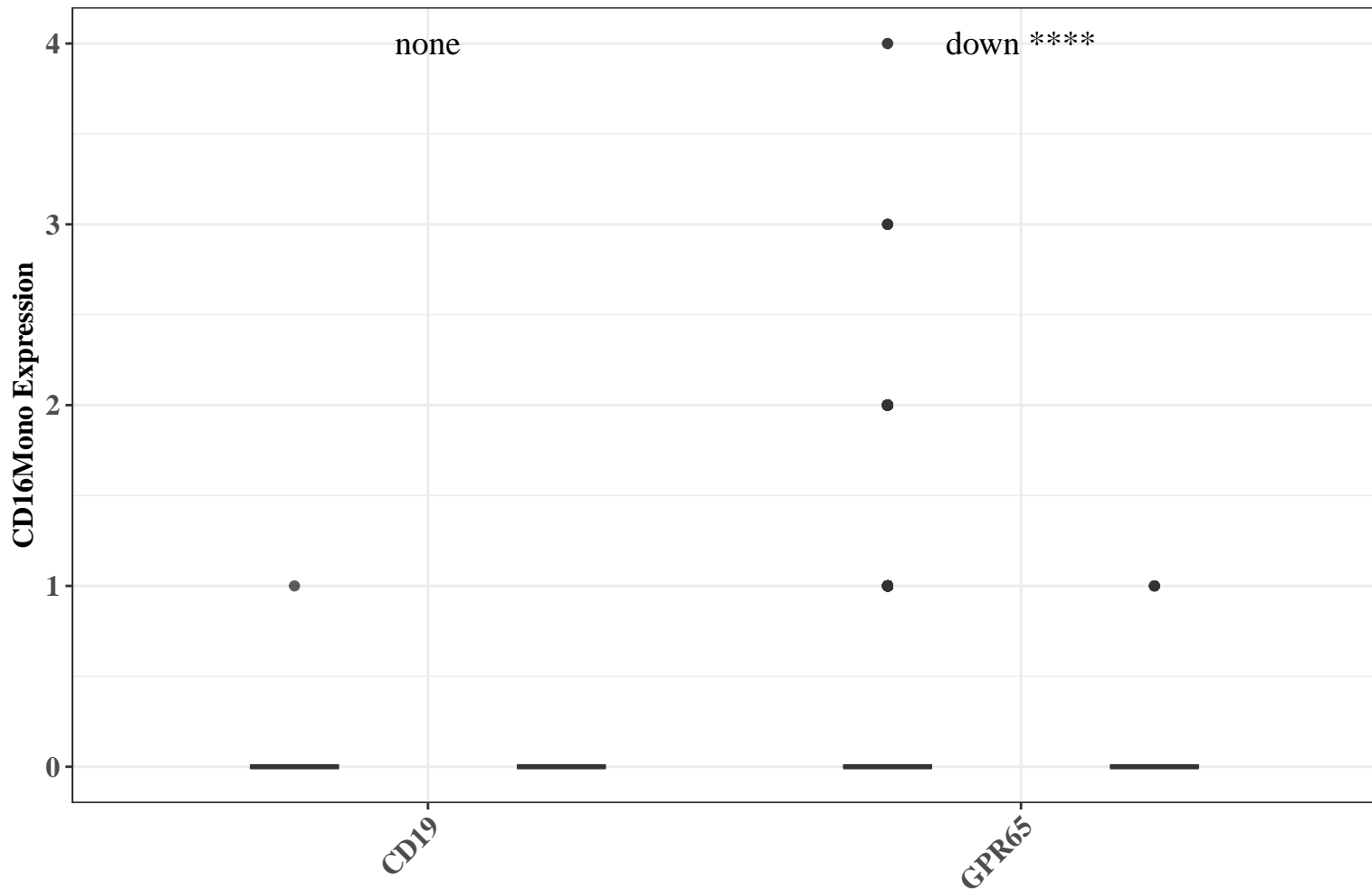

Supplement: Supplementary Table 1 — The primer sequences for PCR. [file DataSheet1.zip › Original data/12_Single_cell/07.keyCell/04.CD16Mono_exp.pdf]

group Sepsis ARDS

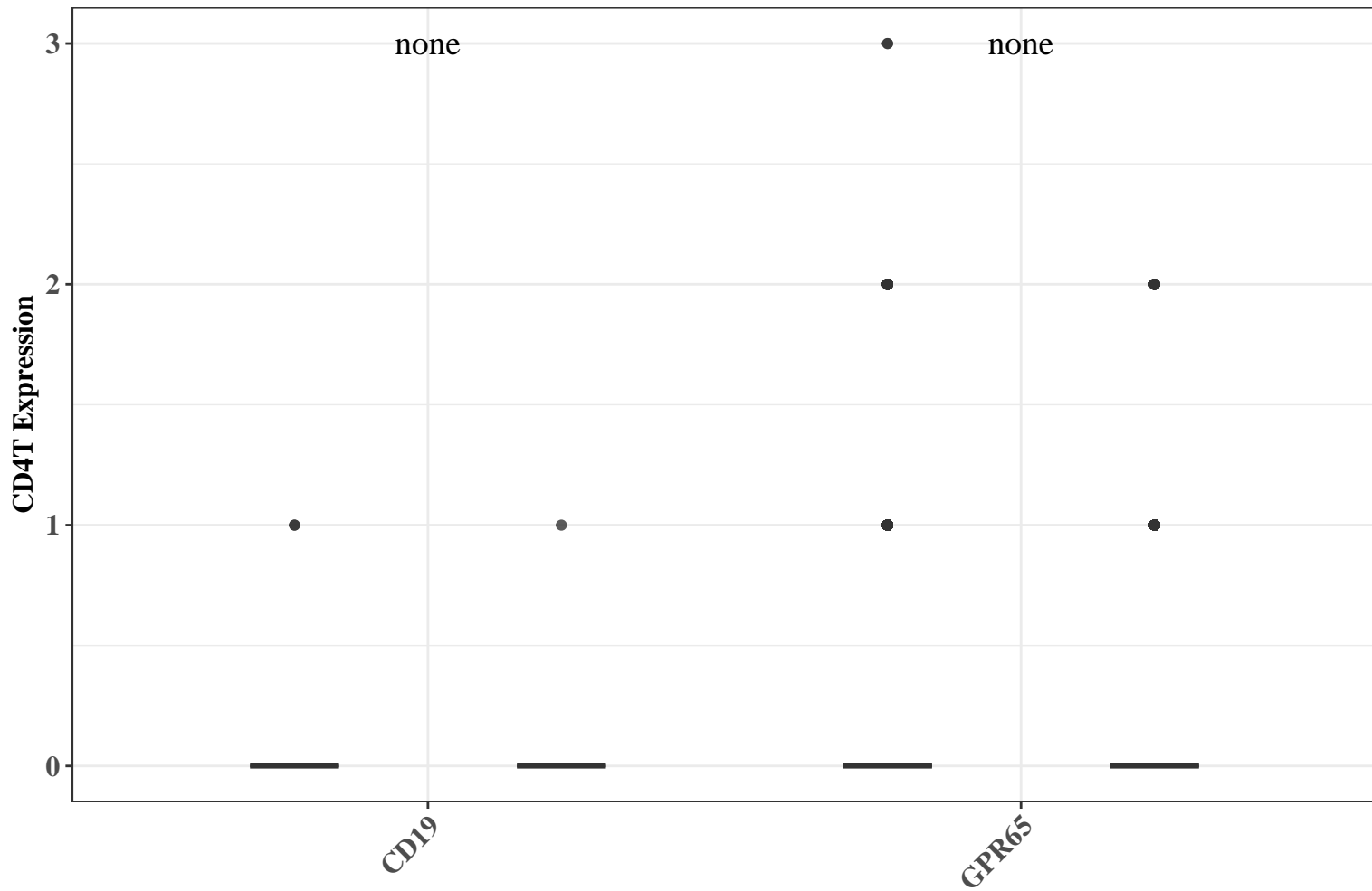

Supplement: Supplementary Table 1 — The primer sequences for PCR. [file DataSheet1.zip › Original data/12_Single_cell/07.keyCell/05.CD4T_exp.pdf]

group Sepsis ARDS

CD14Mono Expression

CD19

GPR65

up \*

down \*\*\*\*

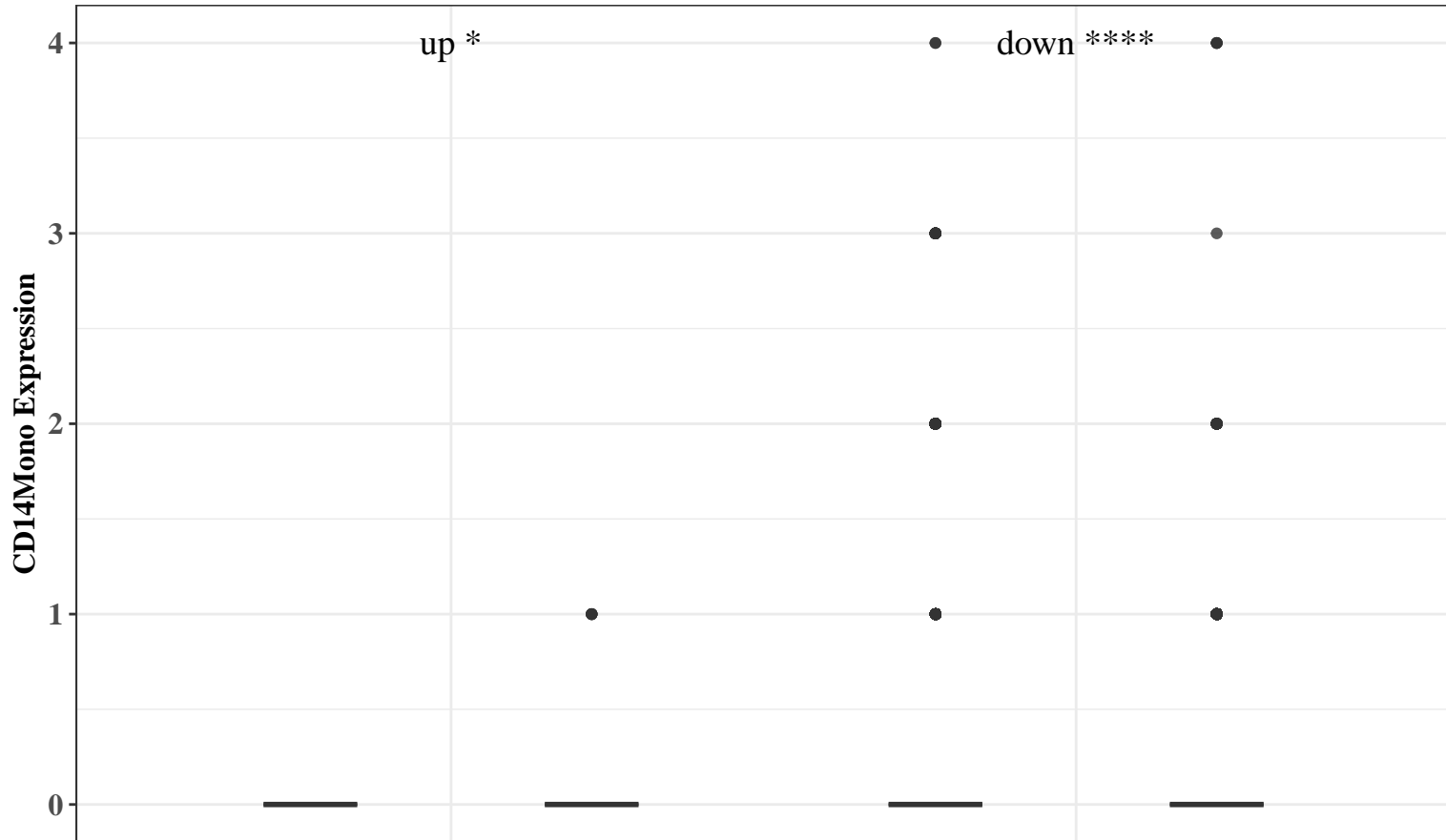

Supplement: Supplementary Table 1 — The primer sequences for PCR. [file DataSheet1.zip › Original data/12_Single_cell/07.keyCell/06.CD14Mono_exp.pdf]

group Sepsis ARDS

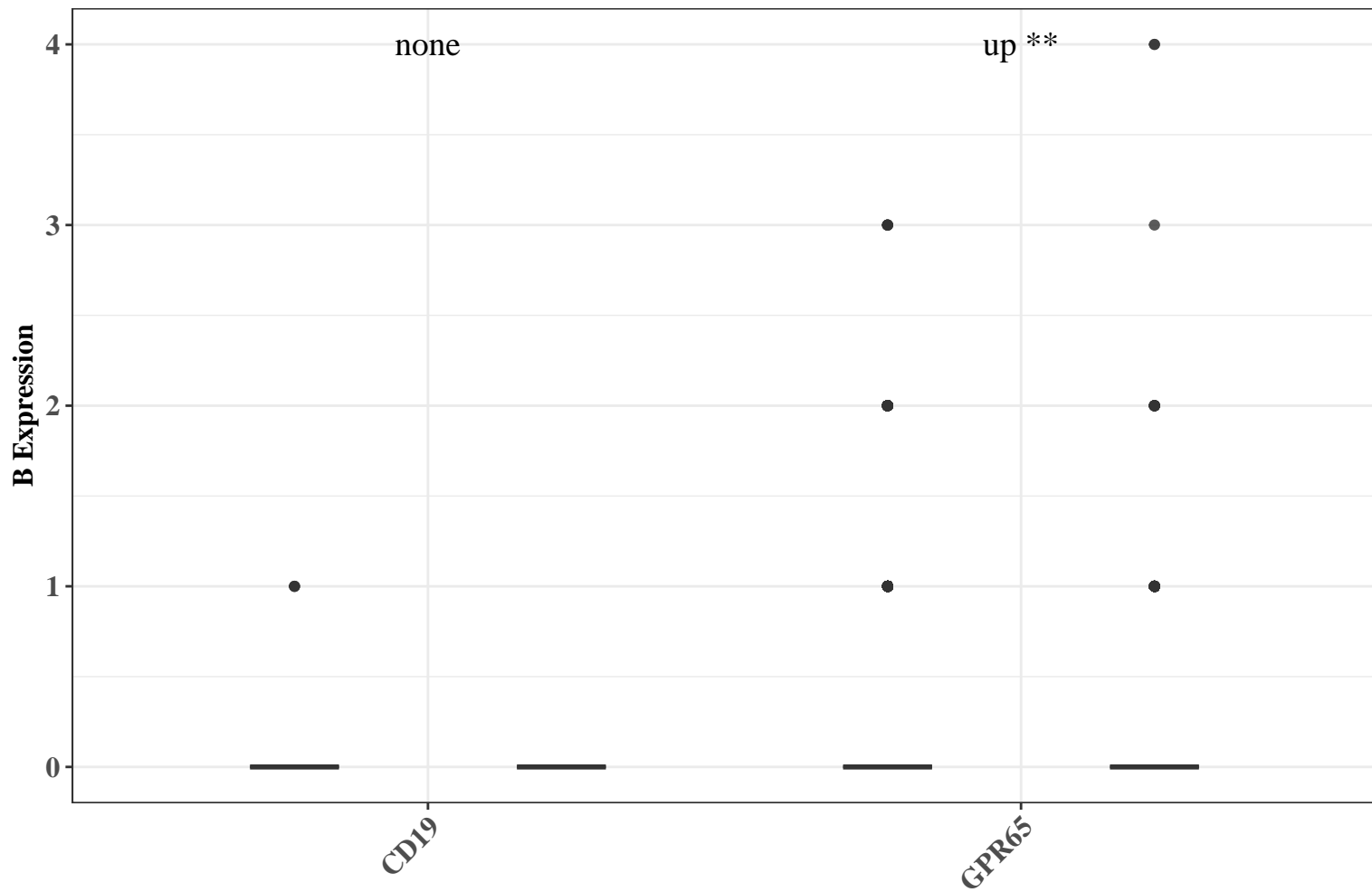

Supplement: Supplementary Table 1 — The primer sequences for PCR. [file DataSheet1.zip › Original data/12_Single_cell/07.keyCell/07.B_exp.pdf]

group Sepsis ARDS

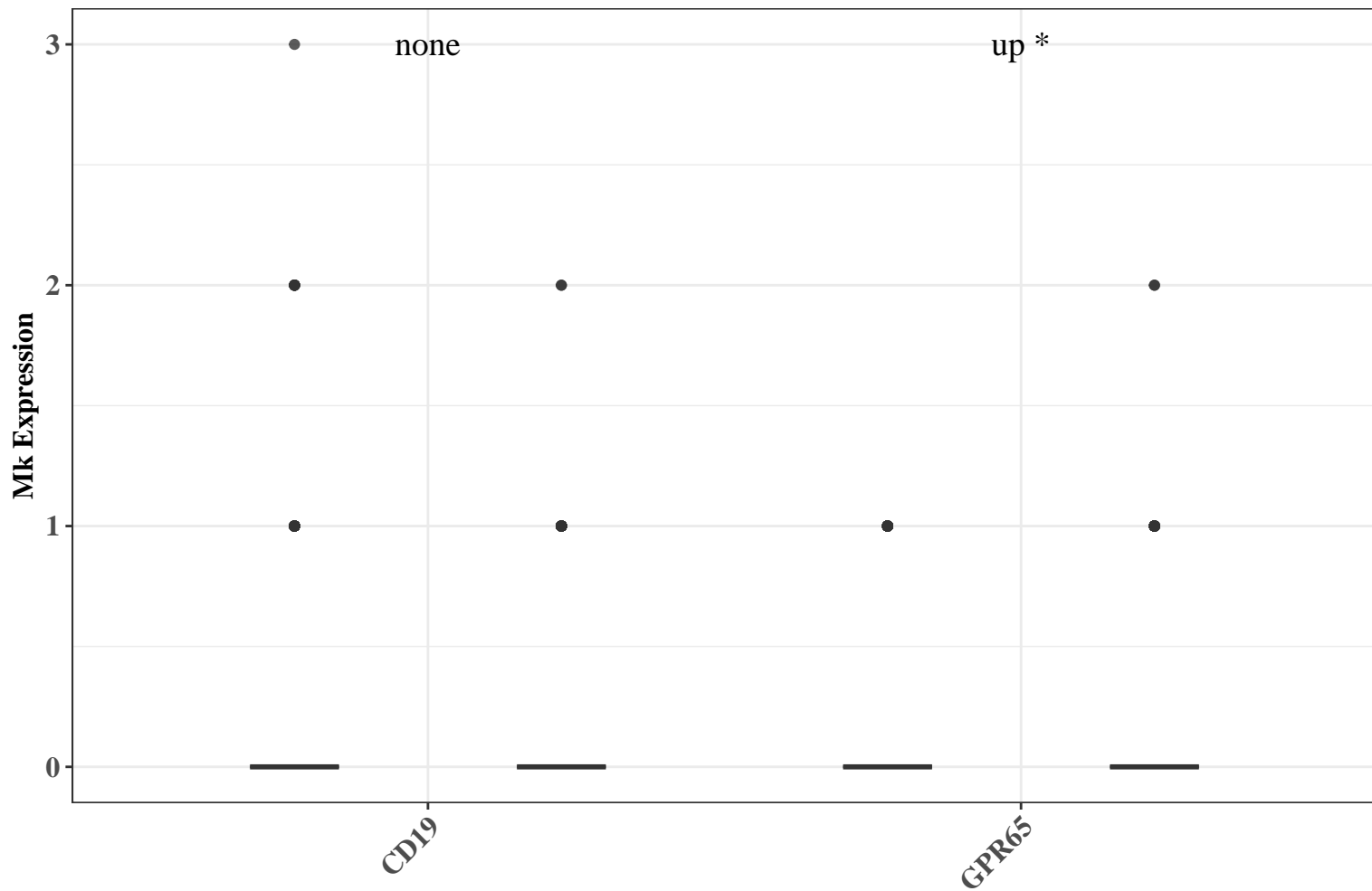

Supplement: Supplementary Table 1 — The primer sequences for PCR. [file DataSheet1.zip › Original data/12_Single_cell/07.keyCell/08.Mk_exp.pdf]

group Sepsis ARDS

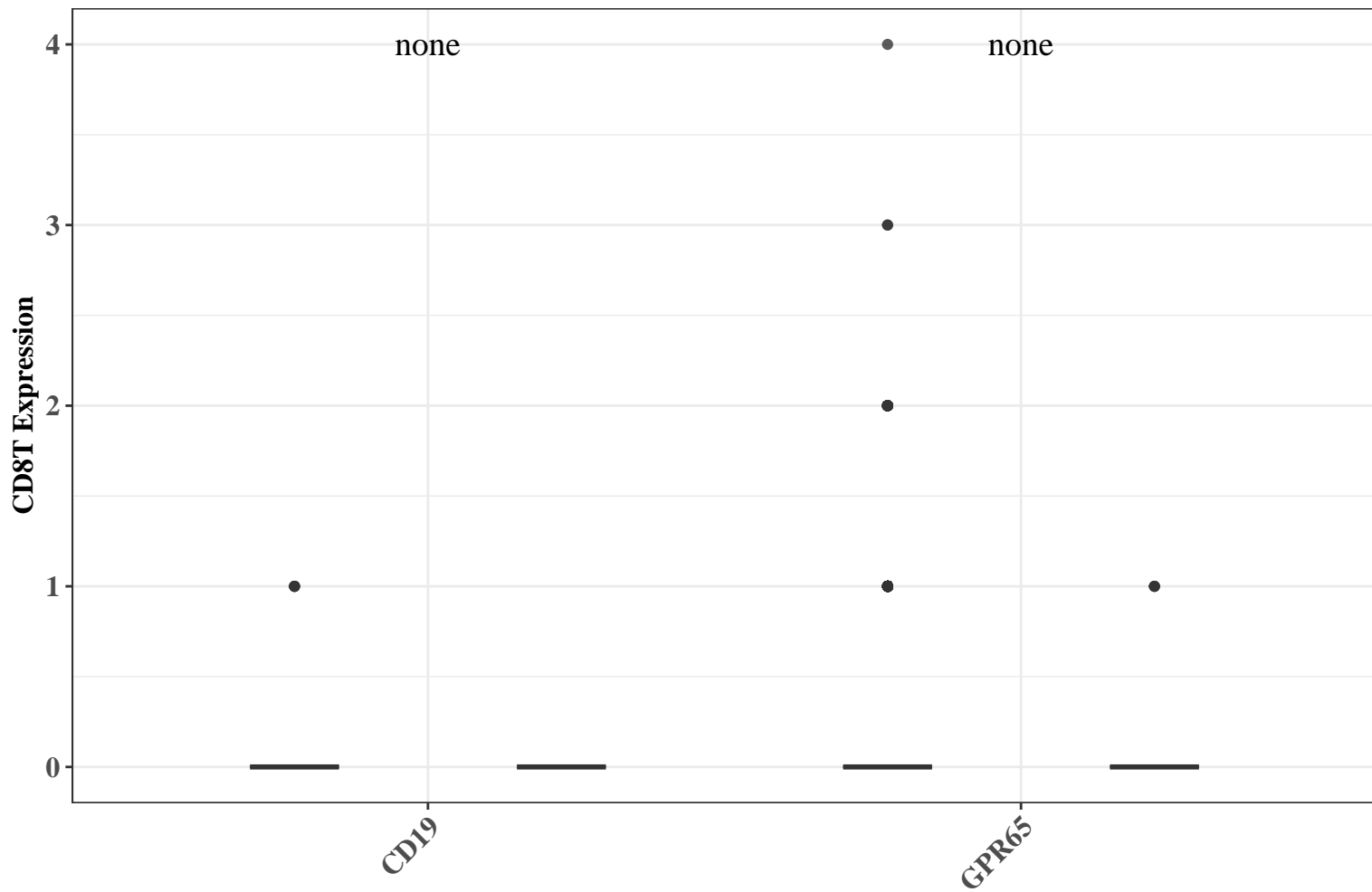

Supplement: Supplementary Table 1 — The primer sequences for PCR. [file DataSheet1.zip › Original data/12_Single_cell/07.keyCell/09.CD8T_exp.pdf]

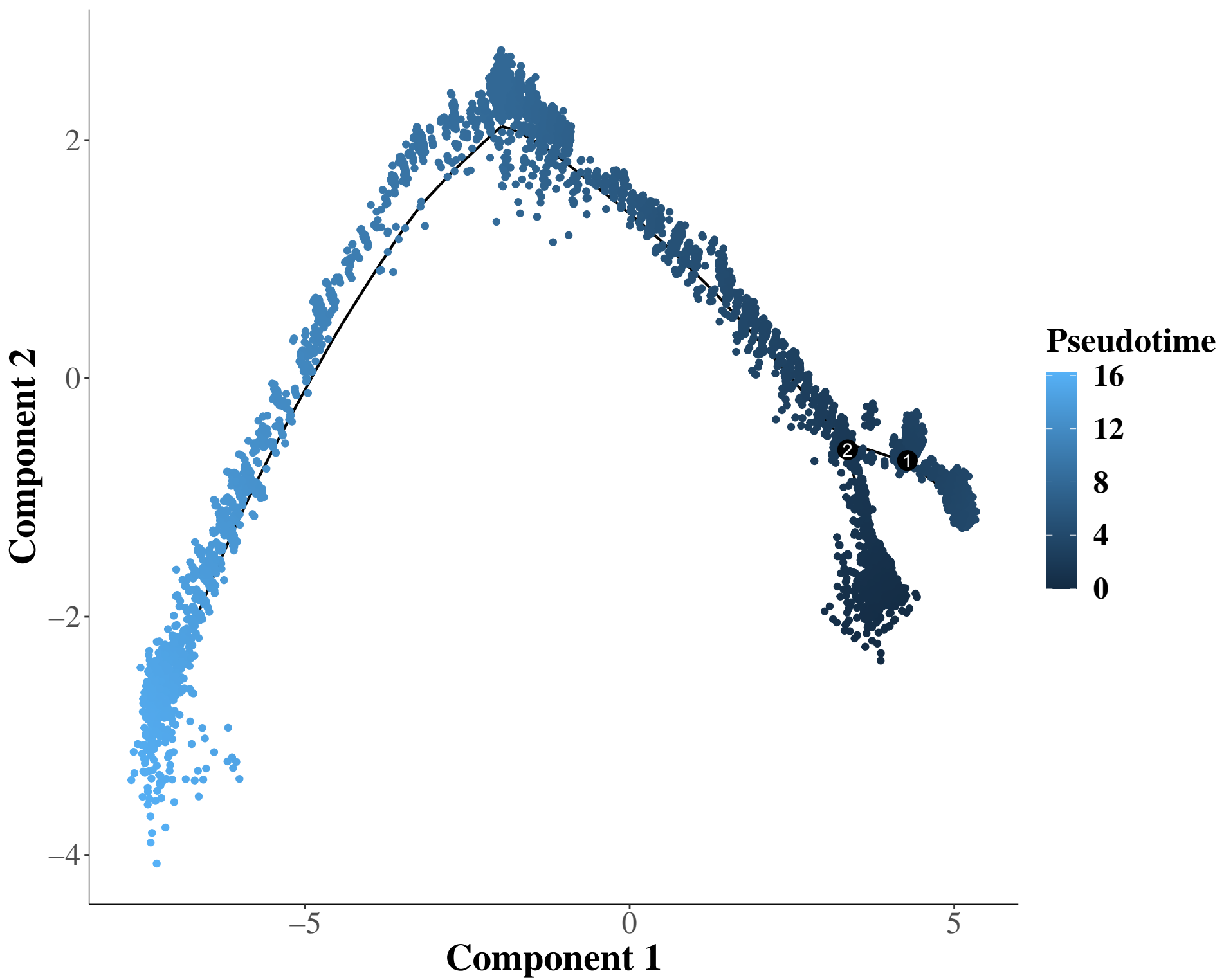

Supplement: Supplementary Table 1 — The primer sequences for PCR. [file DataSheet1.zip › Original data/12_Single_cell/07.keyCell/CD14Mono/13.trajectory_pseudotime_CD14Mono.pdf]

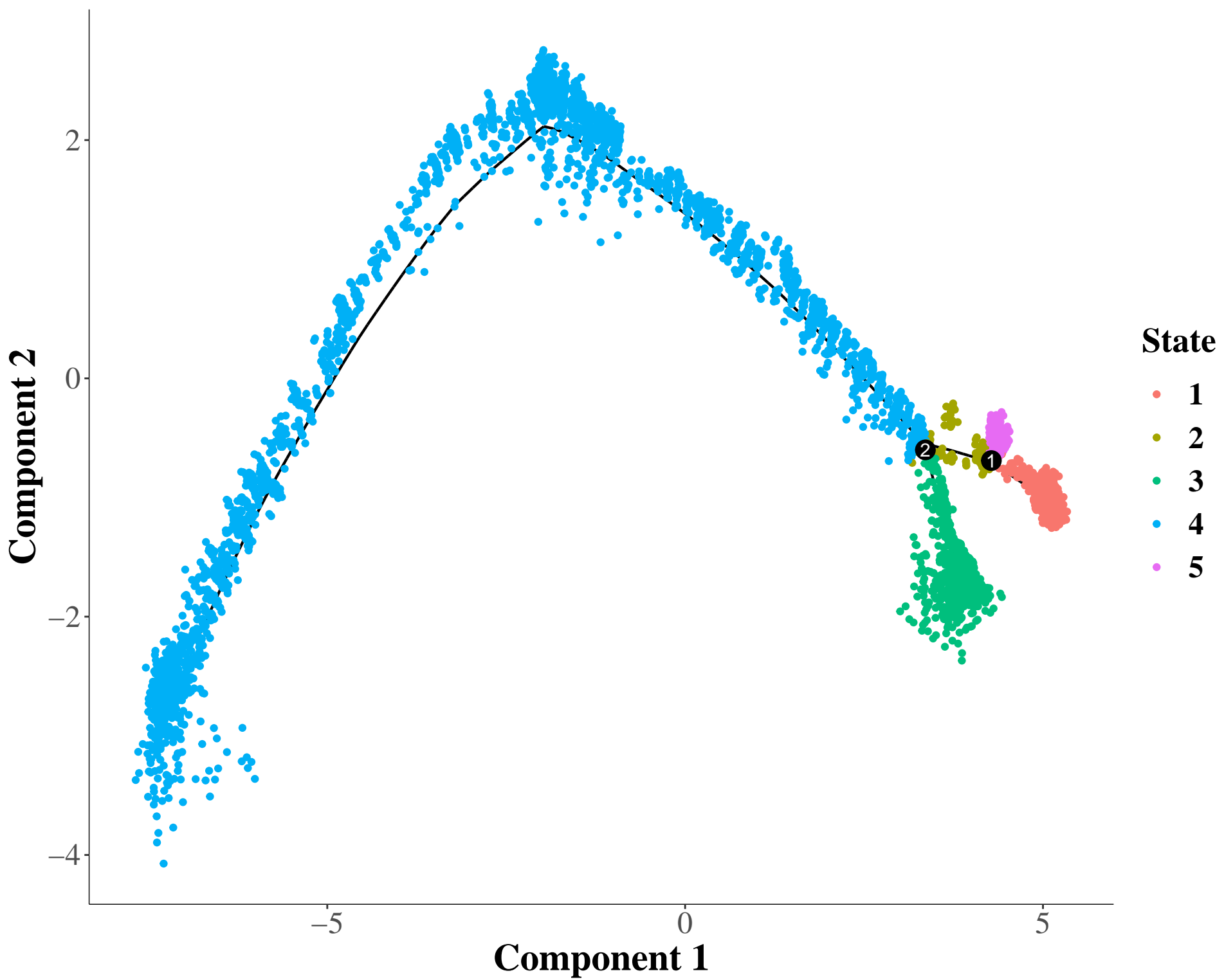

Supplement: Supplementary Table 1 — The primer sequences for PCR. [file DataSheet1.zip › Original data/12_Single_cell/07.keyCell/CD14Mono/14.trajectory_state_CD14Mono.pdf]

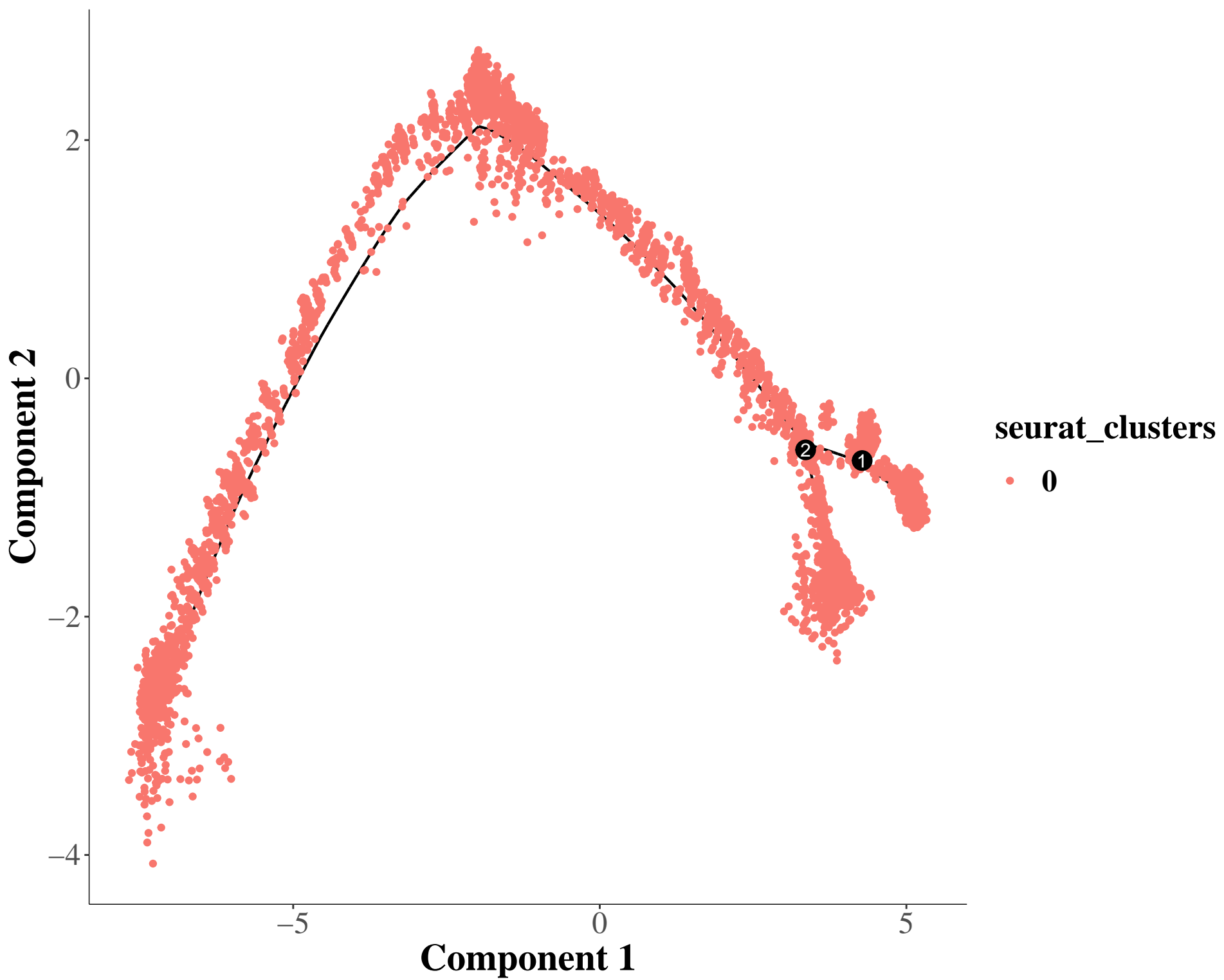

Supplement: Supplementary Table 1 — The primer sequences for PCR. [file DataSheet1.zip › Original data/12_Single_cell/07.keyCell/CD14Mono/15.trajectory_cluster_CD14Mono.pdf]

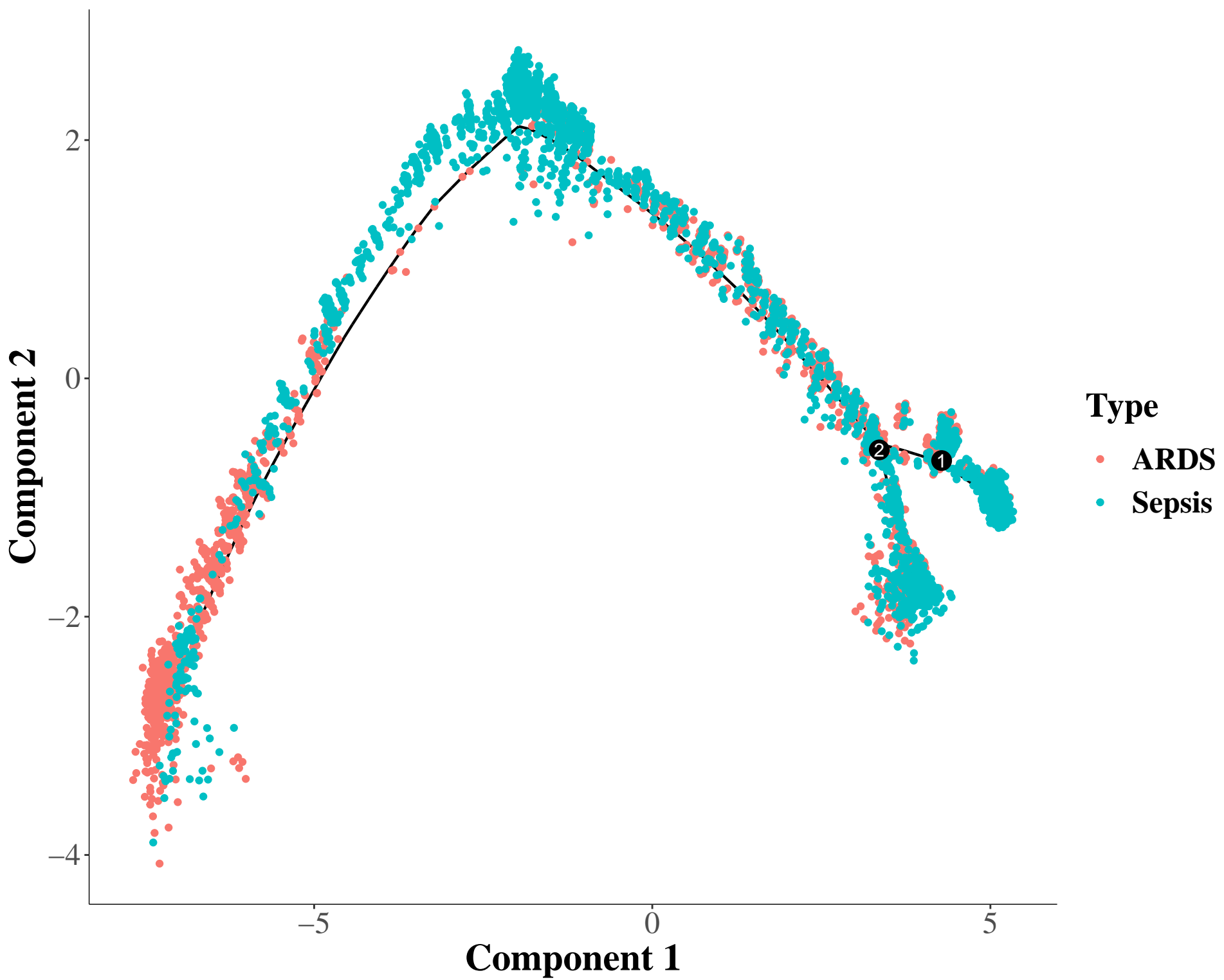

Supplement: Supplementary Table 1 — The primer sequences for PCR. [file DataSheet1.zip › Original data/12_Single_cell/07.keyCell/CD14Mono/16.trajectory_Group_CD14Mono.pdf]

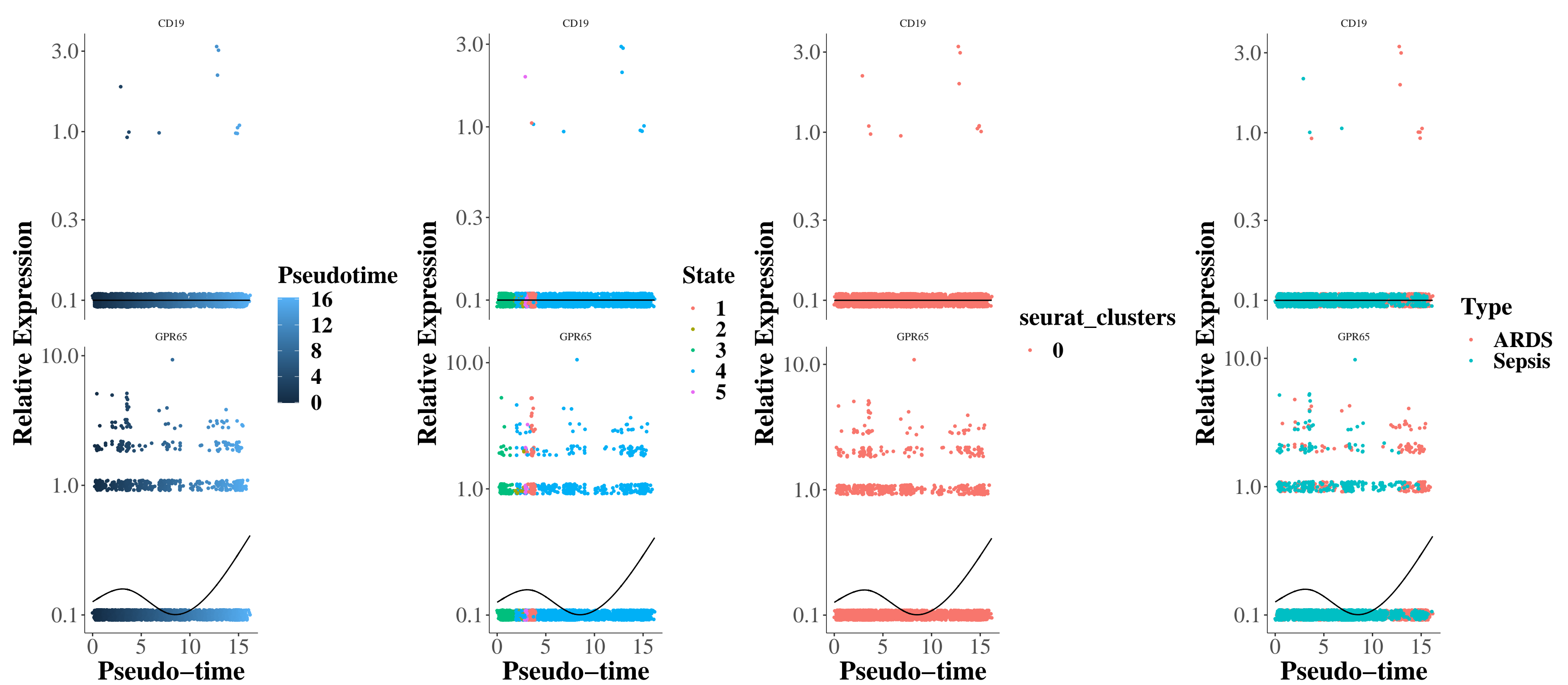

Supplement: Supplementary Table 1 — The primer sequences for PCR. [file DataSheet1.zip › Original data/12_Single_cell/07.keyCell/CD14Mono/17.trajectory_gene_CD14Mono.pdf]

Features

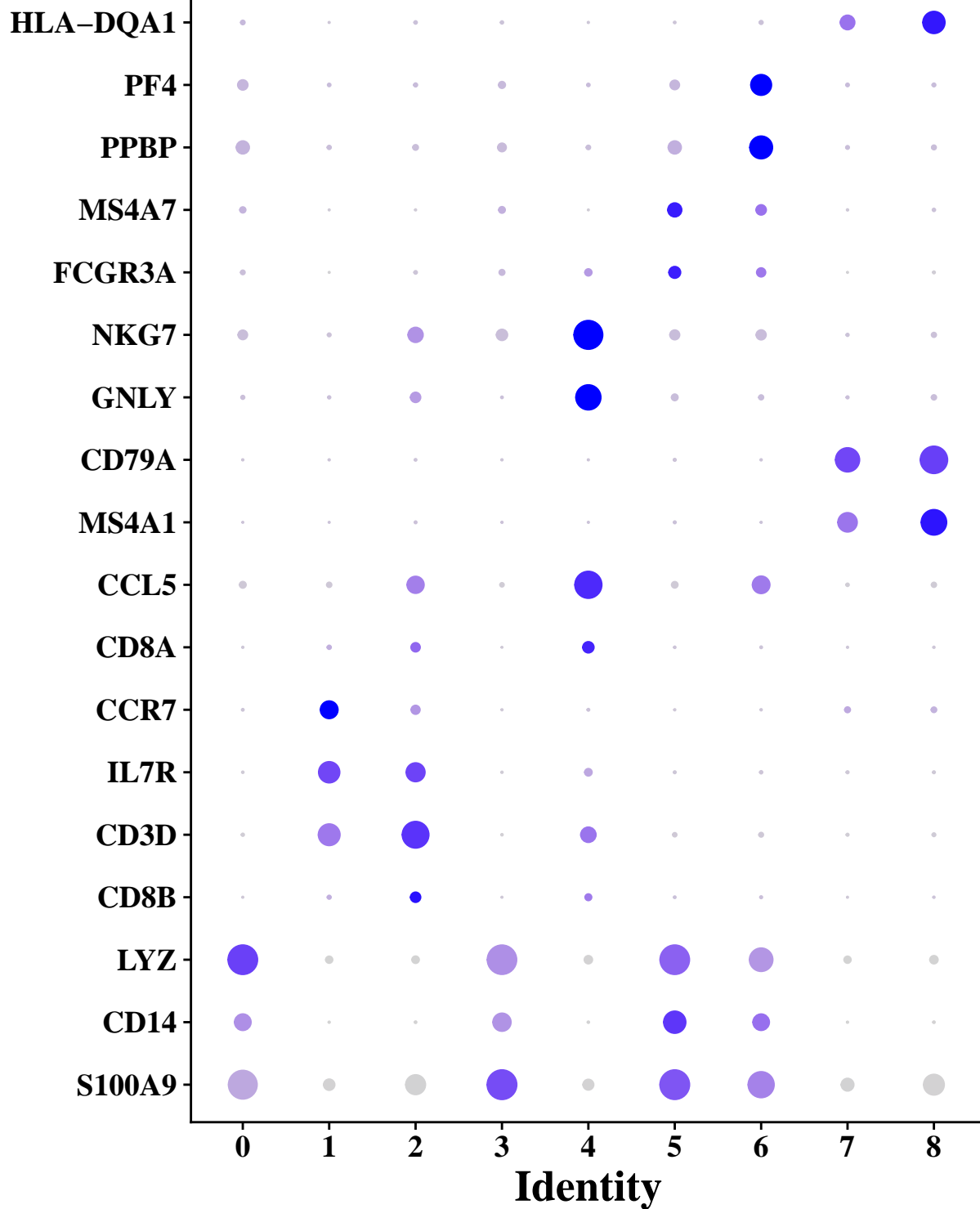

Average Expression

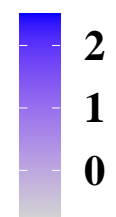

Percent Expressed

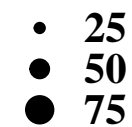

Supplement: Supplementary Table 1 — The primer sequences for PCR. [file DataSheet1.zip › Original data/12_Single_cell/08.DotPlot.pdf]

Features

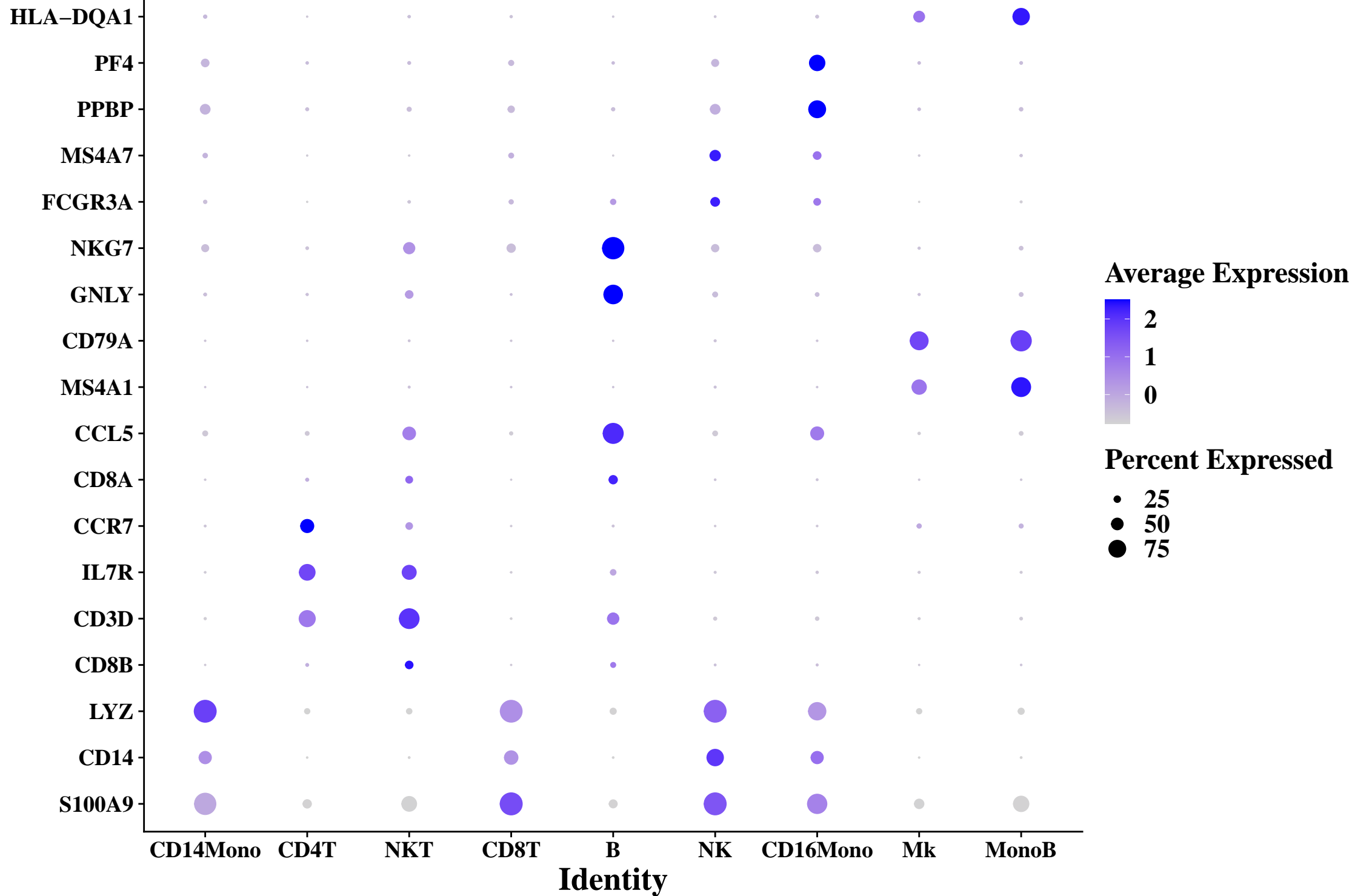

Supplement: Supplementary Table 1 — The primer sequences for PCR. [file DataSheet1.zip › Original data/12_Single_cell/09.DotPlot_celltype.pdf]

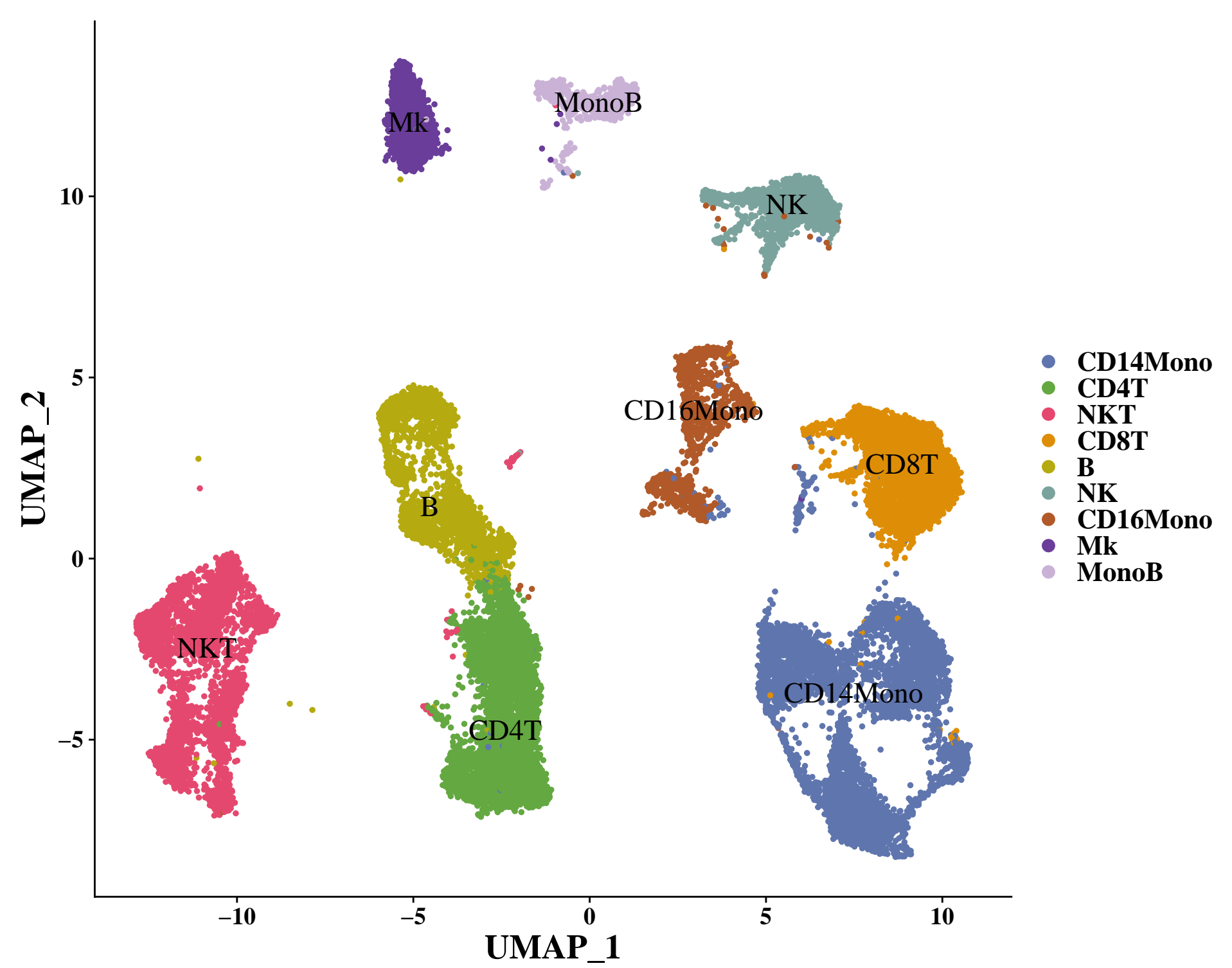

Supplement: Supplementary Table 1 — The primer sequences for PCR. [file DataSheet1.zip › Original data/12_Single_cell/10.Celltype.pdf]

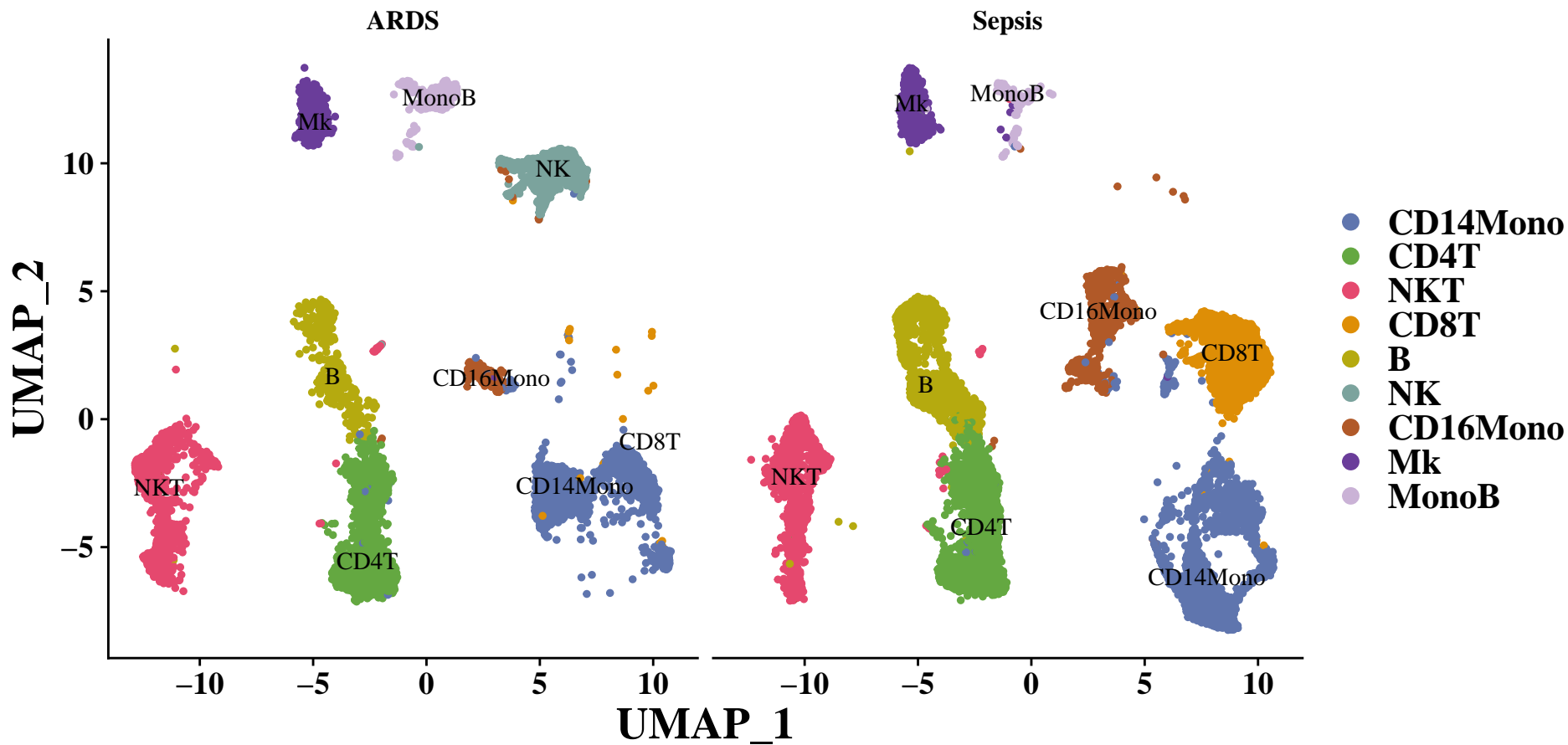

Supplement: Supplementary Table 1 — The primer sequences for PCR. [file DataSheet1.zip › Original data/12_Single_cell/11.Celltype_Group.pdf]

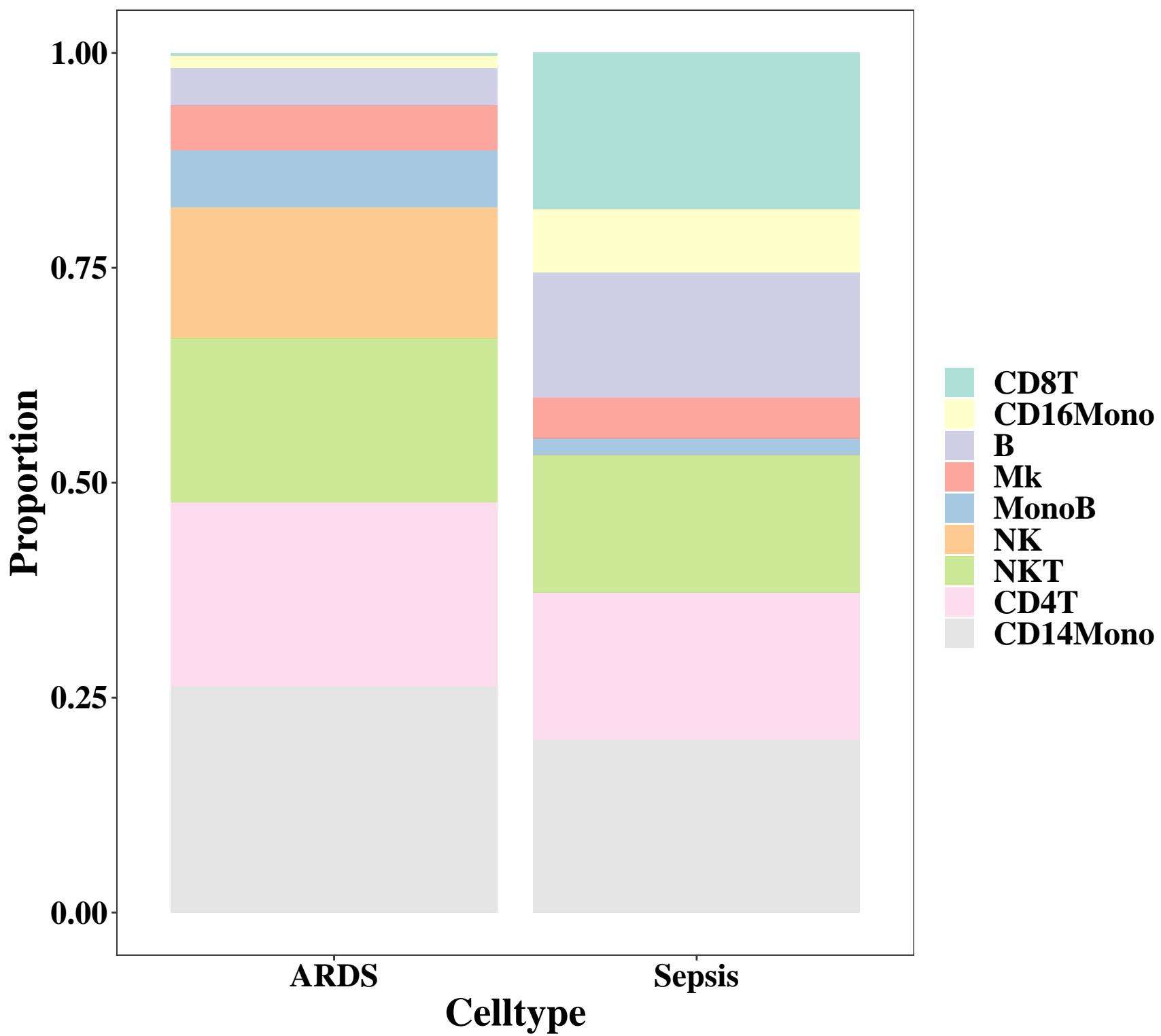

Supplement: Supplementary Table 1 — The primer sequences for PCR. [file DataSheet1.zip › Original data/12_Single_cell/12.Proportion.pdf]

## ARDS

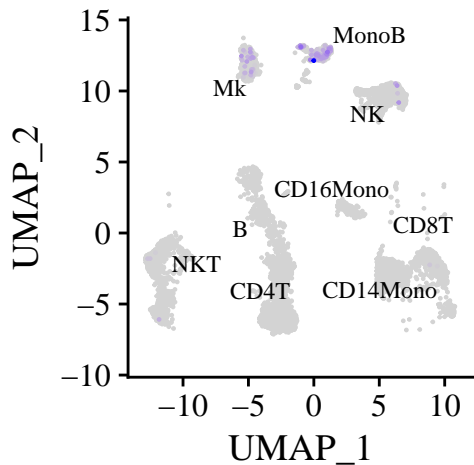

## Sepsis

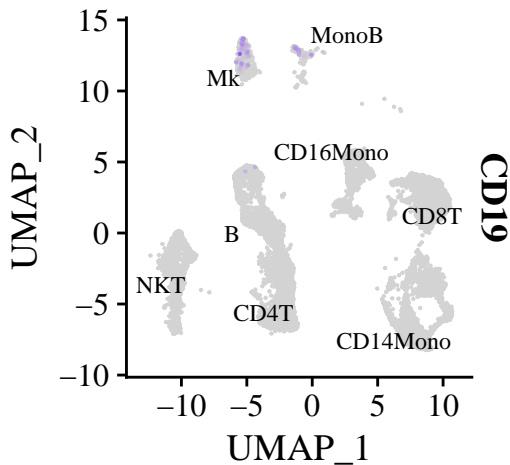

## ARDS

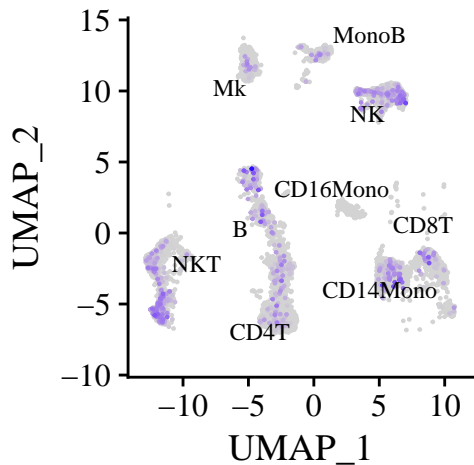

## Sepsis

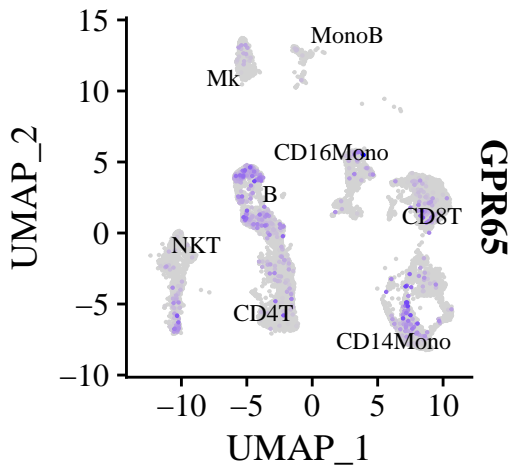

Supplement: Supplementary Table 1 — The primer sequences for PCR. [file DataSheet1.zip › Original data/12_Single_cell/13.FeaturePlot.pdf]
