# Supplementary material for: Integrating single-cell sequencing and transcriptome analysis to unravel the mechanistic role of sialylation-related genes in sepsis-induced acute respiratory distress syndrome
Source: Front Immunol. 2025 May 1;16:1528769. doi: 10.3389/fimmu.2025.1528769 (PMC12078151; doi:10.3389/fimmu.2025.1528769)
Supplement: Supplementary Table 1 — The primer sequences for PCR. [file DataSheet1.zip › Original data/10_Friends/02.GPR65.pdf]

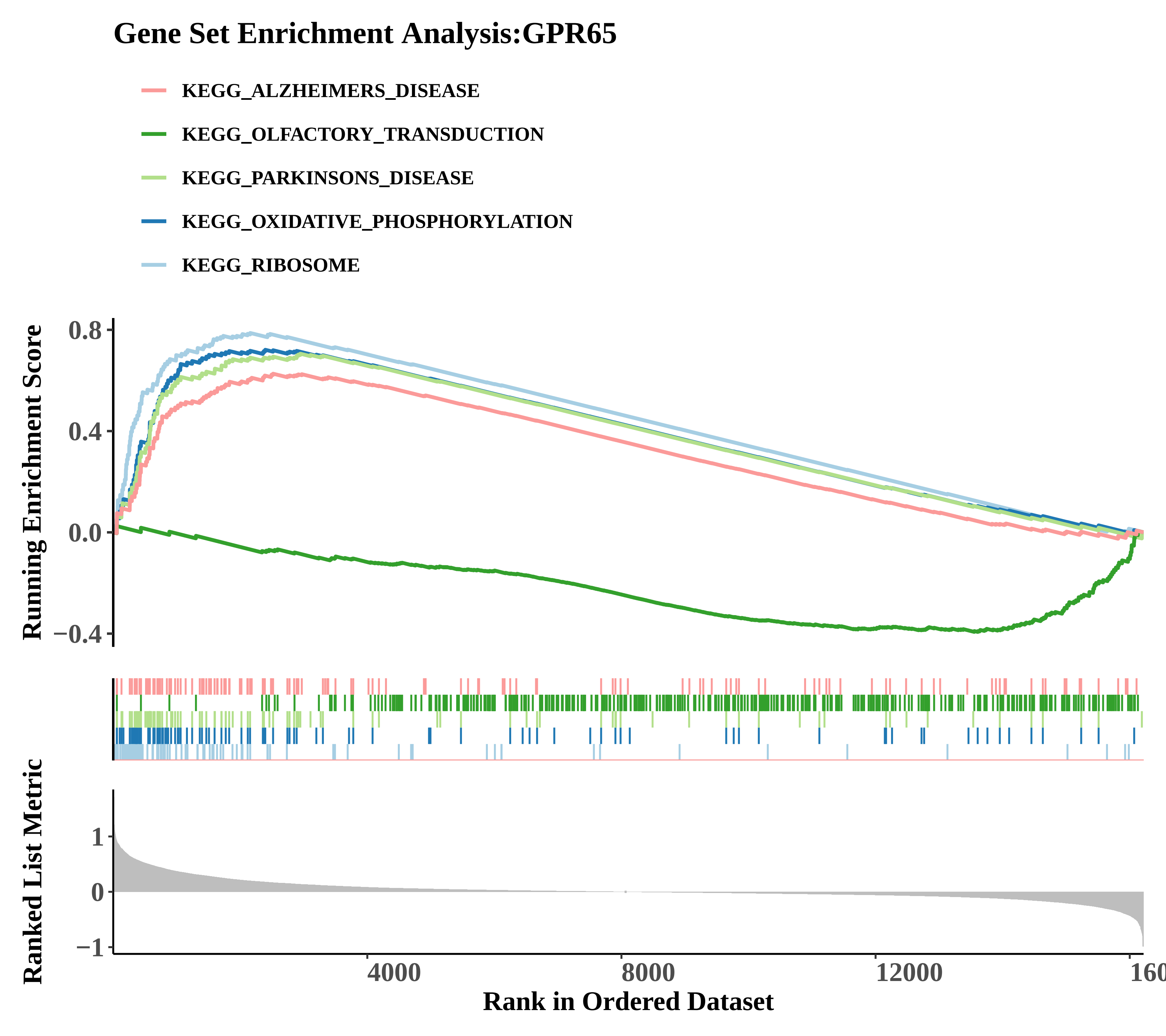
Gene Set Enrichment Analysis:GPR65
KEGG_ALZHEIMERS_DISEASE
KEGG_OLFACTORY_TRANSDUCTION
KEGG_PARKINSONS_DISEASE
KEGG_OXIDATIVE_PHOSPHORYLATION
KEGG_RIBOSOME
Running Enrichment Score
0.8
0.4
0.0
−0.4
Ranked List Metric
1
0
−1
4000
8000
12000
160
Rank in Ordered Dataset
