# Supplementary material for: Integrating single-cell sequencing and transcriptome analysis to unravel the mechanistic role of sialylation-related genes in sepsis-induced acute respiratory distress syndrome
Source: Front Immunol. 2025 May 1;16:1528769. doi: 10.3389/fimmu.2025.1528769 (PMC12078151; doi:10.3389/fimmu.2025.1528769)
Supplement: Supplementary Table 1 — The primer sequences for PCR. [file DataSheet1.zip › Original data/10_Friends/genemania-report.pdf]

GeneMANIA report

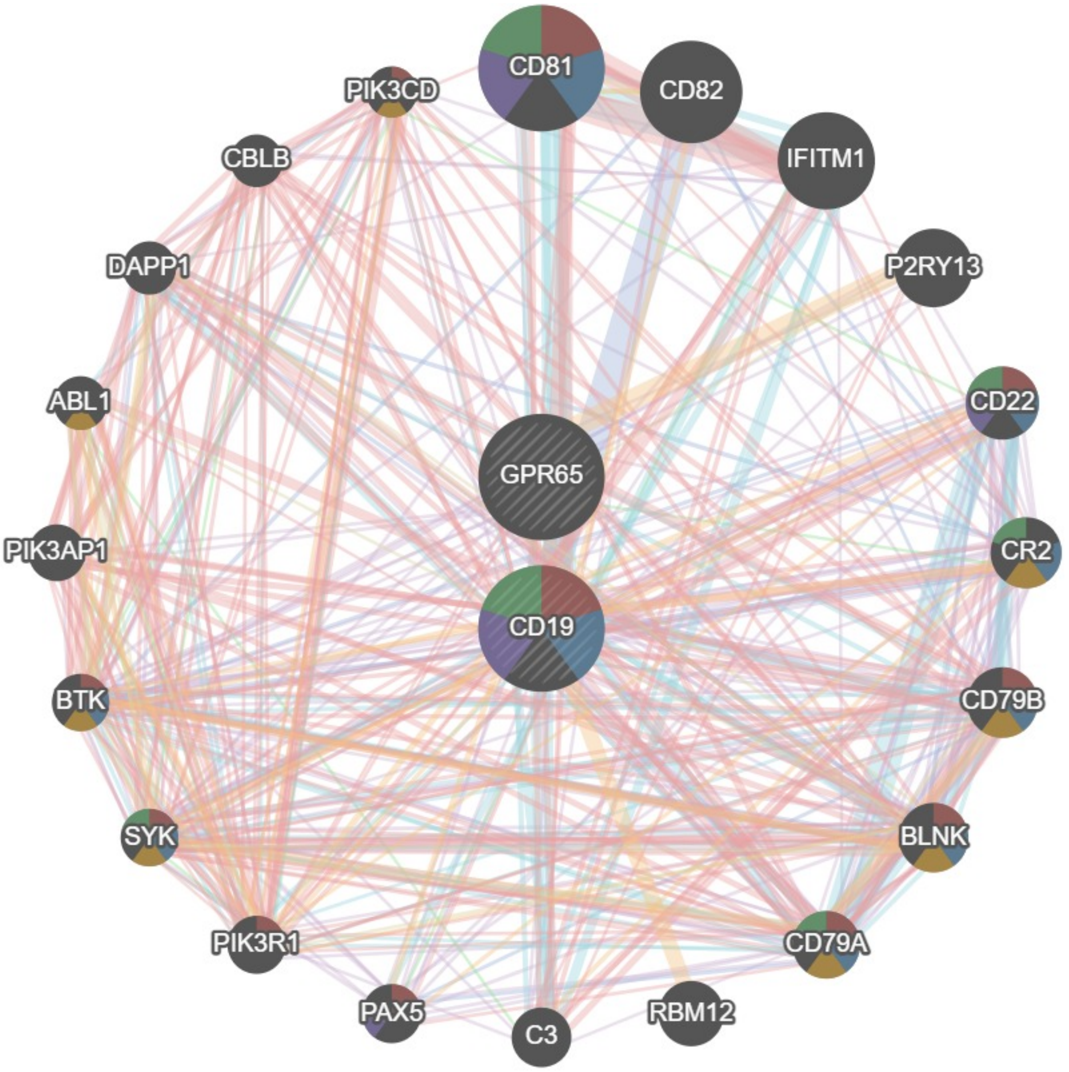

Networks

- Physical Interactions
- Co-expression
- Predicted
- Co-localization
- Genetic Interactions
- Pathway
- Shared protein domains

Functions

- antigen receptor-mediated signaling pathway
- B cell activation
- lymphocyte differentiation
- B cell receptor signaling pathway
- lymphocyte proliferation
