# Supplementary material for: Integrating single-cell sequencing and transcriptome analysis to unravel the mechanistic role of sialylation-related genes in sepsis-induced acute respiratory distress syndrome
Source: Front Immunol. 2025 May 1;16:1528769. doi: 10.3389/fimmu.2025.1528769 (PMC12078151; doi:10.3389/fimmu.2025.1528769)
Supplement: Supplementary file 5 [file Table1.docx]

| **Primer** | **Sequence** | |
| --- | --- | --- |
| CD19 F | CCAGAACCAGTACGGGAACG | |
| CD19 R | CTCGGGTTTCCATAAGACGGG | |
| GPR65 F | CGTGTTCTGTACGGGACCTC | |
| GPR65 R | TCCTGGGAGGGACTTCTTGT | |
| GAPDH F | CGAAGGTGGAGTCAACGGATTT |  |
| GAPDH R | ATGGGTGGAATCATATTGGAAC |  |
